# High-Throughput Sequencing and Unsupervised Analysis of Formyltetrahydrofolate Synthetase (FTHFS) Gene Amplicons to Estimate Acetogenic Community Structure

**DOI:** 10.3389/fmicb.2020.02066

**Published:** 2020-08-27

**Authors:** Abhijeet Singh, Johan A. A. Nylander, Anna Schnürer, Erik Bongcam-Rudloff, Bettina Müller

**Affiliations:** ^1^Anaerobic Microbiology and Biotechnology Group, Department of Molecular Sciences, Swedish University of Agricultural Sciences, Uppsala, Sweden; ^2^Department of Bioinformatics and Genetics, Swedish Museum of Natural History, Stockholm, Sweden; ^3^National Bioinformatics Infrastructure Sweden, SciLifeLab, Uppsala, Sweden; ^4^SLU-Global Bioinformatics Centre, Department of Animal Breeding and Genetics, Swedish University of Agricultural Sciences, Uppsala, Sweden

**Keywords:** FTHFS, acetogens, AcetoScan, AcetoBase, high-throughput sequencing

## Abstract

The formyltetrahydrofolate synthetase (FTHFS) gene is a molecular marker of choice to study the diversity of acetogenic communities. However, current analyses are limited due to lack of a high-throughput sequencing approach for FTHFS gene amplicons and a dedicated bioinformatics pipeline for data analysis, including taxonomic annotation and visualization of the sequence data. In the present study, we combined the barcode approach for multiplexed sequencing with unsupervised data analysis to visualize acetogenic community structure. We used samples from a biogas digester to develop proof-of-principle for our combined approach. We successfully generated high-throughput sequence data for the partial FTHFS gene and performed unsupervised data analysis using the novel bioinformatics pipeline “AcetoScan” presented in this study, which resulted in taxonomically annotated OTUs, phylogenetic tree, abundance plots and diversity indices. The results demonstrated that high-throughput sequencing can be used to sequence the FTHFS amplicons from a pool of samples, while the analysis pipeline AcetoScan can be reliably used to process the raw sequence data and visualize acetogenic community structure. The method and analysis pipeline described in this paper can assist in the identification and quantification of known or potentially new acetogens. The AcetoScan pipeline is freely available at https://github.com/abhijeetsingh1704/AcetoScan.

## Introduction

Acetogens are a group of bacteria that (1) use the Wood-Ljungdahl pathway (WLP) for energy conservation, (2) generate acetyl-Coenzyme A (CoA) by reduction of two molecules of carbon dioxide (CO_2_), (3) may or may not produce acetate as the main end product of carbon fixation by WLP and (4) are obligate anaerobes, with some members having tolerance to periods of aerobiosis (Wagner et al., 1996; Drake and Küsel, 2003; Küsel and Drake, 2011; Schuchmann and Müller, 2016). WLP is one of the most ancient biological pathways known (Pereló et al., 1999; Russell and Martin, 2004). It is used mainly by acetogens, but also by some archaea, syntrophic acetate-oxidizing bacteria and sulfate-reducing bacteria (Drake, 1994; Drake et al., 1997; Ragsdale and Pierce, 2008; Poehlein et al., 2012; Sakimoto et al., 2016). However, only acetogens use the complete WLP and meet the requirements for reductive or true acetogenesis (Drake, 1994; Poehlein et al., 2012; Müller and Frerichs, 2013). Acetogens are phylogenetically very diverse and consist of more than 100 species in more than 23 genera (Drake et al., 2013; Müller and Frerichs, 2013; Shin et al., 2016). They are metabolically very dexterous and, in addition to acetate, may produce ethanol, butyrate, lactate *etc.* (Das and Ljungdahl, 2003; Lovell and Leaphart, 2005; Hügler and Sievert, 2011; Yang, 2018). They are also very competitive when co-existing with methanogenic archaea and sulfate-reducing bacteria, especially if the temperature is below 20°C (Drake, 1994; Kotsyurbenko et al., 1996; Dar et al., 2008; Drake et al., 2013; Fu et al., 2019; Palacios et al., 2019). Acetogens play a very important role in biological carbon cycling and produce approximately 10^13^ kg/annum of acetate in different anaerobic environments worldwide (Müller, 2003; Lovell and Leaphart, 2005; Ragsdale, 2007; Ragsdale and Pierce, 2008; Schuchmann and Müller, 2016). For example, acetogens are one of the most dominant microorganisms in the human gut (Sagheddu et al., 2017), where they generate approximately 10^10^ kg of acetate annually worldwide by reductive acetogenesis. In the termite gut, acetogenesis results in annual worldwide acetate turnover of approximately 10^12^ kg (Breznak and Kane, 1990; Drake et al., 2013). A further approximately 10^12^ kg of acetate is produced in different terrestrial and marine environments like forest soil, marshes, peat and sediments *etc.* (e.g., King, 1991; Kusel and Drake, 1994; Drake et al., 2013). However, due to lack of sufficient data from other environments with prominent acetogenesis, the actual amount of acetate production globally cannot be resolved (Küsel and Drake, 2011; Drake et al., 2013). Acetogens are also of importance in constructed anaerobic digestion systems, where they are important key players for efficient and stable methane production (e.g., Xu et al., 2009; Weiland, 2010; Hori et al., 2011; Wang et al., 2013; Moestedt et al., 2016; Müller et al., 2016; Westerholm et al., 2018; Fischer et al., 2019).

This physiological diversity and versatility enables acetogens to survive in different types of habitats and compete with other microorganisms (Drake et al., 1997; Drake and Küsel, 2003). However, the phylogenetic heterogeneity of acetogens hampers their detection and identification based on the most commonly used molecular markers. For example, designing acetogen-specific primers for the 16S rRNA gene is almost impossible (Drake, 1994; Drake et al., 2002, 2008, 2013; Lovell and Leaphart, 2005). Enzymes involved in the WLP, especially formyltetrahydrofolate synthetase (FTHFS), are structurally and functionally conserved and the gene sequences of these enzymes have been used in numerous studies to target and identify potential acetogens in complex microbial communities (e.g., Lovell and Hui, 1991; Ragsdale and Wood, 1991; Lovell, 1994; Leaphart and Lovell, 2001; Lovell and Leaphart, 2005; Ragsdale and Pierce, 2008; Hori et al., 2011; Müller et al., 2013, 2016; Shin et al., 2016). Despite this, a high-throughput analysis pipeline for this group of organisms has not yet been established. Most, if not all, previous studies using FTHFS gene amplicons have been conducted by clone library construction, sequencing by low-throughput methods and manually evaluated against small reference datasets (Gagen et al., 2010; Henderson et al., 2010; Hori et al., 2011; Planý et al., 2019). This process is time- and resource-intensive and not suitable for rapid analysis of a large number of samples, due to lack of a high-throughput solution for analysis of FTHFS gene amplicons (Leaphart and Lovell, 2001; Xu et al., 2009; Gagen et al., 2010). Lack of a dedicated information resource/database for acetogen-specific homology and taxonomic comparisons has also restricted the development of high-throughput methods (Lovell and Hui, 1991; Xu et al., 2009; Gagen et al., 2010; Planý et al., 2019). Therefore, we recently published AcetoBase, an information resource for FTHFS data, which can assist in high-throughput analysis of acetogenic community diversity and taxonomic assignments of sequence reads from high-throughput sequencing platforms (Singh et al., 2019).

The goal of this study was to present a proof-of-principle for high-throughput sequencing of FTHFS gene amplicons. More specifically the aim was to set up and validate our bioinformatics pipeline “AcetoScan” for unsupervised data analysis of the raw high-throughput sequence data using our previously developed resource “AcetoBase” (Singh et al., 2019). For the analyses, samples from a biogas digester were selected. Biogas/anaerobic digester environments are well-studied in terms of overall bacterial community composition as well as dynamics and acetogens. Previous studies with the bioreactors used in the present study have also confirmed a diverse acetogenic community, making it suitable for the evaluation (Müller et al., 2013; Westerholm et al., 2015; Müller et al., 2016).

## Materials and Methods

### Sample Collection and DNA Extraction

Samples were taken on five different time-points (date: 150303, 150414, 150519, 150709, and 151117) from a continuous stirred-tank biogas reactor (GR2) in the Anaerobic Microbiology and Biotechnology Laboratory, Swedish University of Agricultural Sciences, Uppsala. The reactor was operated with mixed food waste at 37°C, an organic loading rate of 2.5 ± 0.42 g VS L^–1^ day^–1^ and NH_4_^+^-N 5.4 g/L, while other operating parameters were as described for reactor D^TE^37 by Westerholm et al. (2015). Genomic DNA extraction was performed with the FastDNA™ Spin kit for soil (MP Biomedicals, 2015) with an additional wash step with 500 μL 5.5 M Guanidine thiocyanate (Sigma-Aldrich, 2020) for humic acid removal (Singh, 2020). Reactor and DNA samples were stored at −20°C until further processing.

### PCR Amplification, Library Preparation and Sequencing

Partial FTHFS gene amplicons were generated by the primer pairs and PCR protocol developed by Müller et al. (2013) with the modifications FTHFS_fwd (5′-CCIACICCISYIGGNGARGGNAA-3′) and FTHFS_rev (5′-ATITTIGCIAAIGGNCCNSCNTG-3′). FTHFS amplicons were purified by E-Gel^®^ iBase™ Power System (Invitrogen, 2012) and E-Gel^®^ EX with SYBR^®^ Gold II, 2% SizeSelect pre-cast agarose gels (Invitrogen, 2014). Sequencing libraries were prepared from 20 ng purified FTHFS amplicons using the ThruPLEX DNA-seq Prep Kit (Takara Bio USA, 2017) according to the manufacturer’s protocol. Library preparation and sequencing were performed by the SNP&SEQ Technology Platform at the National Genomics Infrastructure (NGI) Sweden and Science for Life Laboratory in Uppsala. Paired-end sequencing was performed on Illumina MiSeq with 300 base pairs (bp) read length using v3 sequencing chemistry (UGC, 2018).

### Development and Implementation of the AcetoScan Pipeline

AcetoScan is a dedicated data analysis pipeline for FTHFS gene sequences. It is a fully automated pipeline for data filtering, taxonomic annotation, phylogenetic tree reconstruction and visualization of sequence data. Input sequence format can be in fasta format or compressed fastq format. AcetoScan is primarily tested with raw sequence data in compressed fastq format from the Illumina MiSeq platform. AcetoScan allows the user to run the analysis with raw sequence data using the command acetoscan based on parameters of the user’s choice (read-type, quality threshold, clustering threshold, minimum cluster size, e-value and phylogenetic tree bootstraps). If the user does not specify any analysis parameters, default parameters are used instead (see AcetoScan user manual). The FTHFS gene amplicon generated with the primers used in the present study has an average length of ∼635 bp. Illumina MiSeq paired-end sequencing can generate the sequence data of only 2 × 300 bp (600 bp), so after the quality control step, it is practically impossible to merge the paired-end reads. Therefore, AcetoScan is at present optimized for data analysis of only one type of sequencing reads at a time, either forward reads (R1) or reverse reads (R2).

AcetoScan carries out data analysis in four major steps (Table 1). In the first step, raw sequences are subjected to primer sequence trimming and quality filtering. The second step comprises dereplication, denoising, chimera filtering, operational taxonomic units (OTU) cluster generation, filtering of non-target sequences, best-frame analysis and taxonomic assignments. In this step, AcetoScan uses the AcetoBase reference protein dataset for selection of the target sequences and taxonomic annotations. In the third step, multiple sequence alignment of the OTU sequences is performed with the subsequent generation of a phylogenetic tree. The last step is data visualization, in which all the results generated in steps two and three are rendered in different plots and interactive graphs.

**TABLE 1:** Overview of the AcetoScan analysis process.

Step	Process	Task	Dependency
1	Quality control	Primer sequence trimming and quality filtering	Cutadapt (Martin, 2017)
2	Sequence data analysis	Dereplication, denoising, chimera removal, clustering, OTU picking	VSEARCH (Rognes et al., 2016)
		Filtering non-target sequences and best frame analysis	Bioperl (Stajich et al., 2002), NCBI-blast+ (Camacho et al., 2009), AcetoBase (Singh et al., 2019)
		OTU table generation and taxonomic assignment	NCBI-blast+, VSEARCH, AcetoBase
3	Phylogenetic inference	Multiple sequence alignment	MAFFT (Katoh and Standley, 2013)
		Phylogenetic tree computation	Fasttree (Price et al., 2009)
4	Visualization	Plotting various plots for taxonomic abundance, alpha and beta diversity graphs, phylogenetic tree visualization	R and dependencies (R Core Team, 2011)

The AcetoScan pipeline can also perform data analysis on the FTHFS gene sequences in fasta sequences format. In addition to the command acetoscan, there are three commands which can be used to process the FTHFS sequence data. The command acetocheck takes fasta format sequences as input, filters out any non-FTHFS sequences and performs reading frame analysis to select the longest reading frame without internal stop codons. If the user wants to taxonomically annotate the FTHFS fasta sequences, the command acetotax can be used, which is acetocheck followed by taxonomic annotation of the filtered sequences. If a user wants to prepare a phylogenetic tree, the command acetotree can be used, which is acetotax followed by phylogenetic tree generation. These commands are implemented for better utilization of time and computational resources when analyzing large fasta sequence datasets. AcetoScan pipeline can also be used as a Docker container for operating system independent and software installation free data analysis.

### *In silico* Mock Community Construction

To evaluate the AcetoScan pipeline for different analysis parameters, *in silico* mock communities were constructed. Three datasets were generated to target different taxonomic levels, i.e., phylum, family and genus. For the construction of the phylum-level mock community, the 11 most abundant phyla in AcetoBase were identified and full-length nucleotide sequences were retrieved. For the family level mock community, full-length nucleotide sequences were collected from the 29 most abundant families in AcetoBase. Finally, for the genus-level mock community, full-length nucleotide sequences from 40 known acetogens (Supplementary Table S1, Singh et al., 2019) were collected from AcetoBase. To assess the effect of different sequence lengths, datasets of ∼635 nucleotides (nt), 300 nt and 150 nt were generated.

For all individual mock communities, the process described in the following paragraph was adopted:

(1) Creation of dataset full-length.
(2) Dataset trimmed was created by aligning the sequences to the sequence amplified by the primer pair from Müller et al. (2013) and the full-length sequence was trimmed to the aligned length of ∼635 nt.
(3) Dataset 300 was created by trimming the dataset trimmed to a sequence length of 300 nt (equivalent to maximum read length from Illumina MiSeq).
(4) Dataset 150 was created by trimming the dataset trimmed to a sequence length of 150 nt.
(5) All the fasta sequences in individual datasets were converted to fastq sequences using the program fastA2Q (Singh, 2019) and compressed in Samplename_L001_R1_001.fastq.gz format.
(6) Individual datasets were processed with the command acetoscan in the AcetoScan pipeline with cluster thresholds of 80% (default) and 100%.
(7) To check the reproducibility, analyses were repeated three times (data not shown).

### FTHFS Sequence Data Analysis With the AcetoScan Pipeline

The raw FTHFS gene amplicon sequence data retrieved from biogas reactor samples in compressed fastq format were used as input. The analysis was carried out separately for the forward (R1) and reverse (R2) reads using the command acetoscan with default parameters: max_length = 300, min_length = 120, Phred quality score = 20, clustering threshold = 80%, minimum cluster size = 2 and evalue 1e-3. The analysis was also carried out for both reads separately, using a clustering threshold of 100%. All analyses presented in this study were performed on a Debian-based Linux operating system running kernel 5.3.4-40-generic in x86-64 architecture (Computer 1, Table 2). To evaluate the computational performance of the AcetoScan pipeline, the analysis was performed on three computers with different specifications (Table 2).

**TABLE 2:** Specification of computers 1–3 and time required for analysis for the forward and reverse reads data generated in this study.

Attributes	Computer 1	Computer 2	Computer 3
Operating system	Debian Linux	Debian Linux	Mac OS Catalina
Architecture	x86_64	x86_64	x86_64
CPU	8	4	4
Model	Intel Core i7-6700	Intel Core i3-4010U	Intel Core i5-5257U
Frequency	3.4 GHz	1.7 GHz	2.7 GHz
Physical RAM (Gb)	40	4	8
Available RAM (Gb)	29	3	4
Clustering threshold (%)	100	100	100
Forward fastq reads	6579804	6579804	6579804
Forward time	35 min	1 h 27 min	1 h 28 min
Reverse fastq reads	6579804	6579804	6579804
Reverse time	38 min	2 hr 27 min	1 hr 30 min

## Results

### Mock-Community Data Analysis

#### Phylum-Level Mock Community Analysis

The mock community analysis at the phylum level for the dataset full-length resulted in the taxonomic assignment of OTUs with >96% accuracy at the 80% clustering threshold, while for the 100% clustering threshold the taxonomic assignment of OTUs was >99% accurate. For the 80% clustering threshold, only one phylum showed misidentification (3.4% of OTUs belonging to phylum Actinobacteria were misidentified as phylum Firmicutes). However, this misidentification was only 0.6% when the clustering threshold was set to 100%. The taxonomic assignments for the dataset trimmed was >99% accurate at the 80% and 100% clustering thresholds. Taxonomic assignment for the dataset 300 was >99% accurate at the 100% clustering threshold. However, when using the 80% clustering threshold, the taxonomic annotations were >95% accurate and only 6.25% of OTUs belonging to the phylum Actinobacteria were assigned wrongly to the phylum Synergistetes. For the dataset 150, taxonomic assignments at both clustering thresholds were >95% accurate except for the phylum Spirochaetes where ∼21% OTUs were misassigned to phylum Bacteroidetes (Supplementary Data 1).

#### Family-Level Mock Community Analysis

For the family level, the dataset full-length analyzed with clustering thresholds 80% and 100% resulted in the identification and taxonomic assignment of OTUs with accuracy > 99%. In this analysis, particularly for the family Clostridiaceae, <5% of OTUs were misidentified as family Peptostreptococcaceae. Similar observations were made for the dataset trimmed at both clustering thresholds. Analysis for the dataset 300 at both clustering thresholds resulted in the similar taxonomic classification of OTUs as obtained for the dataset trimmed, except that 25% of OTUs were affiliated to the family Verrucomicrobiaceae. These could not be annotated with a valid family and were thus denoted “NA.” The affiliation of this “NA” was traced back to class and order level as unclassified Verrucomicrobia. Analysis of the dataset 150 at clustering threshold 80 and 100% resulted in invalid taxonomic assignments, ranging from 5–24% in six out of 29 families (Supplementary Data 2).

#### Acetogen/Genus-Level Mock Community Analysis

When analyzing the dataset full-length from known acetogens, we observed that OTUs resulting from a clustering threshold of 80% were annotated to either the correct species or closest relative in the same genus. However, at a clustering threshold of 100%, all except three samples were accurately annotated. Of these, *Butyribacterium methylotrophicum* was correctly annotated as order Clostridiales, while *Terrisporobacter glycolicus* was correctly annotated as an unknown genus of the family Peptostreptococcaceae, but no further classification of these acetogens was observed. For the dataset trimmed, clustering at the 80% threshold gave accurate identification of OTUs up to genus or species level for all except *Blautia hydrogenotrophica, Terrisporobacter glycolicus*, and *Butyribacterium methylotrophicum*, which were annotated as *Marvinbryantia formatexigens*, *Clostridioides difficile*, and *Eubacterium callanderi*, respectively. At clustering with the 100% threshold, similar observations were made for *B. methylotrophicum* and *T. glycolicus* as for the dataset full-length. For the dataset 300 at both clustering thresholds, similar taxonomic identification was observed as for the dataset trimmed, in which accurate classification was obtained at the family or genus level for the 80% clustering threshold and at the genus or species level for the 100% clustering threshold. For the dataset 150, the 80% clustering threshold resulted in assigning accurate taxonomy at the genus level for most acetogens. However, the species *Clostridium carboxidivorans*, *Clostridium formicaceticum*, *Clostridium magnum*, and *Clostridium scatologenes* were assigned to the genus *Clostridioides.* In addition, *Treponema primitia* and *Caloramator fervidus* were assigned to the genus *Marvinbryantia* and the genus *Alkaliphilus*, respectively, and *Acetobacterium woodii* and *Butyribacterium methylotrophicum* were assigned to *Eubacterium*. At the 100% clustering threshold, five out of 40 samples showed misidentification at the genus level. *Acetobacterium woodii* was affiliated to *Romboutsia*, *Blautia hydrogenotrophica* to *Robinsoniella*, *Caloramator fervidus* to the genus *Alkaliphilus* and *Clostridium formiacaceticum* to *Anaerovirgula*. *Acetoanaerobium noterae* was annotated as *[Clostridium] sticklandii* (Supplementary Data 3).

### FTHFS Gene Amplicon Sequencing

Ultra-deep sequencing of five multiplexed samples on the Illumina MiSeq platform resulted in a sequence data size of 8.2 Gigabytes (compressed size 4.68 Gigabytes). A total of 6.5 million read pairs and, on average, 1.32 million read-pairs per sample were generated. The number of reads generated per sample and the number of reads per sample after quality filtering is presented in Supplementary Table T1.

### FTHFS Gene Sequence Data Analysis

#### Analysis of Forward Reads

**FIGURE 1:**
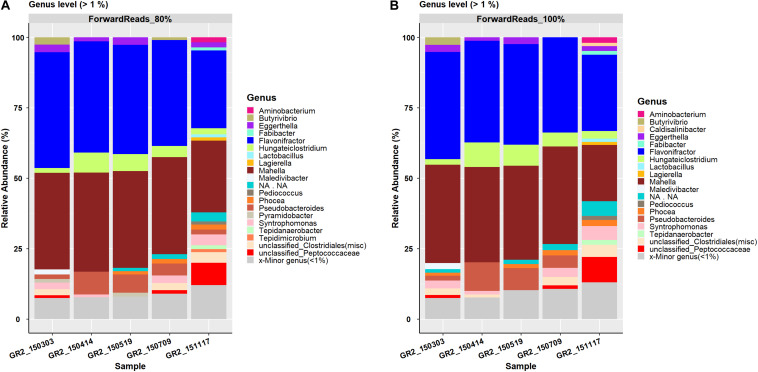
Forward reads processing results representation of the genus-level bar plot with relative abundance > 1% for data processed at a clustering threshold of **(A)** 80% and **(B)** 100%. Genera with relative abundance < 1% are merged in the category ‘Minor genera’.

Forward reads data were analyzed with the command acetoscan with clustering thresholds of 80 and 100%. This generated in total 577 OTUs belonging to 13 phyla and 168 genera, and 1171 OTUs belonging to 13 phyla and 176 genera, respectively (Table 3). The community composition at the family level [relative abundance (RA) > 1%] for both clustering thresholds was similar, except for the *Tepidimicrobium*.NA (RA 1.06%), which was observed in sample GR2-151117 at the 80%, but not the 100%, clustering threshold. At the genus level, 20 genera with relative abundance > 1% were seen for the 80% clustering threshold, while 19 genera with relative abundance > 1% were seen at the 100% clustering threshold%. At the 80% clustering threshold, *Pyramidobacter* was observed in samples GR2-150303 (RA 1.3%) and GR2-150519 (RA 1.35%) and *Tepidimicrobium* in sample GR2-151117 (1.05%) (Figure 1A). However, these two genera were not seen (RA > 1%) at the 100% clustering threshold, where instead *Caldisalinibacter* was observed in one sample, GR2-151117 (1.19%) (Figure 1B). At the species level (RA > 5%), community composition was similar to that at the genus level, with only five major species observed at both clustering thresholds. The RA of these species differed marginally between the clustering thresholds (Supplementary Data 4, 5).

**TABLE 3:** Details of the dataset submitted to AcetoBase, with number of OTUs and their AcetoBase accession number range.

Read type	Clustering threshold (%)	Number of OTUs	AcetoBase Accession number
Forward/R1	80	577	UN_0000020117:UN_0000020693
Forward/R1	100	1171	UN_0000020694:UN_0000021864
Reverse/R2	80	395	UN_0000021865:UN_0000022259
Reverse/R2	100	1241	UN_0000022260:UN_0000023500

#### Analysis of Reverse Reads

**FIGURE 2:**
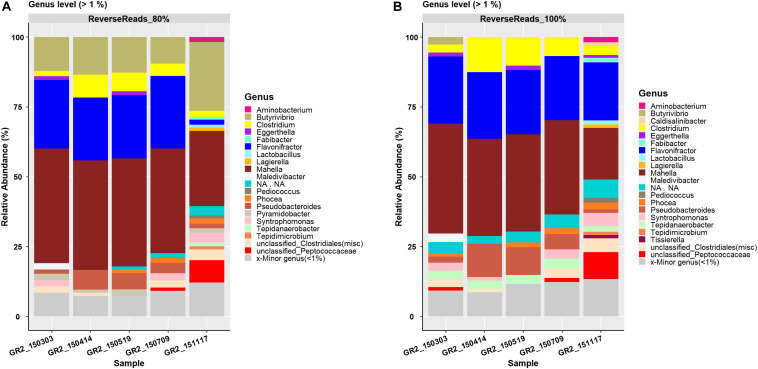
Reverse reads processing result representation of the genus-level bar plot with relative abundance > 1% for the data processed at a clustering threshold of **(A)** 80% and **(B)** 100%. Genera with relative abundance < 1% are merged in the category ‘Minor genera’.

A total of 395 and 1241 OTUs were generated by processing the reverse reads data at a clustering threshold of 80 and 100%, respectively (Table 3). These OTUs were affiliated to 12 phyla and 137 genera (80%) and 12 phyla and 148 genera (100%), respectively. For both clustering thresholds, the same 17 families were observed. At the genus level, 20 and 21 genera were reported for clustering threshold 80 and 100%, respectively, with *Pyramidobacter* (RA 0–2.4%) only seen at the 80% threshold and *Caldisalinibacter* (RA 0–1.2%) and *Tissierella* (RA 0–1.2%) only seen at the 100% threshold (Figures 2A,B). At the species level, five major species, i.e., *Clostridium beijerinckii*, *Flavonifractor* sp., *Mahella australiensis*, an unknown *Peptococcaceae* bacterium and *Pseudobacteroides cellulosolvens*, were observed to have RA > 5% at the 80% clustering threshold, with an additional occurrence of *Butyrivibrio proteoclasticus* (RA 9–25%), which was not seen to have RA > 5%, at the 100% clustering threshold. The five major species were seen at both the 80% and 100% clustering thresholds (Supplementary Data 6, 7).

#### Comparison of Forward and Reverse Reads Processing Results

**FIGURE 3:**
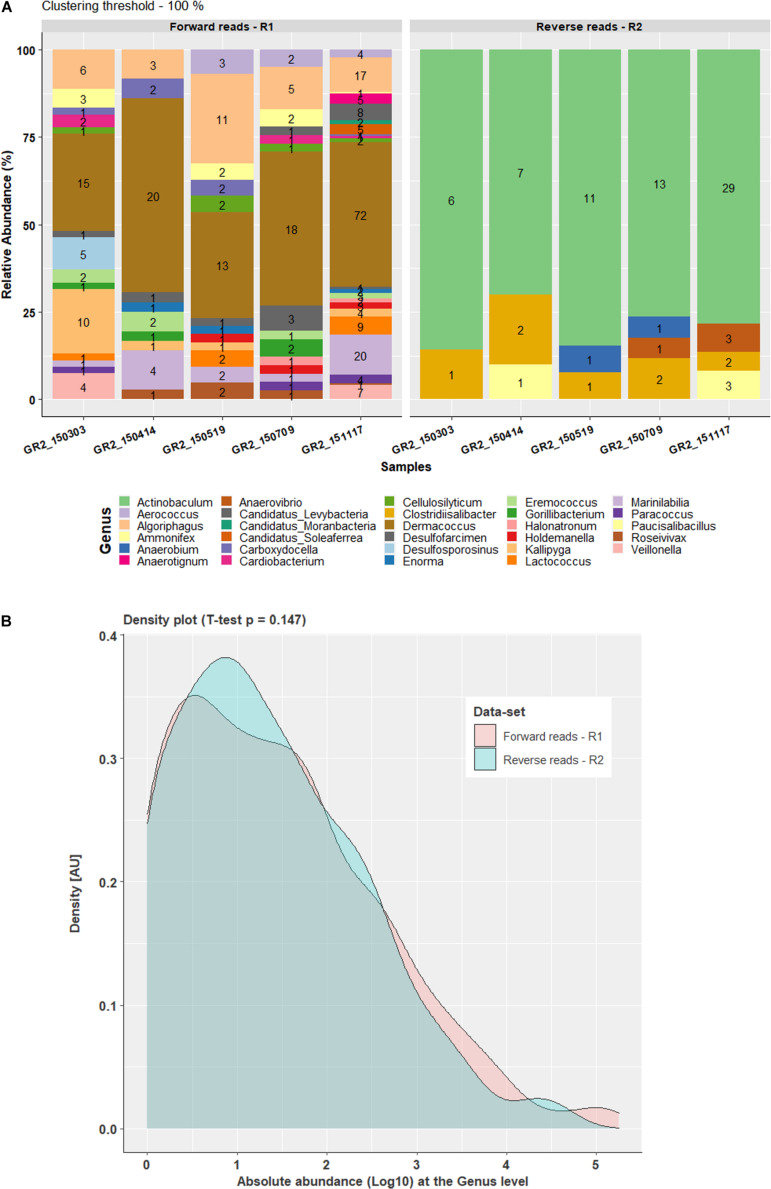
Comparison of forward and reverse reads data-set at genus level with 100% clustering threshold. **(A)** Bar plot representing the percentage difference in the relative abundance of the unique OTUs for the forward and reverse taxonomy at the genus level, where values on the bars indicate the count of the respective OTU in the abundance table. **(B)** Density plot for comparison of log_10_-transformed complete datasets of the forward and reverse read absolute abundance at the genus level.

Comparison of data processed at the 100% clustering threshold at the family level for both forward reads and reverse reads showed a very similar pattern for the top five families Clostridiaceae, Hungateiclostridiaceae, Ruminococcaceae, Syntrophomonadaceae and Thermoanaerobacterales family_IV Incertae Sedis. At the genus level, comparison of the forward and reverse reads showed a similar pattern for the four most abundant genera *Flavonifractor*, *Pseudobacteroides*, *Syntrophomonas*, and *Mahella*. However, genus *Hungateiclostridium* (RA 2–9%) was only recovered in forward reads results (Figures 1B, 2B) and *Clostridium* was only observed in reverse reads results (RA 2.9–13%). At the genus level, 24 genera were unique and observed only in forward read result while five genera (*Actinobaculum*, *Anaerobium*, *Anaerovibrio*, *Clostridiisalibacter*, *Paucisalibacillus*) appeared uniquely in reverse read result (Figure 3A). Even though some differences were seen between forward and reverse reads, a density plot comparing the absolute abundance of the whole dataset from forward and reverse reads at the genus level did not show significant differences in Student’s *t*-test (*p* = 0.147) (Figure 3B). Moreover, an additional independent 2-group Mann–Whitney *U* Test/Wilcox test (*p* = 0.363) and Kruskal–Wallis Test (*p* = 0.363) did not show any significant difference in the absolute abundance of forward and reverse read dataset at the genus level.

#### Computational Competence Comparison

The sequence data generated were used to compare the computation performance of the AcetoScan pipeline. Three computers with different specifications were used to process the raw sequence data, and the times required for the analysis were compared. The analysis took the shortest time (35 and 38 min) on computer 1 because of comparatively better hardware (see Table 2). The data analysis performed on computers 2 and 3 was generally similar for both forward and reverse reads data (∼1 h 28 min), except for reverse reads data processed on computer 2 (∼2 h 27 min). Complete computer specifications are provided in Table 2.

## Discussion

### High-Throughput Sequencing of FTHFS Amplicons

To our knowledge, this is the first study to succeed in producing and analyzing high-throughput sequencing data from FTHFS gene amplicons with multiplexed samples. A previous attempt at multiplexed sequencing of the FTHFS gene has been reported (Planý et al., 2019), but the reliability and reproducibility of that analysis cannot be tested as no data were submitted to public repositories/databases. Most previous studies dealing with the FTHFS gene were based on clone library sequencing or terminal restriction fragment length polymorphism (T-RFLP) analysis (e.g., Pester and Brune, 2006; Hori et al., 2011; Westerholm et al., 2011). Both methods are used widely for microbial community analysis and are considered reliable. However, both are also low-throughput and time- and resource-intensive and have many shortcomings. For example, clone library sequencing can give long sequences of good quality but can be biased by ligation, transformation and colony selection. The T-RFLP method can be useful for community dynamics profiling of a large number of samples, but the resolution of the community may be limited, the reliability of restriction fragments can be questioned and there is no taxonomic information associated with the restriction fragments (Avis et al., 2006; Prakash et al., 2014; De Vrieze et al., 2018).

For the high-throughput sequencing on Illumina platforms, forward and reverse reads are generally merged during data-analysis. To merge the read-pairs, the amplicon size must be less than twice the read length generated by respective Illumina platform and if paired-end reads cannot be merged, single-end reads can be used for the taxonomic annotations (Menzel et al., 2016; Liu et al., 2020). In case of FTHFS gene, there has been a lack of primer pair which can (1) target of the high diversity in the whole microbial community and (2) generate amplicons ≤ 590 bp. Until now, several primer pairs have been published but they all are limited in their ability to target the overall acetogenic community (Müller et al., 2013). Among those, primers developed by Müller et al. (2013) covered more diverse FTHFS sequences as compared to other published FTHFS primer pairs. Thus, although these primers generate amplicons of ∼635 bp and therefore read pairs produced from these amplicons on Illumina MiSeq platform cannot be merged, we used the primers from Müller et al. (2013) for our high-throughput sequencing approach. The effect of un-paired forward and reverse reads on resulting community profile and effect of different read lengths on reliability of taxonomic classification is further reviewed in the discussion.

The high-throughput sequencing of partial FTHFS gene amplicons in this study generated an average of 1.32 M fastq reads (pairs) per sample on Illumina MiSeq. For meta-barcoding studies, a sequencing depth of approximately 15,000 reads is sufficient to enumerate the community structure and diversity of taxa that are not considered rare (Kuczynski et al., 2010; Caporaso et al., 2012; Bukin et al., 2019). Thus, the sequence data generated in our study can be considered sufficient to illustrate accurate and statistically legitimate community composition across samples up to the genus level. With the method, it is possible to sequence large numbers of samples using different strategies, e.g., deep sequencing of fewer samples to find unknown members of the acetogenic community or sufficiently deep sequencing of a larger number of samples from different environments or time-series data to follow and illustrate community changes.

High-throughput sequencing data require a reproducible and accurate data analysis pipeline and a curated database for taxonomic classification. Specific bioinformatics skills are also required, together with computational resources for handling and processing the sequencing data. These skills and resources are not always easily accessible in a cross- or multi-disciplinary research environment and rarely outside the research arena in practical field applications. For this reason, we developed a new high-throughput automated analysis method/pipeline for unsupervised estimation of the acetogenic community.

### Testing AcetoScan Pipeline With *in silico* Mock Community

To test the reproducibility, reliability and accuracy of AcetoScan, mock communities at phylum, family and genus level were constructed and analyzed using different clustering thresholds (80 and 100%) and different sequence lengths (full-length, trimmed, 300 and 150). Repeated analysis of the respective datasets showed reproducible (duplicate) results, where taxonomic assignments of mock communities reliably corresponded with the clustering thresholds and sequence lengths (data not shown for duplicate results). At family and genus level, the taxonomic affiliations were accurate at the 100% clustering threshold for the datasets full-length, trimmed and 300 nt. However, when the sequence clustering threshold was reduced to 80%, only the family level could be reliably classified. The sequence length of 150 nt could not be accurately and reliably used for rare taxa, but still illustrated the overall community dynamics of more abundant taxa and at higher taxonomic levels, i.e., phylum, class, and order.

The differences in community structure at the different clustering thresholds could easily be explained by the fact that any marker gene has its specific percentage clustering thresholds which are required for accurate classification at different taxonomic levels (Qin et al., 2014; Yang et al., 2014). Length of FTHFS sequence, the origin of sequence (level of sequence similarity or variation among different taxa) and type of sequence similarity searches can also influence taxonomic classification at different clustering threshold levels. A percentage similarity threshold of 78% (class Clostridia) in translated nucleotide versus protein searches has been shown to be sufficient for taxonomic classification at the genus level of FTHFS sequences (Singh et al., 2019), and thus the 80% clustering threshold is used as the default cut-off value in the AcetoScan pipeline. The full-length and trimmed datasets helped understand the accuracy of classification, while the 300 and 150 datasets indicated the classification accuracy when using different sequencing platforms, i.e., PacBio, Oxford Nanopore, Illumina MiSeq, HiSeq, NextSeq, or Novaseq series machines, which produce different read lengths.

Blast best-hit ambiguity and re-classification of taxa might also lead to incomplete or false classification, as observed for the acetogen mock community. At the 100% clustering threshold, *Terrisporobacter glycolicus*, a member of the family Peptostreptococcaceae, was assigned correctly at the family level but with no further classification, despite *T. glycolicus* being present in the AcetoBase reference dataset. This might be due to the blast best-hit ambiguity. Furthermore, [*Butyribacterium*] *methylotrophicum*, also present in AcetoBase, was assigned at order level as Clostridiales, probably because genus [*Butyribacterium*] has not yet been classified and named as a valid genus. [*Clostridium*] *sticklandii* has been renamed *Acetoanaerobium sticklandii*, but its homotypic synonym is still in use, and might instead be taxonomically assigned as *Acetoanaerobium noterae.*

### Analysis of High-Throughput Sequencing Data With AcetoScan

Processing FTHFS forward and reverse reads with AcetoScan resulted in similar community dynamics for both at clustering thresholds of 80% and also 100%. Aberrations in the taxonomic profiling, with certain taxa being present in one dataset and not in another (Figures 1B, 2B), have two possible explanations: (i) Low-abundance taxa identified in one dataset might be below the visualization threshold in the other dataset, and thus be merged. This is supported by the fact that the genera differing between forward and reverse reads had <1% relative abundance. There were no statistically significant (Student *t*-test *p*-value 0.147) differences between the community profile generated from the forward and reverse reads if absolute abundance at the genus level is compared, and thus the differences were negligible. (ii) Lack of read pair merging, resulting in individual processing of the respective read type leading to a slight deviation in the read count matrix. This could result in slight variations in the community structure presented in the plots for the respective sequence read types, although such variations might not be statistically significant in whole datasets. The AcetoScan pipeline can process long fastq sequences and does not require read-pairs. It can, therefore, be used for the data generated on sequencing platforms other than Illumina MiSeq or fasta sequences generated by traditional clone libraries using the Sanger sequencing method. The AcetoScan pipeline is available for Debian Linux and MacOS operating systems as well as Docker container, and can perform the analysis even on a laptop computer with standard hardware specifications. Since a high-performance computer is not easily available to all, the ability of AcetoScan to run on a laptop computer is a strong benefit of our bioinformatics analysis pipeline.

### The Vigilance for Acetogens

Acetogenesis is a complex physiological trait and not a phylogenetic/genomic property (e.g., Drake, 1994; Tanner and Woese, 1994; Küsel et al., 2001; Drake et al., 2002; Schuchmann and Müller, 2016; Singh et al., 2019). It should thus be referred to as a flexible functional characteristic, rather than a rigid group of few taxonomic ranks. The FTHFS gene is an important enzyme of the WLP and has been used widely to assess the acetogenic population. It can also be present in other groups of microorganisms, such as syntrophic acid-oxidizing bacteria, sulfate-reducing bacteria and archaea. The mere presence of a FTHFS gene does not define a bacterium as an acetogen, as proposed in a recent publication by Kim et al. (2020). There are instances where the terms acetogens, homoacetogens and acetogenesis are still not properly understood and have been misused (Das and Ljungdahl, 2000; Küsel and Drake, 2011; Müller and Frerichs, 2013; Singh et al., 2019). The FTHFS OTUs generated in the AcetoScan pipeline do not claim that the detected OTUs is an ‘acetogen,’ and we propose a three-step procedure which must be followed before an OTU can be defined as an acetogen: (1) Identification of the bacterial candidate with the FTHFS gene; (2) identification of the WLP genes in the genome of the candidate; and (3) experimental validation of acetogenic metabolism in an environment which sustains acetogenesis. Steps 1 and 2 can be conducted using AcetoScan and AcetoBase. To avoid misuse of the terms acetogen, acetogenic community or acetogenesis, we suggest that organism should be considered as a candidate which has the potential for acetogenesis if its genome contains the complete WLP or at least its main enzyme-encoding genes, i.e., formyltetrahydrofolate synthetase, acetate kinase and acetyl-CoA synthase/carbon monoxide dehydrogenase complex (Singh et al., 2019).

### Future Perspectives

Acetogens are among the most versatile organisms on the planet. This metabolic versatility lies in their ability to grow on the thermodynamic borderline and produce organic precursor acetyl-CoA by reduction of CO_2_ (Drake and Küsel, 2003; Lever, 2012; Schuchmann and Müller, 2014). Some acetogens are reported to harbor a unique hydrogen-dependent CO_2_ reductase that has the highest biological hydrogen production rates known (Müller, 2019). In industrial processes, acetogens are used as microbial cell factories to trap CO_2_ and production of biofuels from syngas (Liew et al., 2016). In recent years, acetogenic bacteria have also been the focus of studies on microbial fuel cells, where electricity is generated from microorganism-powered batteries, and on microbial electro-synthesis systems, where surplus renewable electric power can be used to synthesize organic compounds (Nevin et al., 2011; Parameswaran et al., 2011; Scott and Yu, 2015; Saheb-Alam et al., 2018). Therefore, acetogens are important microorganisms for the circular bio-economy and for mitigating climate change (Oren, 2012; Liew et al., 2016; Müller, 2019; Wiechmann and Müller, 2019). Acetogens are also abundantly present in the human gut, but their role in human gut physiology and the gut-brain relationship require further investigation (e.g., Gibson et al., 1990; Leclerc et al., 1997; Ohashi et al., 2007; Rey et al., 2010; Laverde Gomez et al., 2019). Moreover, some acetogens have been found to associate with plants in aquatic habitats and can fix atmospheric nitrogen (Küsel et al., 1999; Pester and Brune, 2006; Ohkuma et al., 2015). Therefore, acetogens are ubiquitous and prominent microorganisms in the ecosystem and more focused and extensive studies are needed to decode their interconnection with human and other organisms. Consequently, our method and AcetoScan can be of great importance in the exploration of potential acetogenic communities in different and natural environments.

## Conclusion

A novel pipeline, AcetoScan, was validated with several sets of *in silico* mock communities and successfully used for the analysis of the acetogenic community in multiplexed biogas reactor samples. AcetoScan is designed for rapid and accurate analysis of FTHFS amplicon sequence data and taxonomic annotation using AcetoBase with minimum or no user supervision and results in interactive and publication-ready graphs and plots from raw sequence data. AcetoScan does not necessarily require a high-performance cluster computer and analysis can be performed on a standard computer, with analysis time varying depending on the computer configuration. Our sequencing approach and AcetoScan analysis pipeline can become the method of choice for research on natural or constructed environments where the acetogenic community and its dynamics are important.

## Data Availability Statement

The FTHFS OTU sequences generated from analysis of high-throughput sequencing data have been submitted to AcetoBase. Accession numbers of the respective OTU dataset are presented in Table 3. The raw data generated by Illumina Miseq have been submitted to NCBI SRA (study: SRP257947, run IDs: SRR11590656:SRR115 90660) with BioProject accession number PRJNA627452 (https://www.ncbi.nlm.nih.gov/bioproject/PRJNA627452). The AcetoScan pipeline, with test data, user manual and instruction video, is available on the AcetoScan GitHub repository (http://github.com/abhijeetsingh1704/AcetoScan). The Docker image of AcetoScan pipeline can be accessed on the Docker hub (https://hub.docker.com/r/abhijeetsingh1704/acetoscan).

## Author Contributions

ASi, ASc, and BM conceived the idea for the study. ASc acquired funding for the project. ASi performed the experiment and data analysis and developed the AcetoScan pipeline, together with JN and in discussion with ASc and BM. EB-R critically assessed the set-up of the AcetoScan pipeline. ASi wrote the manuscript with valuable help from all co-authors. All the authors contributed to the article and approved the submitted version.

## Conflict of Interest

The authors declare that the research was conducted in the absence of any commercial or financial relationships that could be construed as a potential conflict of interest.

## Abbreviations

bp: base pairs
FTHFS: formyltetrahydrofolate synthetase
nt: nucleotides
OTU: operational taxonomic units
WLP: Wood–Ljungdahl pathway.

## References

[B1] Avis P. G. Dickie I. A. Mueller G. M. (2006). A “dirty” business: Testing the limitations of terminal restriction fragment length polymorphism (TRFLP) analysis of soil fungi. *Mol. Ecol.* 15 873–882. 10.1111/j.1365-294X.2005.02842.x 16499709

[B2] Breznak J. A. Kane M. D. (1990). Microbial H2/CO2 acetogenesis in animal guts: nature and nutritional significance. *FEMS Microbiol. Lett.* 7 309–313. 10.1016/0378-1097(90)90471-2 2128799

[B3] Bukin Y. S. Galachyants Y. P. Morozov I. V. Bukin S. V. Zakharenko A. S. Zemskaya T. I. (2019). The effect of 16s rRNA region choice on bacterial community metabarcoding results. *Sci. Data* 6:190007. 10.1038/sdata.2019.7 30720800 PMC6362892

[B4] Camacho C. Coulouris G. Avagyan V. Ma N. Papadopoulos J. Bealer K. (2009). BLAST+: Architecture and applications. *BMC Bioinformatics* 10:421. 10.1186/1471-2105-10-421 20003500 PMC2803857

[B5] Caporaso J. G. Lauber C. L. Walters W. A. Berg-Lyons D. Huntley J. Fierer N. (2012). Ultra-high-throughput microbial community analysis on the Illumina HiSeq and MiSeq platforms. *ISME J.* 6 1621–1624. 10.1038/ismej.2012.8 22402401 PMC3400413

[B6] Dar S. A. Kleerebezem R. Stams A. J. M. Kuenen J. G. Muyzer G. (2008). Competition and coexistence of sulfate-reducing bacteria, acetogens and methanogens in a lab-scale anaerobic bioreactor as affected by changing substrate to sulfate ratio. *Appl. Microbiol. Biotechnol.* 78 1045–1055. 10.1007/s00253-008-1391-8 18305937 PMC2271084

[B7] Das A. Ljungdahl L. G. (2000). “Acetogenesis and Acetogenic Bacteria,” in *Encyclopedia of Microbiology, Four-*, Vol. Set ed. Joshua L. (London: Academic Press), 18–27.

[B8] Das A. Ljungdahl L. G. (2003). “Electron-Transport System in Acetogens,” in *Biochemistry and Physiology of Anaerobic Bacteria*, eds Ljungdahl L. G. Adams M. W. M. W. Barton L. L. Ferry J. G. Johnson M. K. (New York, NY: Springer), 191–204. 10.1007/0-387-22731-8_14

[B9] De Vrieze J. Ijaz U. Z. Saunders A. M. Theuerl S. (2018). Terminal restriction fragment length polymorphism is an “old school” reliable technique for swift microbial community screening in anaerobic digestion. *Sci. Rep.* 8:16818. 10.1038/s41598-018-34921-7 30429514 PMC6235954

[B10] Drake H. L. (1994). *Acetogenesis.* Boston, MA: Springer.

[B11] Drake H. L. Daniel S. L. Küsel K. Matthies C. Kuhner C. Braus-Stromeyer S. (1997). Acetogenic bacteria: What are the in situ consequences of their diverse metabolic versatilities. *BioFactors* 6 13–24. 10.1002/biof.5520060103 9233536

[B12] Drake H. L. Gößner A. S. Daniel S. L. (2008). Old Acetogens, New Light. *Ann. N. Y. Acad. Sci.* 1125 100–128. 10.1196/annals.1419.016 18378590

[B13] Drake H. L. Küsel K. (2003). “How the Diverse Physiologic Potentials of Acetogens Determine Their In Situ Realities,” in *Biochemistry and Physiology of Anaerobic Bacteria*, eds Ljungdahl J. M. K. Adams M. W. Barton L. L. Ferry J. G. (New York, NY: Springer), 171–190. 10.1007/0-387-22731-8_13

[B14] Drake H. L. Küsel K. Matthies C. (2002). Ecological consequences of the phylogenetic and physiological diversities of acetogens. *Antonie van Leeuwenhoek, Int. J. Gen. Mol. Microbiol.* 81 203–213. 10.1023/A:1020514617738 12448719

[B15] Drake H. L. Küsel K. Matthies C. (2013). “Acetogenic prokaryotes,” in *The Prokaryotes: Prokaryotic Physiology and Biochemistry*, ed. Rosenberg E. (Berlin: Springer), 3–60. 10.1007/978-3-642-30141-4_61

[B16] Fischer M. A. Güllert S. Refai S. Künzel S. Deppenmeier U. Streit W. R. (2019). Long-term investigation of microbial community composition and transcription patterns in a biogas plant undergoing ammonia crisis. *Microb. Biotechnol.* 12 305–323. 10.1111/1751-7915.13313 30381904 PMC6390037

[B17] Fu B. Jin X. Conrad R. Liu H. Liu H. (2019). Competition between chemolithotrophic acetogenesis and hydrogenotrophic methanogenesis for exogenous H2/CO2 in anaerobically digested sludge: impact of temperature. *Front. Microbiol.* 10:2418. 10.3389/fmicb.2019.02418 31749772 PMC6842956

[B18] Gagen E. J. Denman S. E. Padmanabha J. Zadbuke S. Jassim R. Al Morrison M. (2010). Functional gene analysis suggests different acetogen populations in the bovine rumen and tammar wallaby forestomach. *Appl. Environ. Microbiol.* 76 7785–7795. 10.1128/AEM.01679-10 20889794 PMC2988603

[B19] Gibson G. R. Cummings J. H. Macfarlane G. T. Allison C. Segal I. Vorster H. H. (1990). Alternative pathways for hydrogen disposal during fermentation in the human colon. *Gut* 31 679–683. 10.1136/gut.31.6.679 2379871 PMC1378495

[B20] Henderson G. Leahy S. C. Janssen P. H. (2010). Presence of novel, potentially homoacetogenic bacteria in the rumen as determined by analysis of formyltetrahydrofolate synthetase sequences from ruminants. *Appl. Environ. Microbiol.* 76 2058–2066. 10.1128/AEM.02580-09 20118378 PMC2849231

[B21] Hori T. Sasaki D. Haruta S. Shigematsu T. Ueno Y. Ishii M. (2011). Detection of active, potentially acetate-oxidizing syntrophs in an anaerobic digester by flux measurement and formyltetrahydrofolate synthetase (FTHFS) expression profiling. *Microbiology* 157 1980–1989. 10.1099/mic.0.049189-0 21474532

[B22] Hügler M. Sievert S. M. (2011). Beyond the Calvin Cycle: Autotrophic Carbon Fixation in the Ocean. *Ann. Rev. Mar. Sci.* 3 261–289. 10.1146/annurev-marine-120709-142712 21329206

[B23] Invitrogen (2012). *E-Gel^®^ IBase™ Power System and E-Gel^®^ Safe Imager™ Real-Time Transilluminator. Life Technologies; E-Gel User Guide*, Part no. 25-0951, Pub. No. – MAN0000573. Carlsbad, CA: Invitrogen, 1–44.

[B24] Invitrogen (2014). *E-Gel^®^ Technical Guide. Life Technologies; E-Gel Technical Guide*, Pub. No. – MAN0000375, Revision A.0. Carlsbad, CA: Invitrogen, 1–140.

[B25] Katoh K. Standley D. M. (2013). MAFFT multiple sequence alignment software version 7: Improvements in performance and usability. *Mol. Biol. Evol.* 30 772–780. 10.1093/molbev/mst010 23329690 PMC3603318

[B26] Kim S.-H. Mamuad L. L. Islam M. Lee S.-S. (2020). Reductive acetogens isolated from ruminants and their effect on in vitro methane mitigation and milk performance in Holstein cows. *J. Anim. Sci. Technol.* 62 1–13. 10.5187/jast.2020.62.1.1 32082593 PMC7008121

[B27] King G. M. (1991). Measurement of acetate concentrations in marine pore waters by using an enzymatic approach. *Appl. Environ. Microbiol.* 57 3476–3481. 10.1128/AEM.57.12.3476-3481.1991 16348598 PMC183999

[B28] Kotsyurbenko O. R. Nozhevnikova A. N. Soloviova T. I. Zavarzin G. A. (1996). Methanogenesis at low temperatures by microflora of tundra wetland soil. *Antonie van Leeuwenhoek Int. J. Gen. Mol. Microbiol.* 69 75–86. 10.1007/BF00641614 8678482

[B29] Kuczynski J. Liu Z. Lozupone C. McDonald D. Fierer N. Knight R. (2010). Microbial community resemblance methods differ in their ability to detect biologically relevant patterns. *Nat. Methods* 7 813–819. 10.1038/nmeth.1499 20818378 PMC2948603

[B30] Kusel K. Drake H. L. (1994). Acetate synthesis in soil from a bavarian beech forest. *Appl. Environ. Microbiol.* 60 1370–1373. 10.1128/aem.60.4.1370-1373.1994 16349243 PMC201485

[B31] Küsel K. Drake H. L. (2011). “Acetogens,” in *Encyclopedia of Geobiology. Encyclopedia of Earth Sciences Series* Encyclopedia of Earth Sciences Series, eds Reitner J. Thiel V. (Dordrecht: Springer).

[B32] Küsel K. Karnholz A. Trinkwalter T. Devereux R. Acker G. Drake H. L. (2001). Physiological ecology of clostridium glycolicum RD-1, an aerotolerant acetogen isolated from sea grass roots. *Appl. Environ. Microbiol.* 67 4734–4741. 10.1128/AEM.67.10.4734-4741.2001 11571179 PMC93226

[B33] Küsel K. Pinkart H. C. Drake H. L. Devereux R. (1999). Acetogenic and sulfate-reducing bacteria inhabiting the rhizoplane and deep cortex cells of the sea grass Halodule wrightii. *Appl. Environ. Microbiol.* 65 5117–5123. 10.1128/aem.65.11.5117-5123.1999 10543830 PMC91688

[B34] Laverde Gomez J. A. Mukhopadhya I. Duncan S. H. Louis P. Shaw S. Collie-Duguid E. (2019). Formate cross-feeding and cooperative metabolic interactions revealed by transcriptomics in co-cultures of acetogenic and amylolytic human colonic bacteria. *Environ. Microbiol.* 21 259–271. 10.1111/1462-2920.14454 30362296 PMC6378601

[B35] Leaphart A. B. Lovell C. R. (2001). Recovery and Analysis of Formyltetrahydrofolate Synthetase Gene Sequences from Natural Populations of Acetogenic Bacteria. *Appl. Environ. Microbiol.* 67 1392–1395. 10.1128/AEM.67.3.1392 11229939 PMC92742

[B36] Leclerc M. Bernalier A. Donadille G. Lelait M. (1997). H2/CO2 Metabolism in Acetogenic Bacteria Isolated From the Human Colon. *Anaerobe* 3 307–315. 10.1006/anae.1997.0117 16887606

[B37] Lever M. A. (2012). Acetogenesis in the energy-starved deep biosphere-a paradox? *Front. Microbiol.* 2:284. 10.3389/fmicb.2011.00284 22347874 PMC3276360

[B38] Liew F. M. Martin M. E. Tappel R. C. Heijstra B. D. Mihalcea C. Köpke M. (2016). Gas Fermentation-A flexible platform for commercial scale production of low-carbon-fuels and chemicals from waste and renewable feedstocks. *Front. Microbiol.* 7:694. 10.3389/fmicb.2016.00694 27242719 PMC4862988

[B39] Liu T. Chen C. Y. Chen-Deng A. Chen Y. L. Wang J. Y. Hou Y. I. (2020). Joining Illumina paired-end reads for classifying phylogenetic marker sequences. *BMC Bioinformatics* 21:105. 10.1186/s12859-020-3445-6 32171248 PMC7071698

[B40] Lovell C. R. (1994). “Development of DNA Probes for the Detection and Identification of Acetogenic Bacteria,” in *Acetogenesis*, ed. Drake H. L. (Cham: Springer), 236–253. 10.1007/978-1-4615-1777-1_8

[B41] Lovell C. R. Hui Y. (1991). Design and testing of a functional group-specific DNA probe for the study of natural populations of acetogenic bacteria. *Appl. Environ. Microbiol.* 57 2602–2609. 10.1128/aem.57.9.2602-2609.1991 1768134 PMC183627

[B42] Lovell C. R. Leaphart A. B. (2005). Community-level analysis: Key genes of CO2-reductive acetogenesis. *Methods Enzymol.* 397 454–469. 10.1016/S0076-6879(05)97028-6 16260309

[B43] Martin M. (2017). Cutadapt removes adapter sequences from high-throughput sequencing reads. *EMBnet J.* 17 10–12. 10.14806/ej.17.1.200

[B44] Menzel P. Ng K. L. Krogh A. (2016). Fast and sensitive taxonomic classification for metagenomics with Kaiju. *Nat. Commun.* 7 1–9. 10.1038/ncomms11257 27071849 PMC4833860

[B45] Moestedt J. Müller B. Westerholm M. Schnürer A. (2016). Ammonia threshold for inhibition of anaerobic digestion of thin stillage and the importance of organic loading rate. *Microb. Biotechnol.* 9 180–194. 10.1111/1751-7915.12330 26686366 PMC4767286

[B46] MP Biomedicals (2015). *FastDNA™ SPIN Kit for Soil: Instruction Manual, Catalog # 6560-200*, Protocol Revision # 6560-200-07 DEC. Santa Ana, CA: MP Biomedicals.

[B47] Müller B. Sun L. Schnürer A. (2013). First insights into the syntrophic acetate-oxidizing bacteria - a genetic study. *Microbiologyopen* 2 35–53. 10.1002/mbo3.50 23239474 PMC3584212

[B48] Müller B. Sun L. Westerholm M. Schnürer A. (2016). Bacterial community composition and fhs profiles of low- and high-ammonia biogas digesters reveal novel syntrophic acetate-oxidising bacteria. *Biotechnol. Biofuels* 9 1–18. 10.1186/s13068-016-0454-9 26925165 PMC4769498

[B49] Müller V. (2003). Energy Conservation in Acetogenic. *Appl. Environ. Microbiol.* 69 6345–6353. 10.1128/AEM.69.11.6345 14602585 PMC262307

[B50] Müller V. (2019). New Horizons in Acetogenic Conversion of One-Carbon Substrates and Biological Hydrogen Storage. *Trends Biotechnol.* 37 1344–1354. 10.1016/j.tibtech.2019.05.008 31257058

[B51] Müller V. Frerichs J. (2013). *Acetogenic Bacteria.* Hoboken, NJ: John Wiley Sons, Ltd.

[B52] Nevin K. P. Hensley S. A. Franks A. E. Summers Z. M. Ou J. Woodard T. L. (2011). Electrosynthesis of organic compounds from carbon dioxide is catalyzed by a diversity of acetogenic microorganisms. *Appl. Environ. Microbiol.* 77 2882–2886. 10.1128/AEM.02642-10 21378039 PMC3126412

[B53] Ohashi Y. Igarashi T. Kumazawa F. Fujisawa T. (2007). Analysis of Acetogenic Bacteria in Human Feces with Formyltetrahydrofolate Synthetase Sequences. *Biosci. Microflora* 26 37–40. 10.12938/bifidus.26.37

[B54] Ohkuma M. Noda S. Hattori S. Iida T. Yuki M. Starns D. (2015). Acetogenesis from H2 plus CO2 and nitrogen fixation by an endosymbiotic spirochete of a termite-gut cellulolytic protist. *Proc. Natl. Acad. Sci. U. S. A.* 12 10224–10230. 10.1073/pnas.1423979112 25979941 PMC4547241

[B55] Oren A. (2012). There must be an acetogen somewhere. *Front. Microbiol.* 3:22. 10.3389/fmicb.2012.00022 22319520 PMC3269027

[B56] Palacios P. A. Snoeyenbos-West O. Löscher C. R. Thamdrup B. Rotaru A.-E. (2019). Baltic Sea methanogens compete with acetogens for electrons from metallic iron. *ISME J.* 13 3011–3023. 10.1038/s41396-019-0490-0 31444483 PMC6864099

[B57] Parameswaran P. Torres C. I. Lee H. S. Rittmann B. E. Krajmalnik-Brown R. (2011). Hydrogen consumption in microbial electrochemical systems (MXCs): The role of homo-acetogenic bacteria. *Bioresour. Technol.* 102 263–271. 10.1016/j.biortech.2010.03.133 20430615

[B58] Pereló J. G. Velasco A. M. Becerra A. Lazcano A. (1999). Comparative biochemistry of CO2 fixation and the evolution of autotrophy. *Int. Microbiol.* 2 3–10. 10.2436/im.v2i1.9169 10943384

[B59] Pester M. Brune A. (2006). Expression profiles of fhs (FTHFS) genes support the hypothesis that spirochaetes dominate reductive acetogenesis in the hindgut of lower termites. *Environ. Microbiol.* 8 1261–1270. 10.1111/j.1462-2920.2006.01020.x 16817934

[B60] Planý M. Czolderová M. Kraková L. Puškárová A. Buèková M. Šoltys K. (2019). Biogas production: evaluation of the influence of K2FeO4 pretreatment of maple leaves (Acer platanoides) on microbial consortia composition. *Bioprocess Biosyst. Eng.* 42 1151–1163. 10.1007/s00449-019-02112-x 30944995

[B61] Poehlein A. Schmidt S. Kaster A. K. Goenrich M. Vollmers J. Thürmer A. (2012). An ancient pathway combining carbon dioxide fixation with the generation and utilization of a sodium ion gradient for ATP synthesis. *PLoS One* 7:e33439. 10.1371/journal.pone.0033439 22479398 PMC3315566

[B62] Prakash O. Pandey P. K. Kulkarni G. J. Mahale K. N. Shouche Y. S. (2014). Technicalities and Glitches of Terminal Restriction Fragment Length Polymorphism (T-RFLP). *Indian J. Microbiol.* 54 255–261. 10.1007/s12088-014-0461-0 24891731 PMC4039717

[B63] Price M. N. Dehal P. S. Arkin A. P. (2009). Fasttree: Computing large minimum evolution trees with profiles instead of a distance matrix. *Mol. Biol. Evol.* 26 1641–1650. 10.1093/molbev/msp077 19377059 PMC2693737

[B64] Qin Q. L. Xie B. Zhang X. Y. Chen X. L. Zhou B. C. Zhou J. (2014). A proposed genus boundary for the prokaryotes based on genomic insights. *J. Bacteriol.* 196 2210–2215. 10.1128/JB.01688-14 24706738 PMC4054180

[B65] R Core Team (2011). *R: A Language and Environment for Statistical Computing.* Vienna: R Found. Stat. Comput.

[B66] Ragsdale S. W. (2007). Nickel and the carbon cycle. *J Inorg Biochem.* 101 1657–1666. 10.1016/j.jinorgbio.2007.07.014 17716738 PMC2100024

[B67] Ragsdale S. W. Pierce E. (2008). Acetogenesis and the Wood-Ljungdahl pathway of CO2 fixation. *Biochim. Biophys. Acta - Proteins Proteomics* 1784 1873–1898. 10.1016/j.bbapap.2008.08.012 18801467 PMC2646786

[B68] Ragsdale S. W. Wood H. G. (1991). Enzymology of the acetyl-coa pathway of CO2 fixation. *Crit. Rev. Biochem. Mol. Biol.* 26 261–300. 10.3109/10409239109114070 1935170

[B69] Rey F. E. Faith J. J. Bain J. Muehlbauer M. J. Stevens R. D. Newgard C. B. (2010). Dissecting the in vivo metabolic potential of two human gut acetogens. *J. Biol. Chem.* 285 22082–22090. 10.1074/jbc.M110.117713 20444704 PMC2903421

[B70] Rognes T. Flouri T. Nichols B. Quince C. Mahé F. (2016). VSEARCH: a versatile open source tool for metagenomics. *PeerJ* 4:e2584. 10.7717/peerj.2584 27781170 PMC5075697

[B71] Russell M. J. Martin W. (2004). The rocky roots of the acetyl-CoA pathway. *Trends Biochem. Sci.* 29 358–363. 10.1016/j.tibs.2004.05.007 15236743

[B72] Sagheddu V. Patrone V. Miragoli F. Morelli L. (2017). Abundance and diversity of hydrogenotrophic microorganisms in the infant gut before the weaning period assessed by denaturing gradient gel electrophoresis and quantitative PCR. *Front. Nutr.* 4:29. 10.3389/fnut.2017.00029 28695121 PMC5483434

[B73] Saheb-Alam S. Singh A. Hermansson M. Persson F. Schnürer A. Wilén B. M. (2018). Effect of start-up strategies and electrode materials on carbon dioxide reduction on biocathodes. *Appl. Environ. Microbiol.* 84:e02242-17. 10.1128/AEM.02242-17 29222104 PMC5795077

[B74] Sakimoto K. K. Wong A. B. Yang P. (2016). Self-photosensitization of nonphotosynthetic bacteria for solar-to-chemical production. *Science* 351 74–77. 10.1126/science.aad3317 26721997

[B75] Schuchmann K. Müller V. (2014). Autotrophy at the thermodynamic limit of life: A model for energy conservation in acetogenic bacteria. *Nat. Rev. Microbiol.* 12 809–821. 10.1038/nrmicro3365 25383604

[B76] Schuchmann K. Müller V. (2016). Energetics and application of heterotrophy in acetogenic bacteria. *Appl. Environ. Microbiol.* 82 4056–4069. 10.1128/AEM.00882-16 27208103 PMC4959221

[B77] Scott K. Yu E. H. (2015). *Microbial Electrochemical and Fuel Cells: Fundamentals and Applications.* Sawston: Woodhead Publishing.

[B78] Shin J. Song Y. Jeong Y. Cho B. K. (2016). Analysis of the core genome and pan-genome of autotrophic acetogenic bacteria. *Front. Microbiol.* 7:1531. 10.3389/fmicb.2016.01531 27733845 PMC5039349

[B79] Sigma-Aldrich (2020). *Guanidine Thiocyanate. Sigma-Aldrich*, CAS Number 593-84-0, Product-G9277. Darmstadt: Merck KGaA.

[B80] Singh A. (2019). *FastA2Q. Repository: Github.com, Version 1.* 10.13140/RG.2.2.13695.15529 Available online at: https://github.com/abhijeetsingh1704/fastA2Q (accessed May 13, 2020). 28610126

[B81] Singh A. (2020). *Genomic DNA Extraction from Anaerobic Digester Samples.* Protocols.io, protocol ID: 37580. Available online at: 10.17504/protocols.io.bgxkjxkw (accessed May 29, 2020).

[B82] Singh A. Müller B. Fuxelius H. H. Schnürer A. (2019). AcetoBase: a functional gene repository and database for formyltetrahydrofolate synthetase sequences. *Database* 2019:baz142. 10.1093/database/baz142 31832668 PMC6908459

[B83] Stajich J. E. Block D. Boulez K. Brenner S. E. Chervitz S. A. Dagdigian C. (2002). The Bioperl toolkit: Perl modules for the life sciences. *Genome Res.* 12 1611–1618. 10.1101/gr.361602 12368254 PMC187536

[B84] Takara Bio USA Inc (2017). *ThruPLEX^®^ DNA-Seq Kit:* Cat. Nos. R400523, R400428, R400427, R400406 & R400407 (022818). Mountain View, CA: Takara Bio USA Inc 1–28.

[B85] Tanner R. S. Woese C. R. (1994). “A phylogenetic assessment of the acetogens,” in *Acetogenesis*, ed. Drake H.L. (Boston, MA: Springer), 254–269. 10.1007/978-1-4615-1777-1_9

[B86] UGC (2018). *National Genomics Infrastructure (NGI) at Uppsala Genome Center (UGC).* Sweden: UGC.

[B87] Wagner C. Grießhammer A. Drake H. L. (1996). Acetogenic capacities and the anaerobic turnover of carbon in a Kansas prairie soil. *Appl. Environ. Microbiol.* 62 494–500. 10.1128/aem.62.2.494-500.1996 16535237 PMC1388775

[B88] Wang J. Liu H. Fu B. Xu K. Chen J. (2013). Trophic link between syntrophic acetogens and homoacetogens during the anaerobic acidogenic fermentation of sewage sludge. *Biochem. Eng. J.* 70 1–8. 10.1016/j.bej.2012.09.012

[B89] Weiland P. (2010). Biogas production: Current state and perspectives. *Appl. Microbiol. Biotechnol.* 85 849–860. 10.1007/s00253-009-2246-7 19777226

[B90] Westerholm M. Müller B. Arthurson V. Schnürer A. (2011). Changes in the acetogenic population in a mesophilic anaerobic digester in response to increasing ammonia concentration. *Microbes Environ.* 26 347–353. 10.1264/jsme2.ME11123 21869569

[B91] Westerholm M. Müller B. Isaksson S. Schnürer A. (2015). Trace element and temperature effects on microbial communities and links to biogas digester performance at high ammonia levels. *Biotechnol. Biofuels* 8 1–19. 10.1186/s13068-015-0328-6 26396592 PMC4578335

[B92] Westerholm M. Müller B. Singh A. Karlsson Lindsjö O. Schnürer A. (2018). Detection of novel syntrophic acetate-oxidizing bacteria from biogas processes by continuous acetate enrichment approaches. *Microb. Biotechnol.* 11 680–693. 10.1111/1751-7915.13035 29239113 PMC6011928

[B93] Wiechmann A. Müller V. (2019). “Synthesis of Acetyl-CoA from Carbon Dioxide in Acetogenic Bacteria,” in *Biogenesis of Fatty Acids, Lipids and Membranes, Handbook of Hydrocarbon and Lipid Microbiology*, ed. Geiger O. (Cham: Springer), 1–18. 10.1007/978-3-319-43676-0_4-2

[B94] Xu K. Liu H. Du G. Chen J. (2009). Real-time PCR assays targeting formyltetrahydrofolate synthetase gene to enumerate acetogens in natural and engineered environments. *Anaerobe* 15 204–213. 10.1016/j.anaerobe.2009.03.005 19328859

[B95] Yang C. (2018). Acetogen communities in the gut of herbivores and their potential role in syngas fermentation. *Fermentation* 4 1–17. 10.3390/fermentation4020040

[B96] Yang S. Liebner S. Alawi M. Ebenhöh O. Wagner D. (2014). Taxonomic database and cut-off value for processing mcrA gene 454 pyrosequencing data by MOTHUR. *J. Microbiol. Methods* 103 3–5. 10.1016/j.mimet.2014.05.006 24858450

