# Supplementary material for: High-Throughput Sequencing and Unsupervised Analysis of Formyltetrahydrofolate Synthetase (FTHFS) Gene Amplicons to Estimate Acetogenic Community Structure
Source: Front Microbiol. 2020 Aug 27;11:2066. doi: 10.3389/fmicb.2020.02066 (PMC7481360; doi:10.3389/fmicb.2020.02066)
Supplement: Supplementary file 1 [file Table_1.DOCX]

**Supplementary tables**

**Table T1**. Number of reads generated from the multiplexed samples on Illumina MiSeq. Number of fastq reads represents the raw reads and number of fasta reads represents the reads after adapter/primer cutting and quality filtering process

| Sample number | Sample name | Forward reads (R1) | | Reverse reads (R2) | |
| --- | --- | --- | --- | --- | --- |
|  |  | Number of fastq reads | Number of fasta reads | Number of fastq reads | Number of fasta reads |
| 1 | GR2_150303 | 1 278 402 | 1 262 993 | 1 278 402 | 1 210 674 |
| 2 | GR2_150414 | 1 825 726 | 1 803 465 | 1 825 726 | 1 727 875 |
| 3 | GR2_150519 | 1 237 967 | 1 223 698 | 1 237 967 | 1 174 589 |
| 4 | GR2_150709 | 1 129 891 | 1 116 172 | 1 129 891 | 1 075 076 |
| 5 | GR2_151117 | 1 107 818 | 1 093 554 | 1 107 818 | 1 048 223 |
