# Supplementary material for: High-Throughput Sequencing and Unsupervised Analysis of Formyltetrahydrofolate Synthetase (FTHFS) Gene Amplicons to Estimate Acetogenic Community Structure
Source: Front Microbiol. 2020 Aug 27;11:2066. doi: 10.3389/fmicb.2020.02066 (PMC7481360; doi:10.3389/fmicb.2020.02066)

Figure P1: Taxonomic assignments for the phylum-level dataset “full_length” at a clustering threshold of 80%

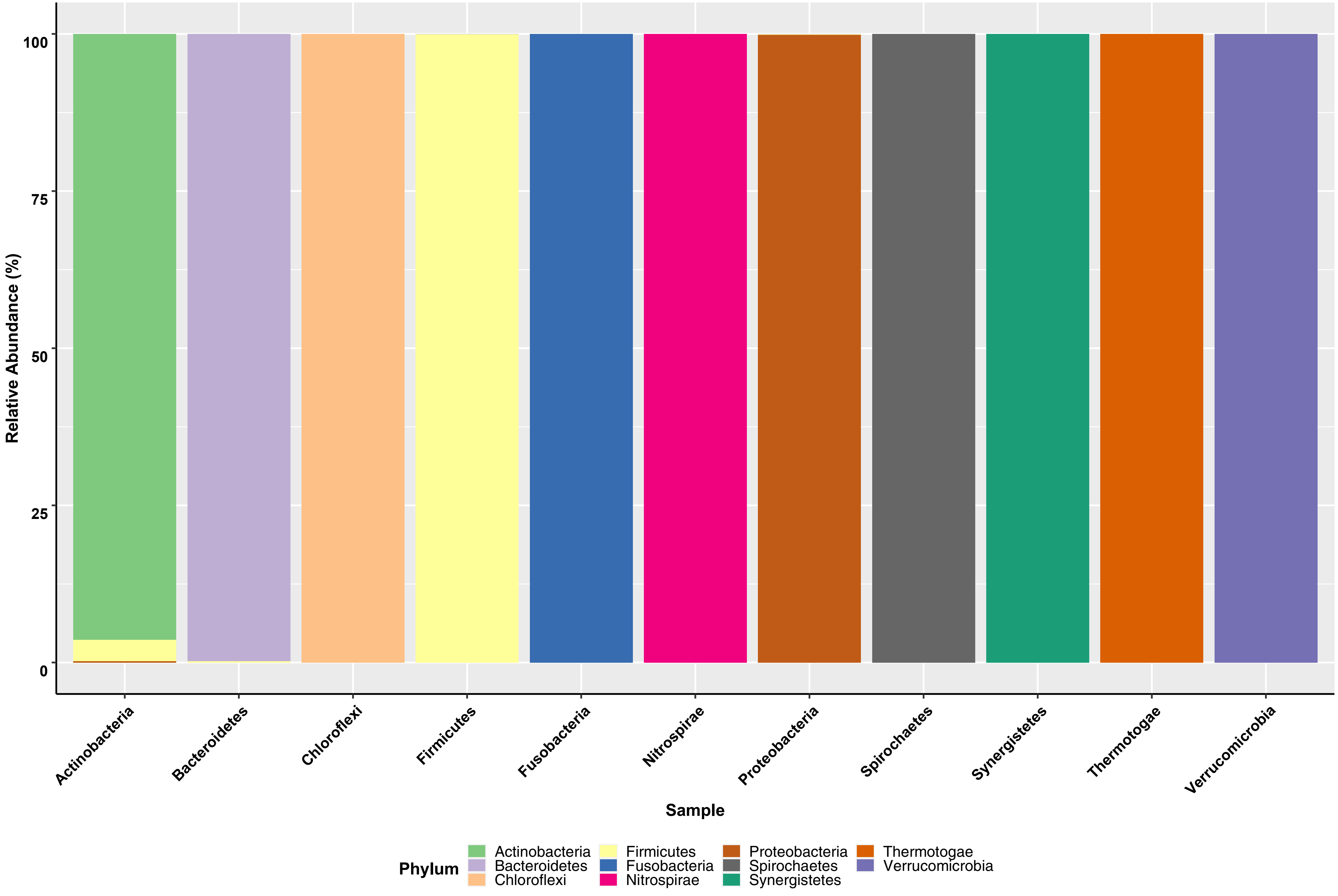

Figure P2: Taxonomic assignments for the phylum-level dataset “full_length” at a clustering threshold of 100%

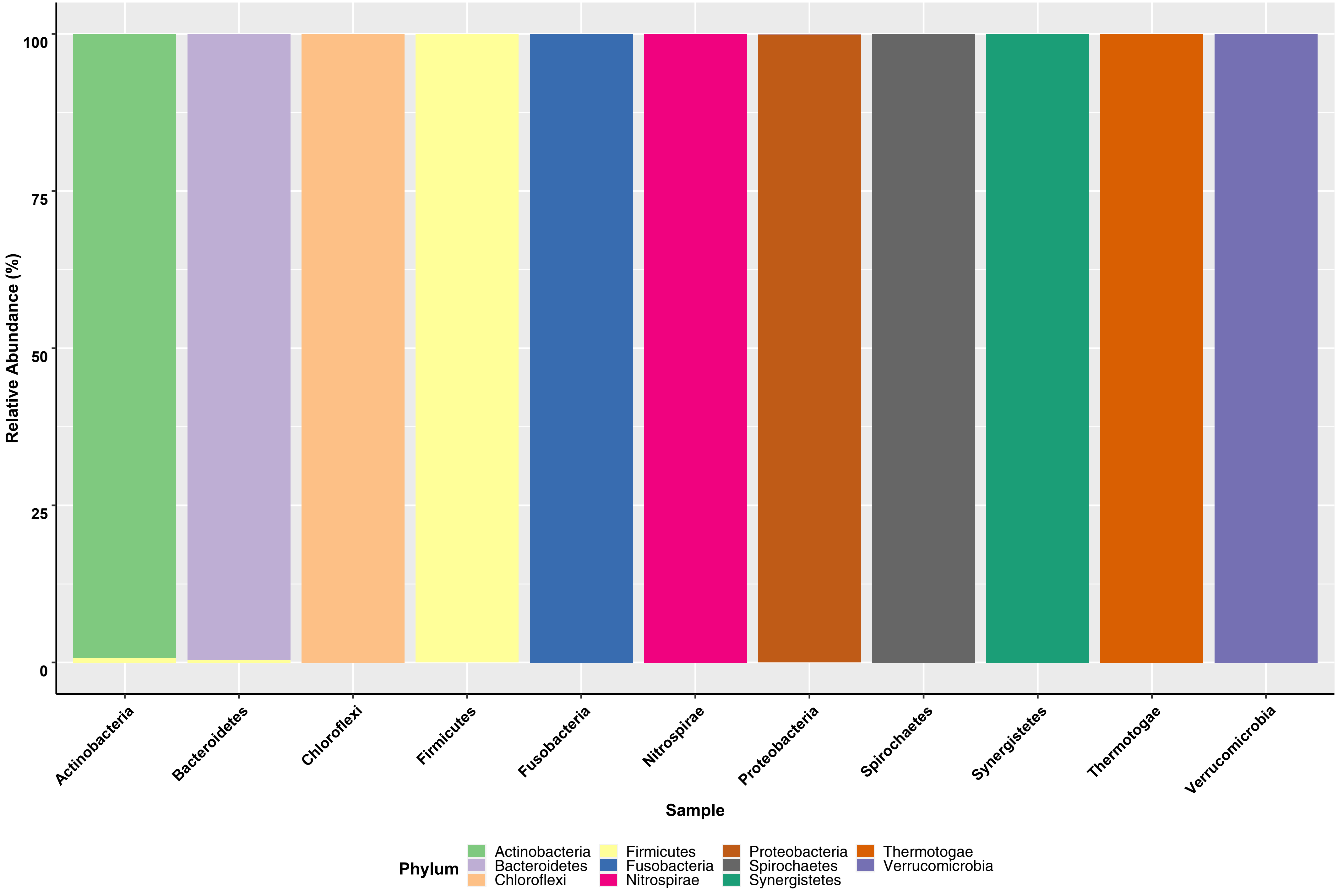

Figure P3: Taxonomic assignments for the phylum-level dataset “trimmed” at a clustering threshold of 80%

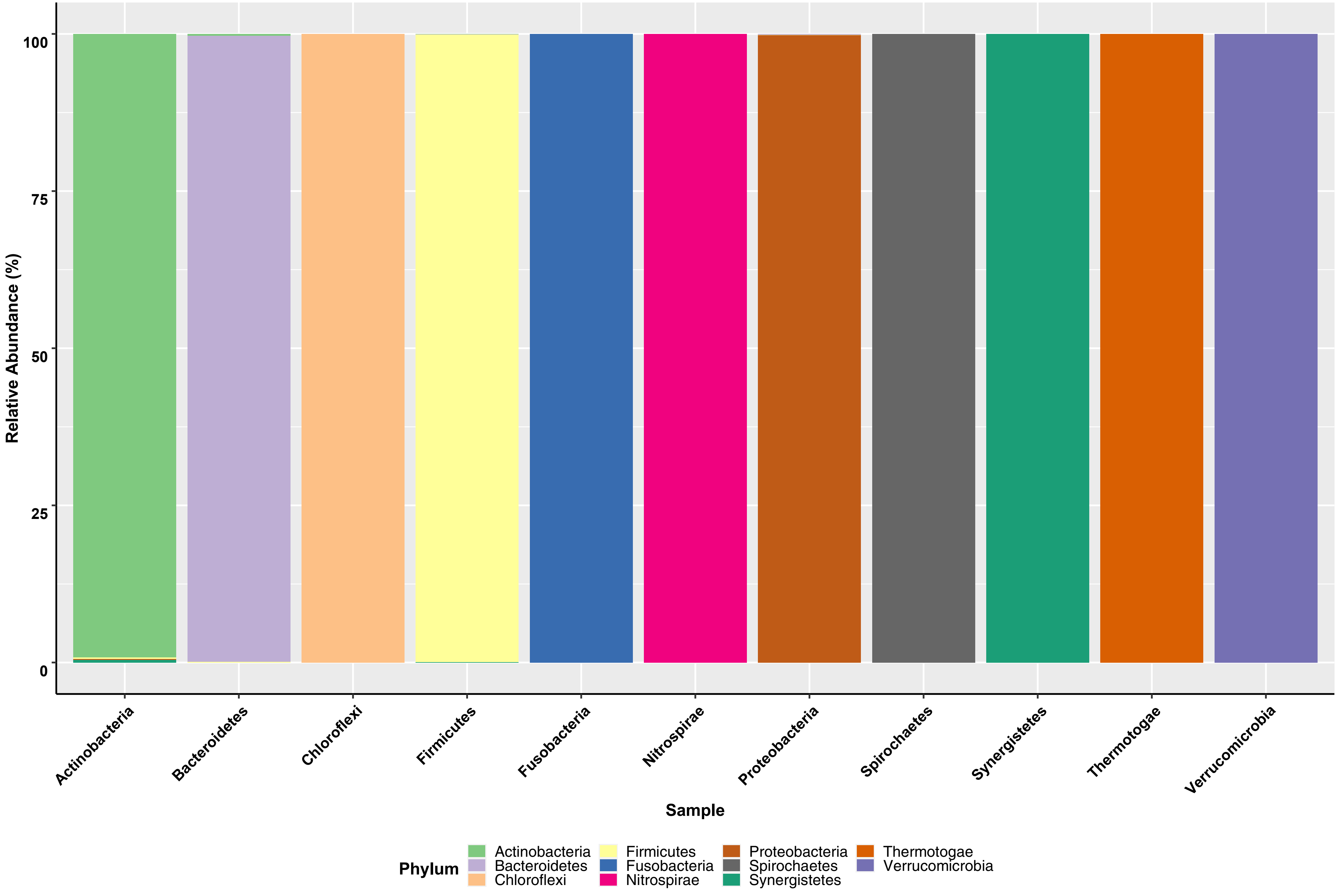

Figure P4: Taxonomic assignments for the phylum-level dataset “trimmed” at a clustering threshold of 100%

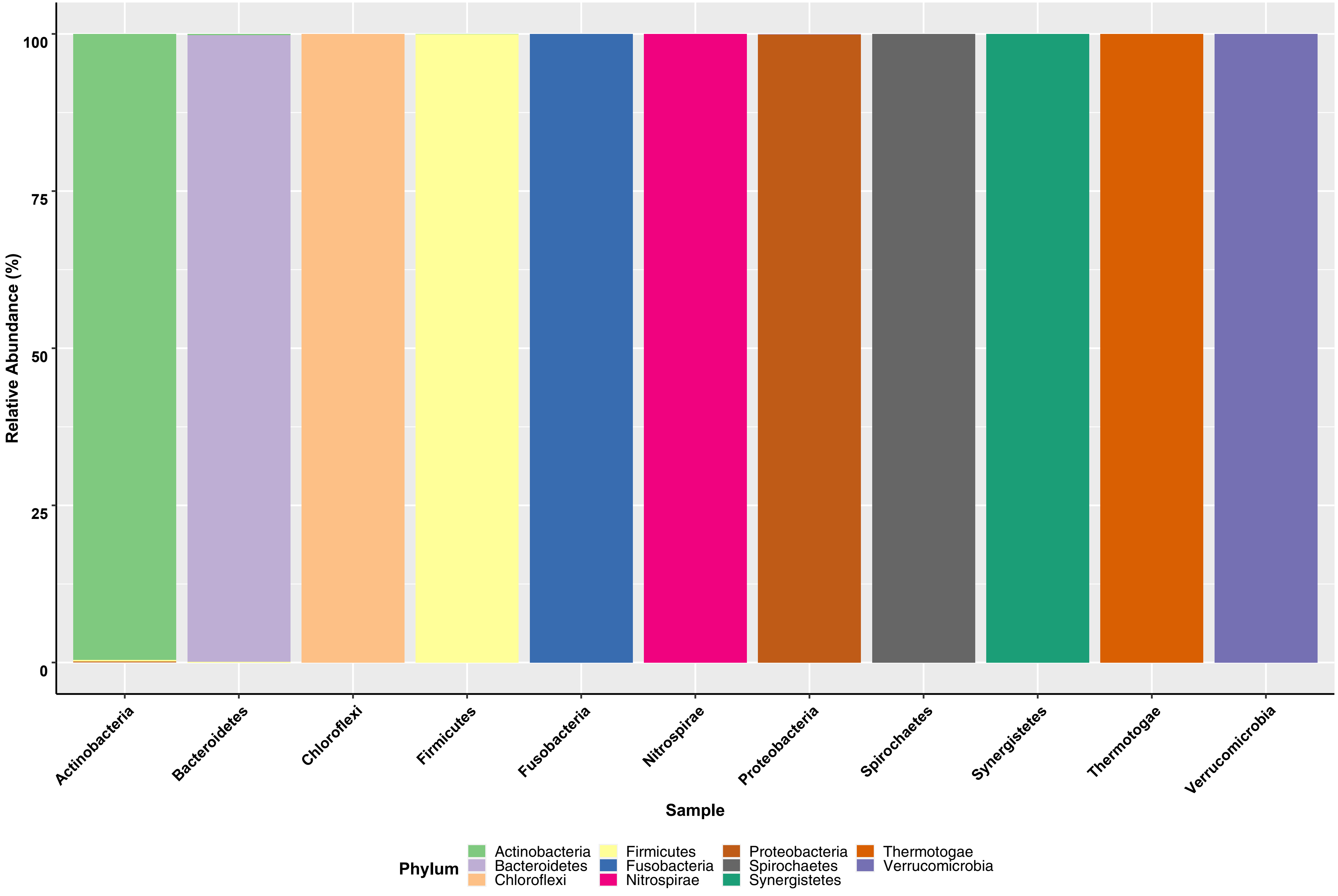

Figure P5: Taxonomic assignments for the phylum-level dataset “300” at a clustering threshold of 80%

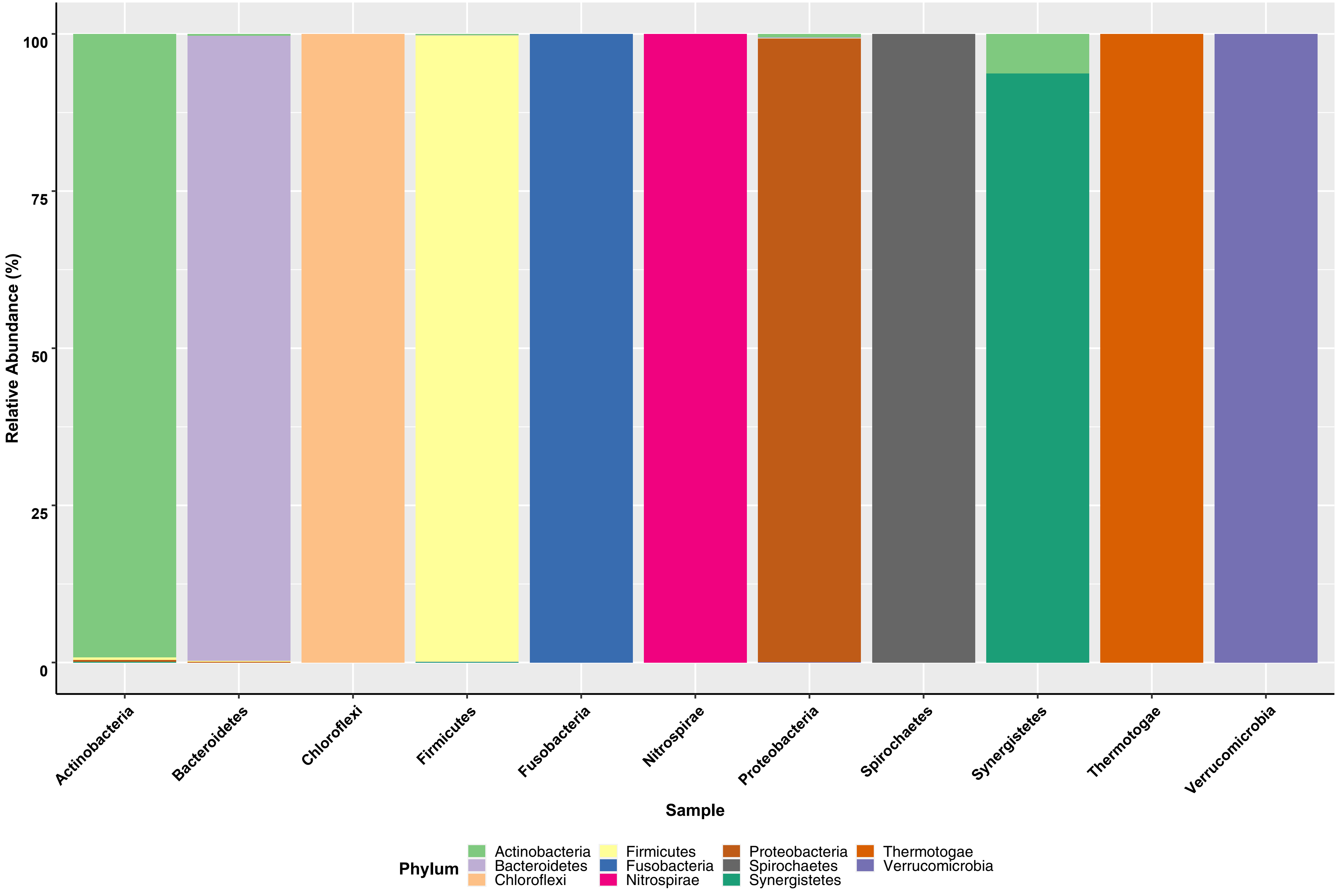

Figure P6: Taxonomic assignments for the phylum-level dataset “300” at a clustering threshold of 100%

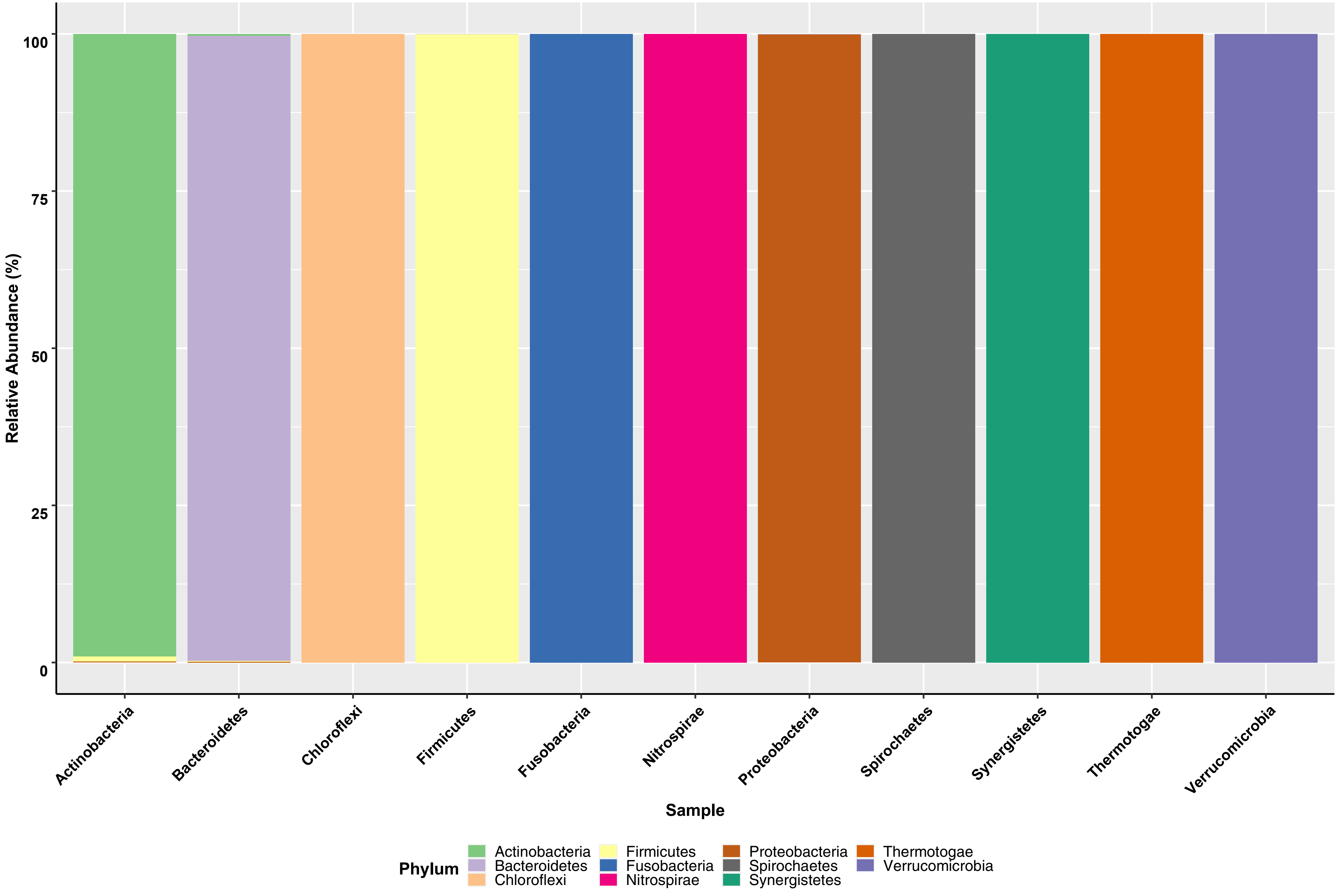

Figure P7: Taxonomic assignments for the phylum-level dataset “150” at a clustering threshold of 80%

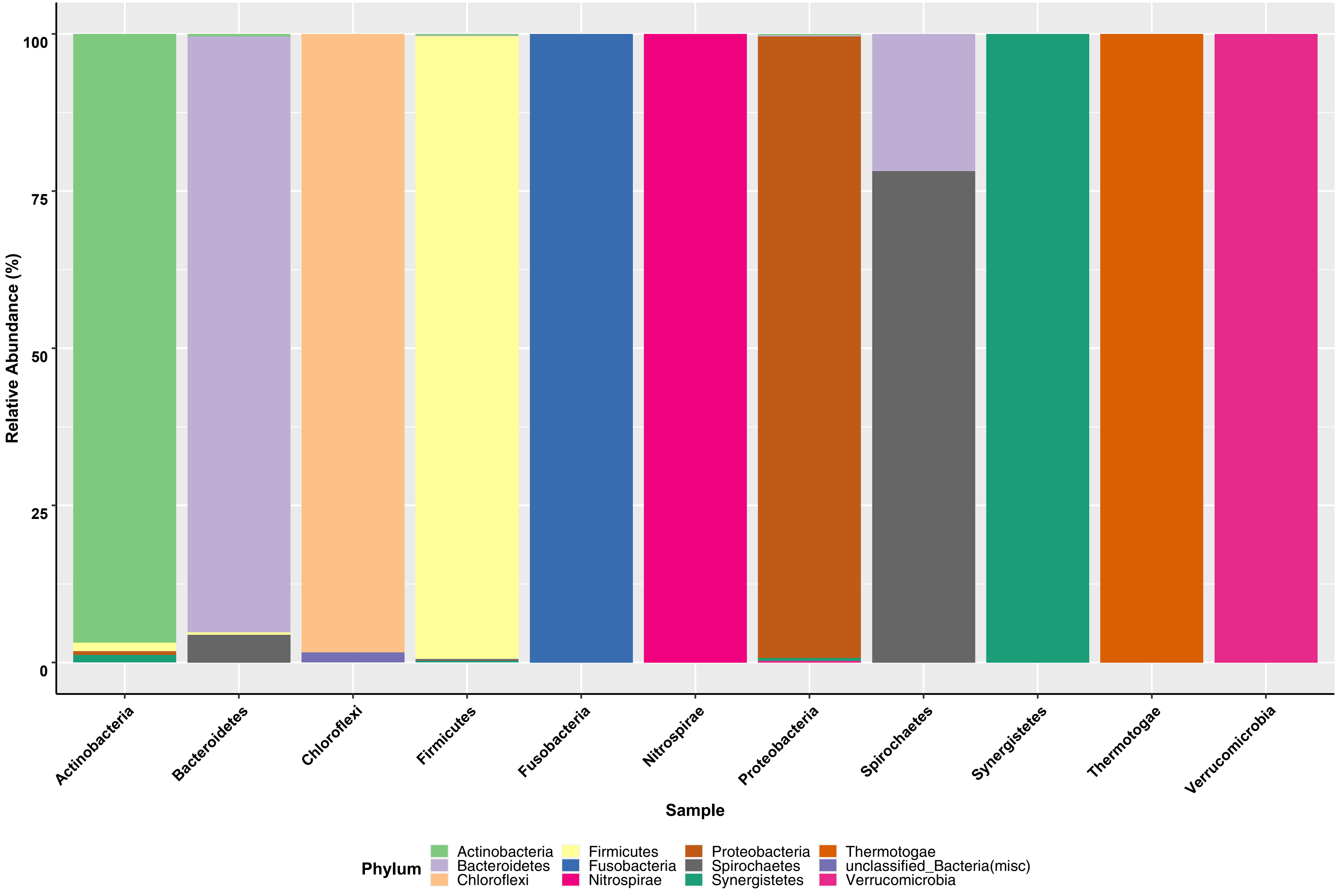

Figure P8: Taxonomic assignments for the phylum-level dataset “150” at a clustering threshold of 100%

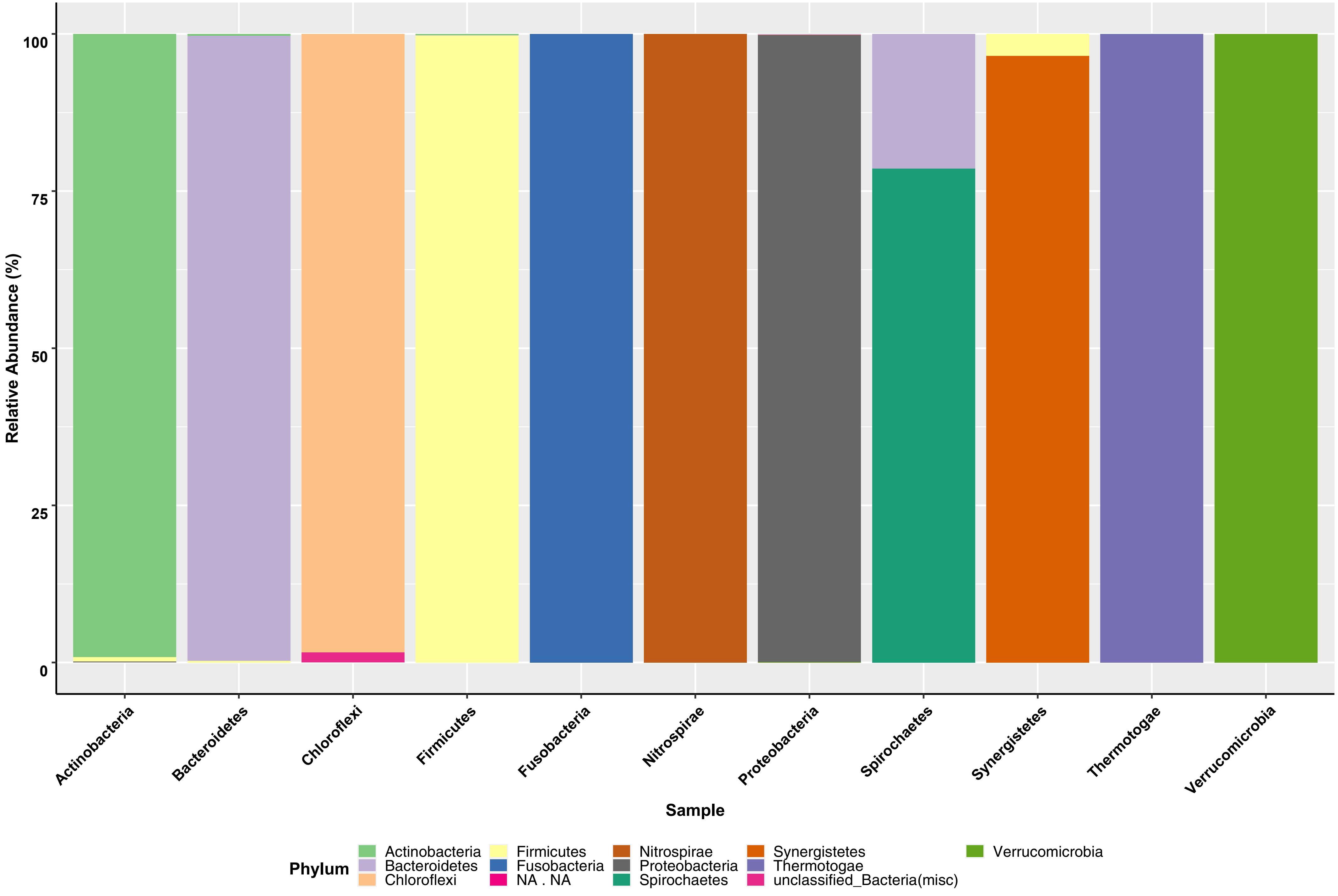

Supplement: Supplementary file 2 [file Data_Sheet_1.PDF]
