# Supplementary material for: High-Throughput Sequencing and Unsupervised Analysis of Formyltetrahydrofolate Synthetase (FTHFS) Gene Amplicons to Estimate Acetogenic Community Structure
Source: Front Microbiol. 2020 Aug 27;11:2066. doi: 10.3389/fmicb.2020.02066 (PMC7481360; doi:10.3389/fmicb.2020.02066)

Figure F1: Taxonomic assignments for the family-level dataset “full_length” at a clustering threshold of 80 %

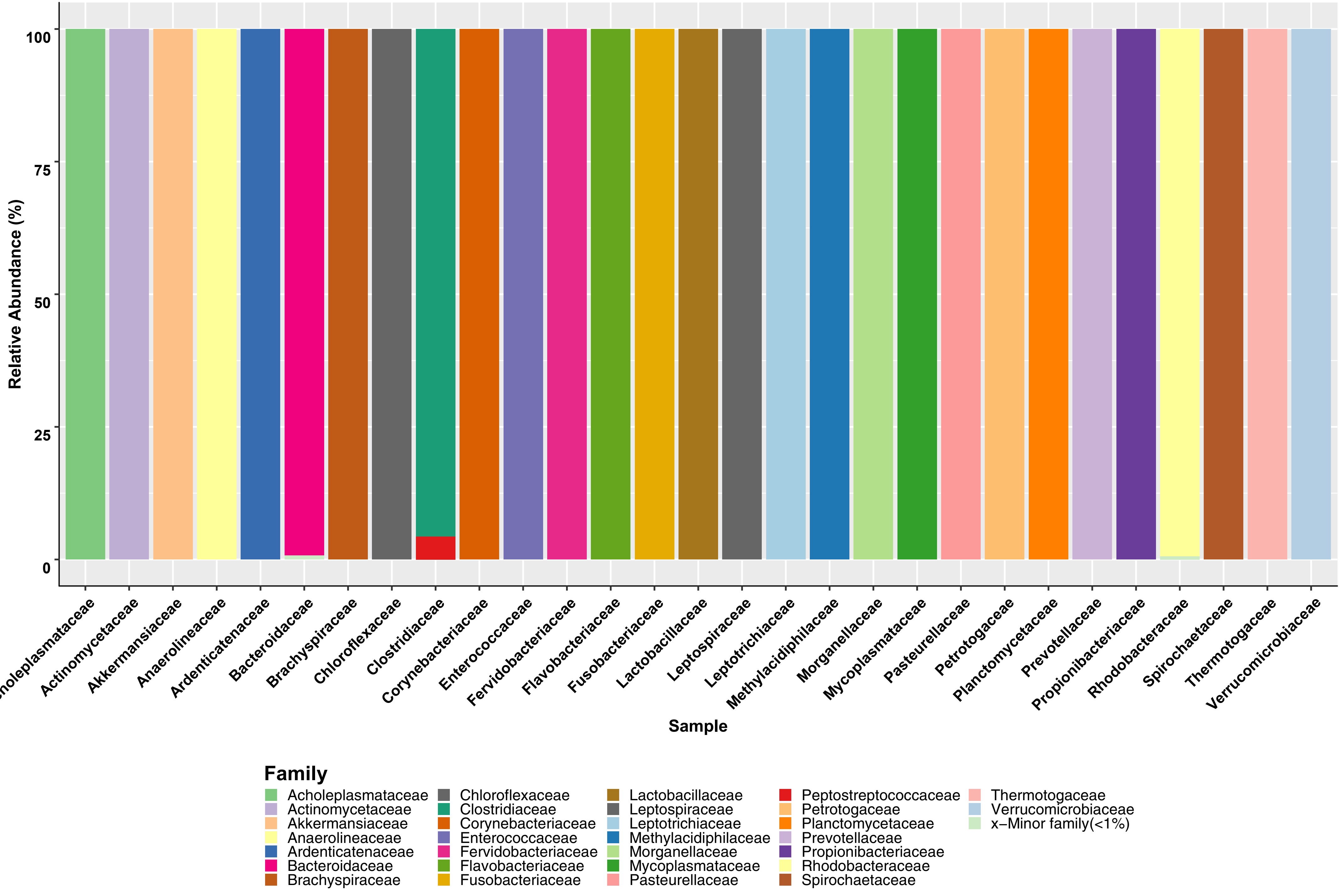

Figure F2: Taxonomic assignments for the family-level dataset “full_length” at a clustering threshold of 100 %

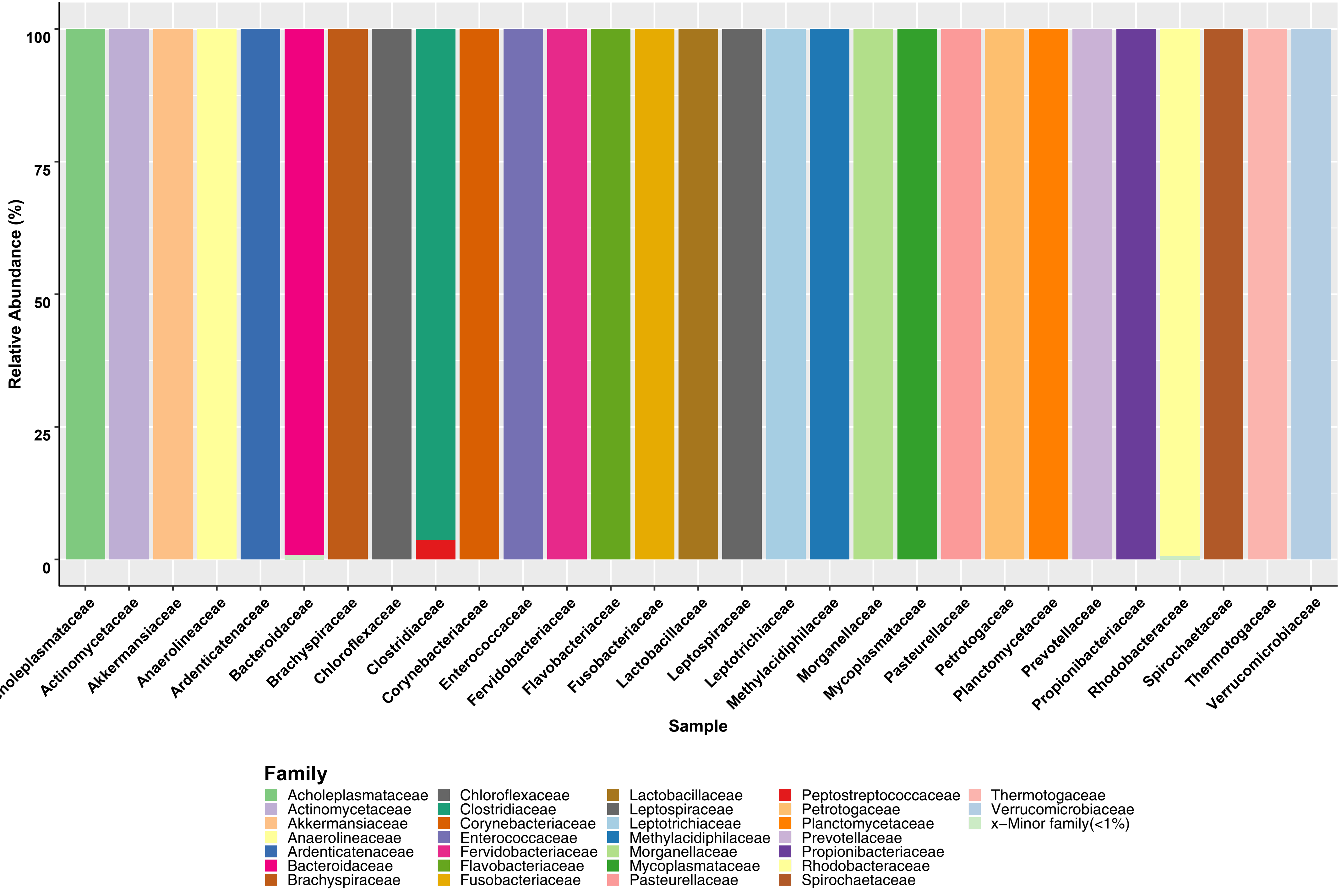

Figure F3: Taxonomic assignments for the family-level dataset “trimmed” at a clustering threshold of 80 %

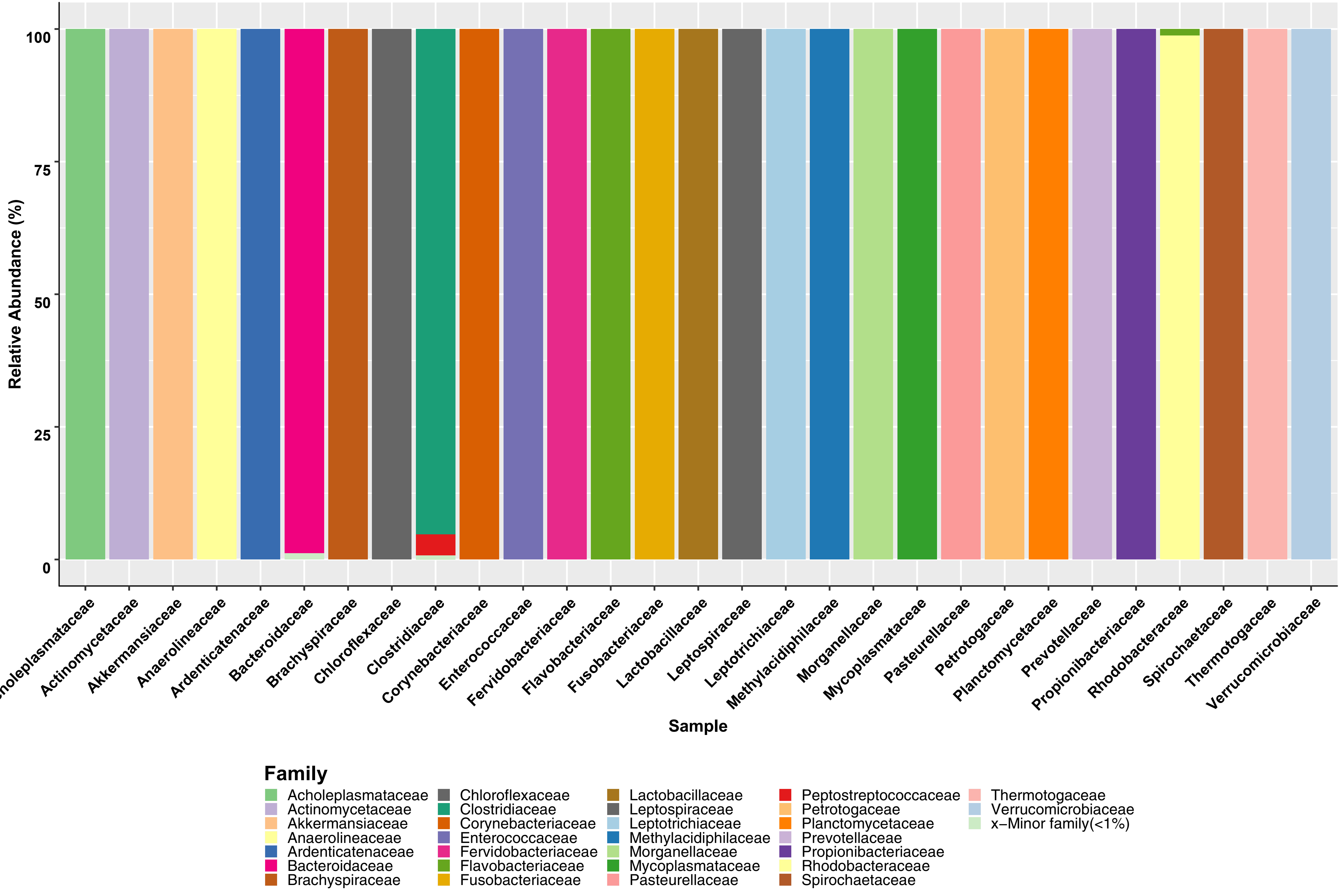

Figure F4: Taxonomic assignments for the family-level dataset ”trimmed” at a clustering threshold of 100 %

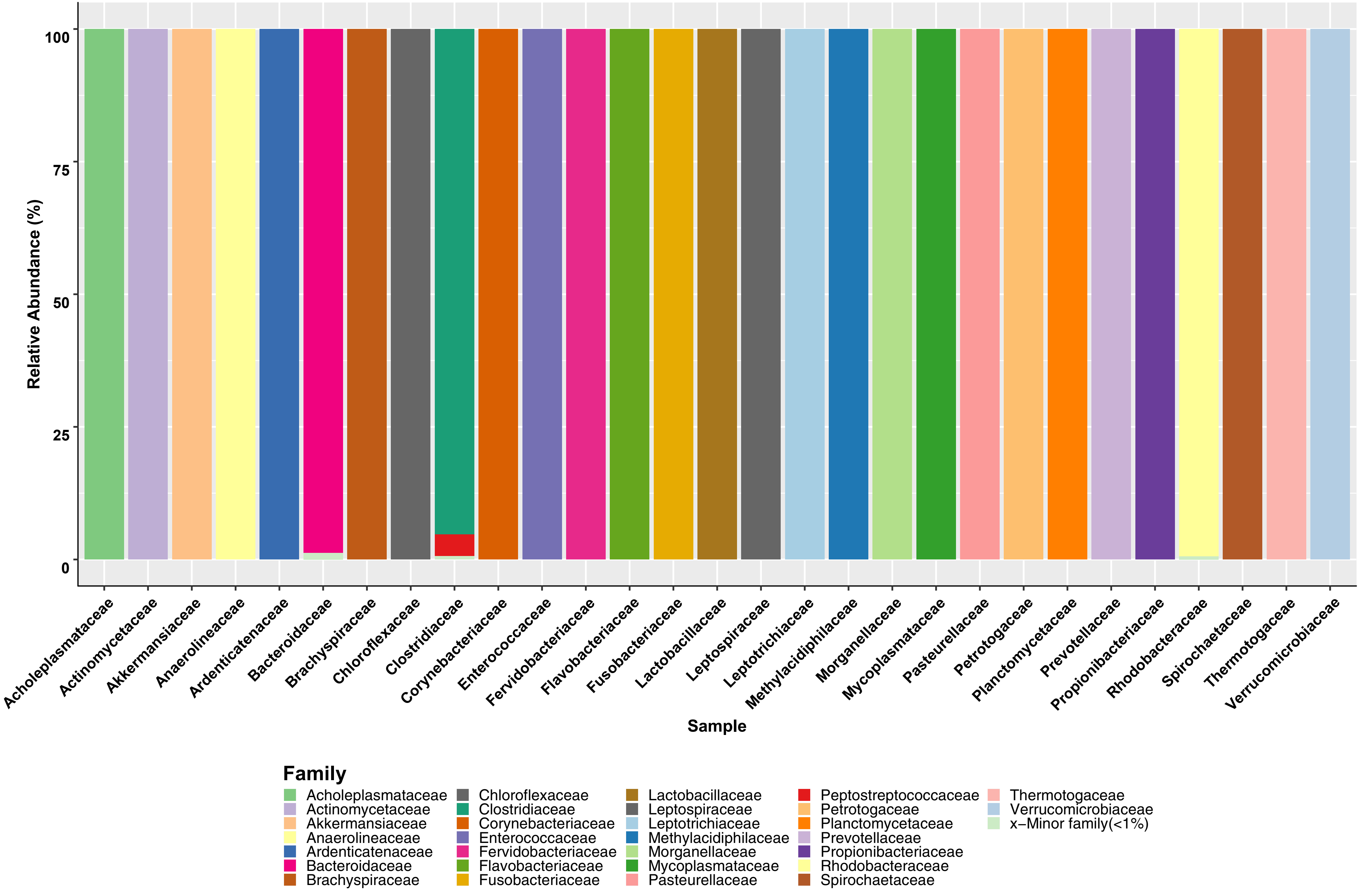

Figure F5: Taxonomic assignments for the family-level dataset ”300” at a clustering threshold of 80 %

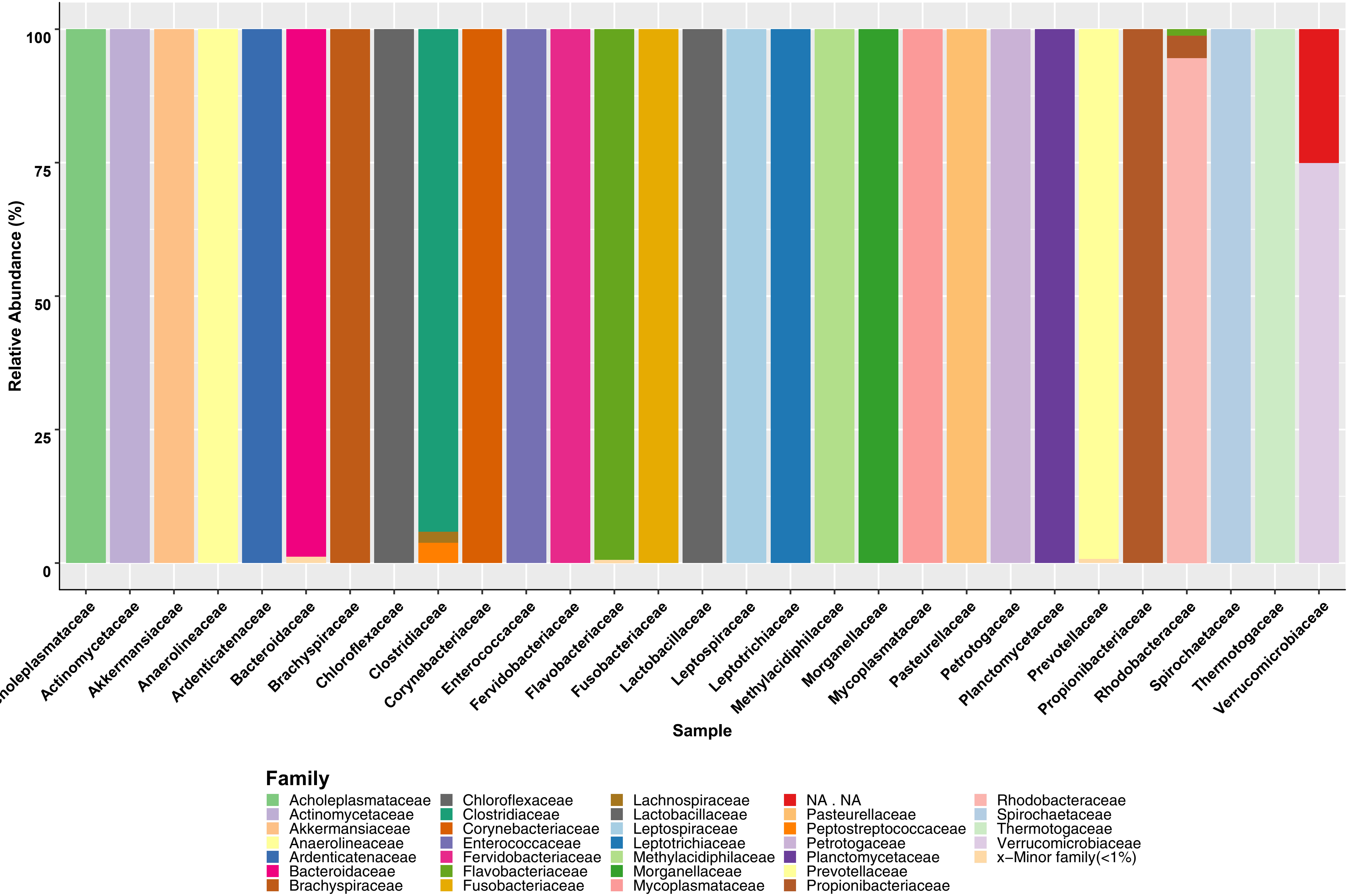

Figure F6: Taxonomic assignments for the family-level dataset ”300” at a clustering threshold of 100 %

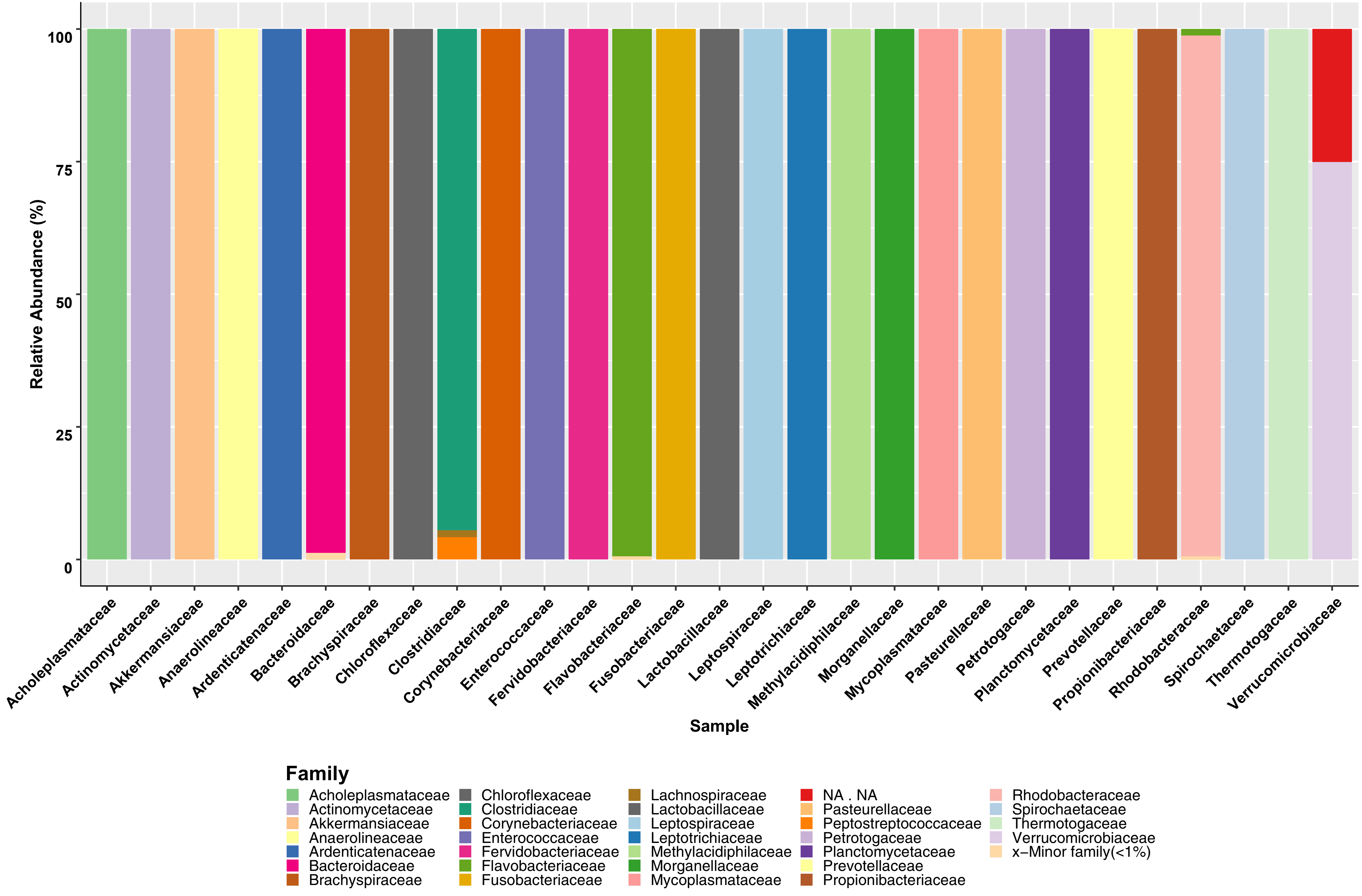

Figure F7: Taxonomic assignments for the family-level dataset "150" at a clustering threshold of 80 %

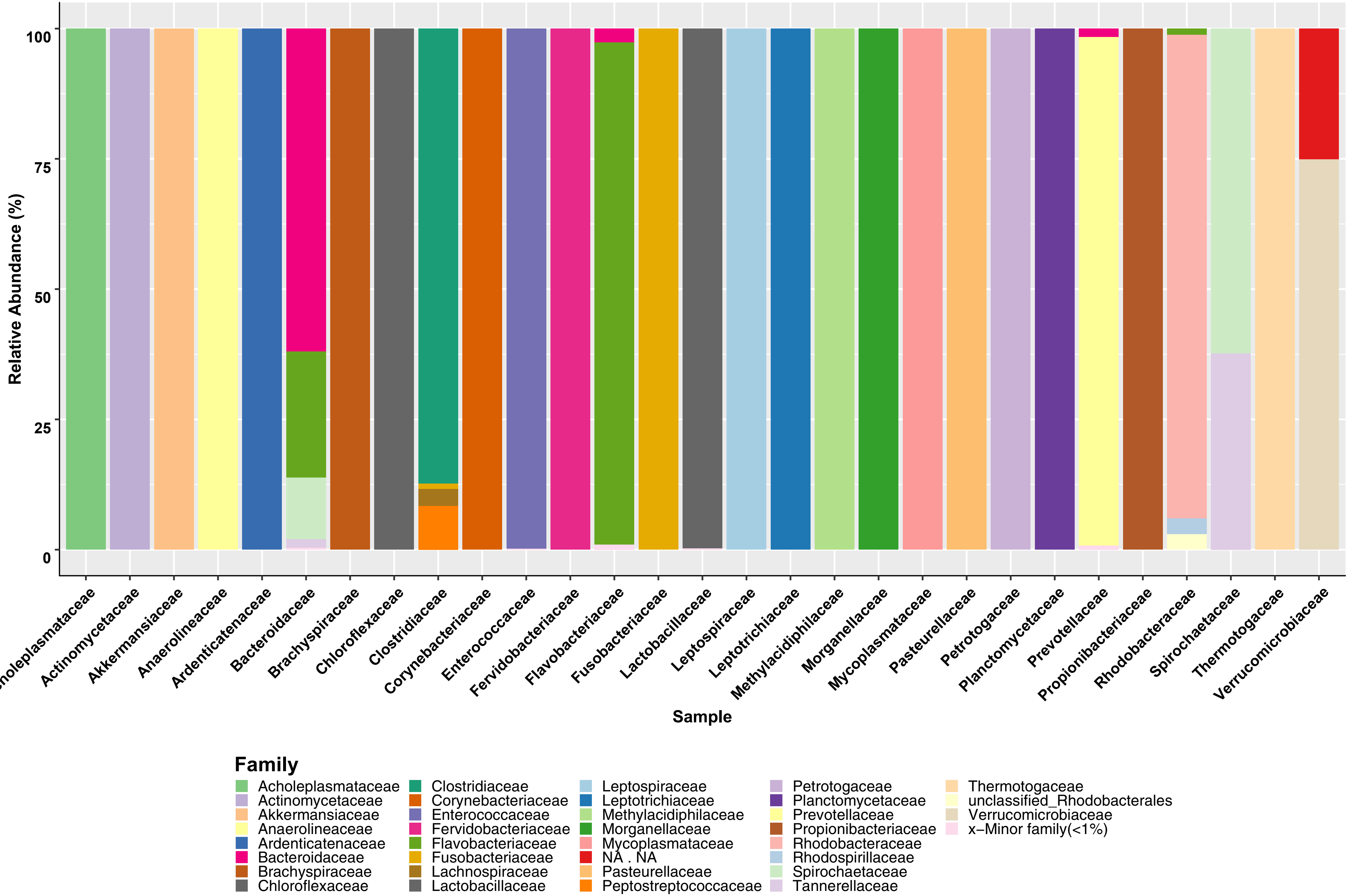

Figure F8: Taxonomic assignments for the family-level dataset ”150” at a clustering threshold of 100 %

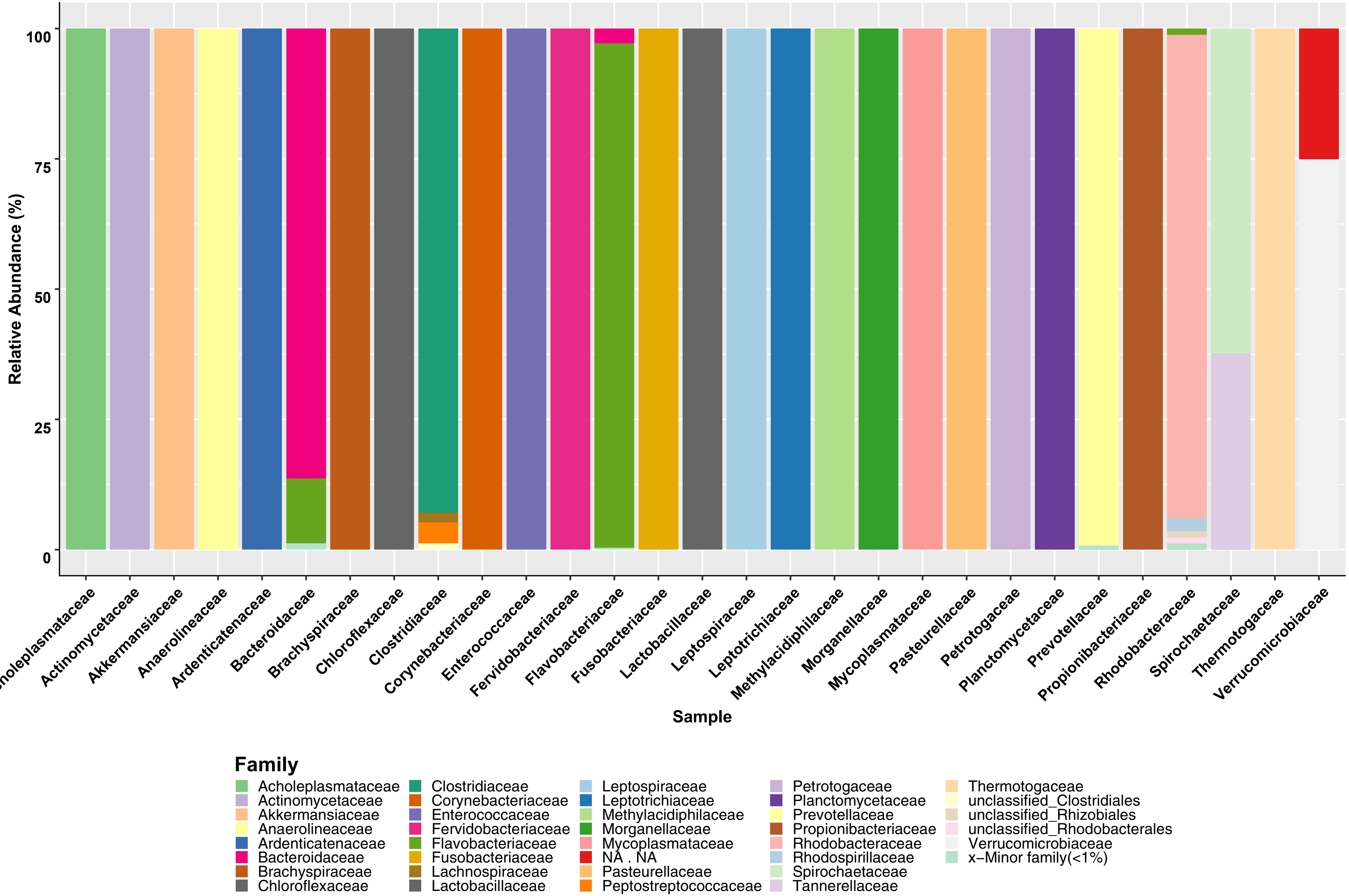

Supplement: Supplementary file 3 [file Data_Sheet_2.PDF]
