# Supplementary material for: High-Throughput Sequencing and Unsupervised Analysis of Formyltetrahydrofolate Synthetase (FTHFS) Gene Amplicons to Estimate Acetogenic Community Structure
Source: Front Microbiol. 2020 Aug 27;11:2066. doi: 10.3389/fmicb.2020.02066 (PMC7481360; doi:10.3389/fmicb.2020.02066)

Figure A1: Taxonomic assignments for the acetogen-level data-set “full_length” at a clustering threshold of 80 %

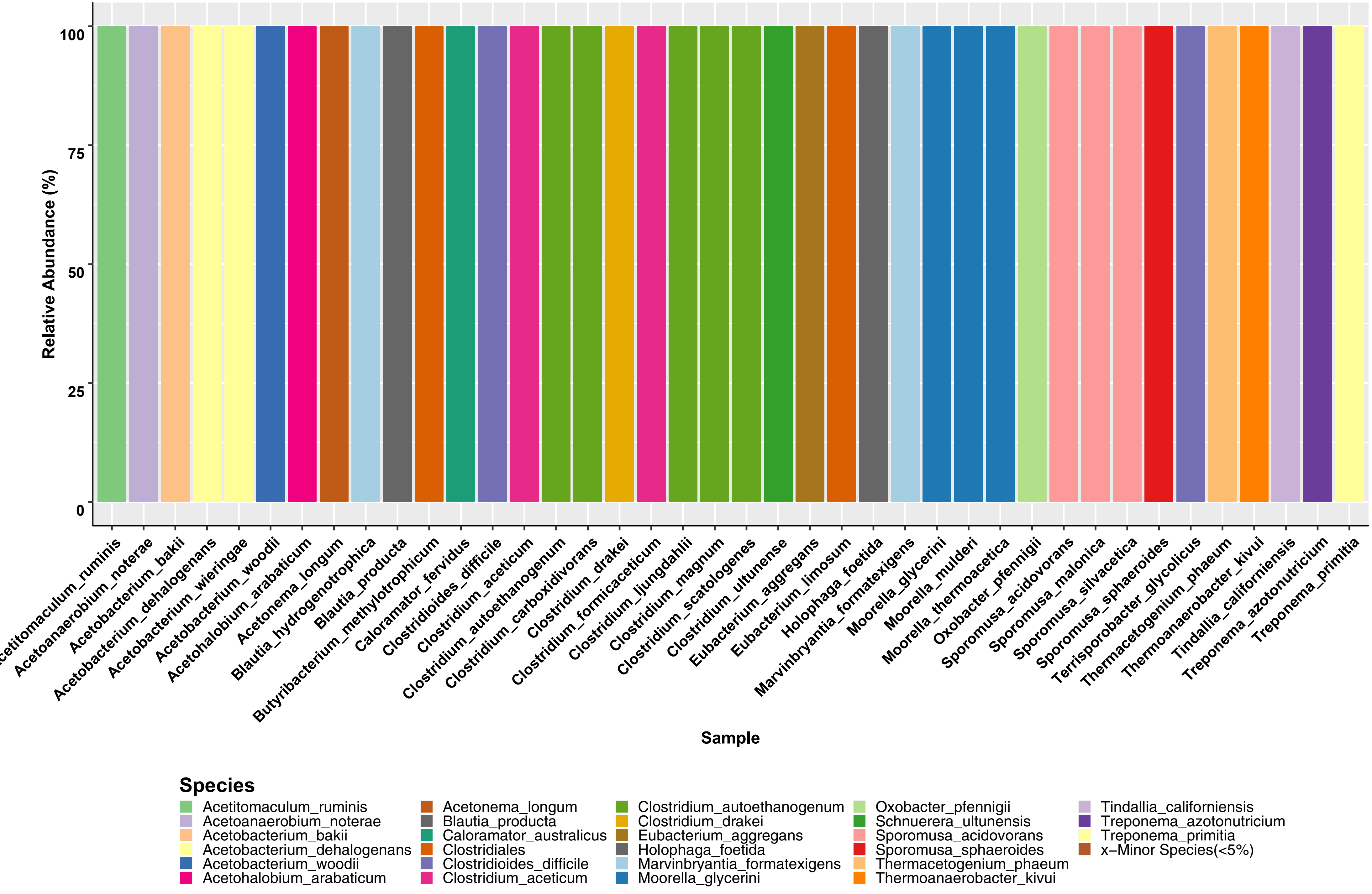

Figure A2: Taxonomic assignments for the acetogen-level data-set “full_length” at a clustering threshold of 100 %

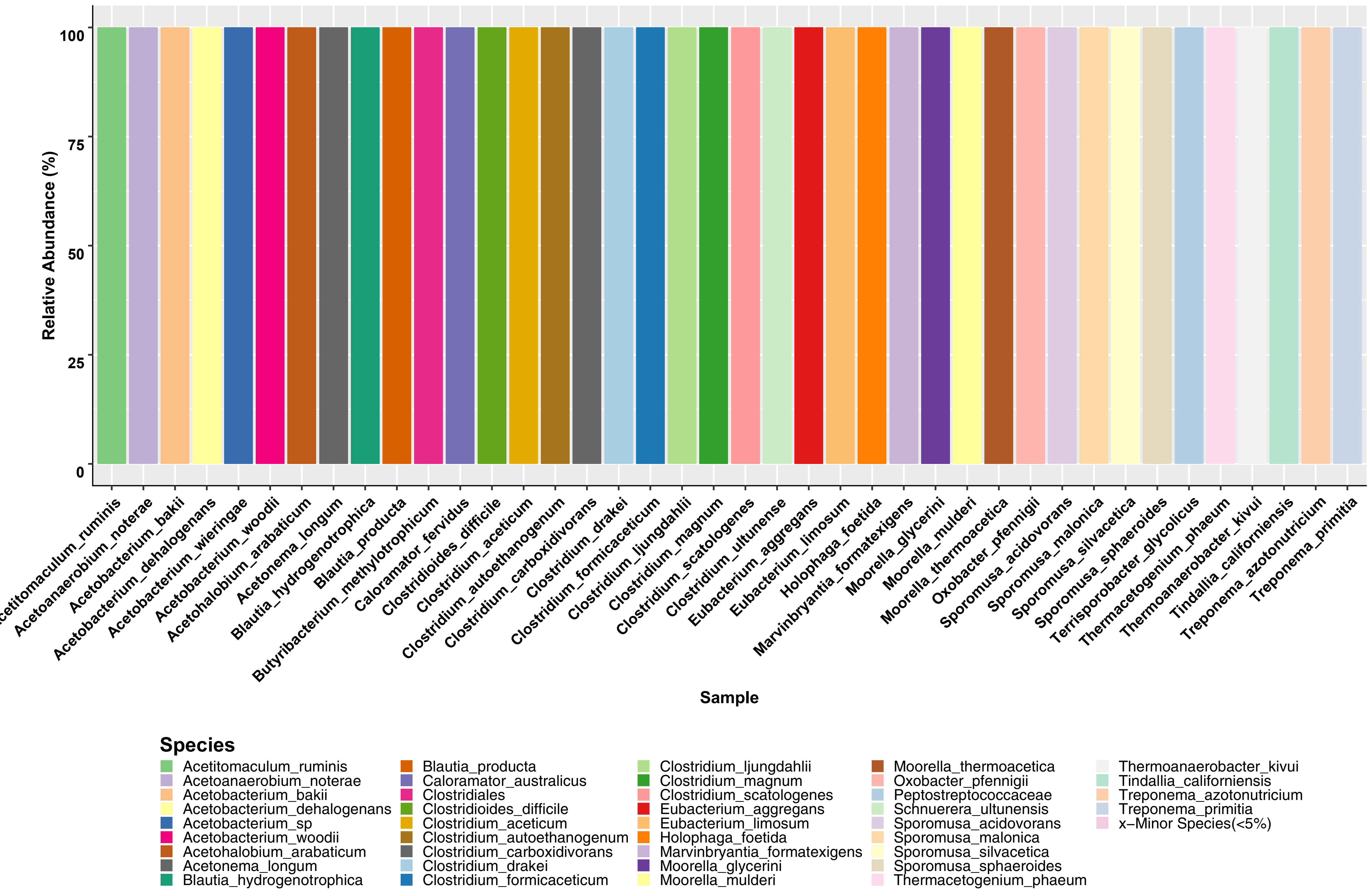

Figure A3: Taxonomic assignments for the acetogen-level data-set “trimmed” at a clustering threshold of 80 %

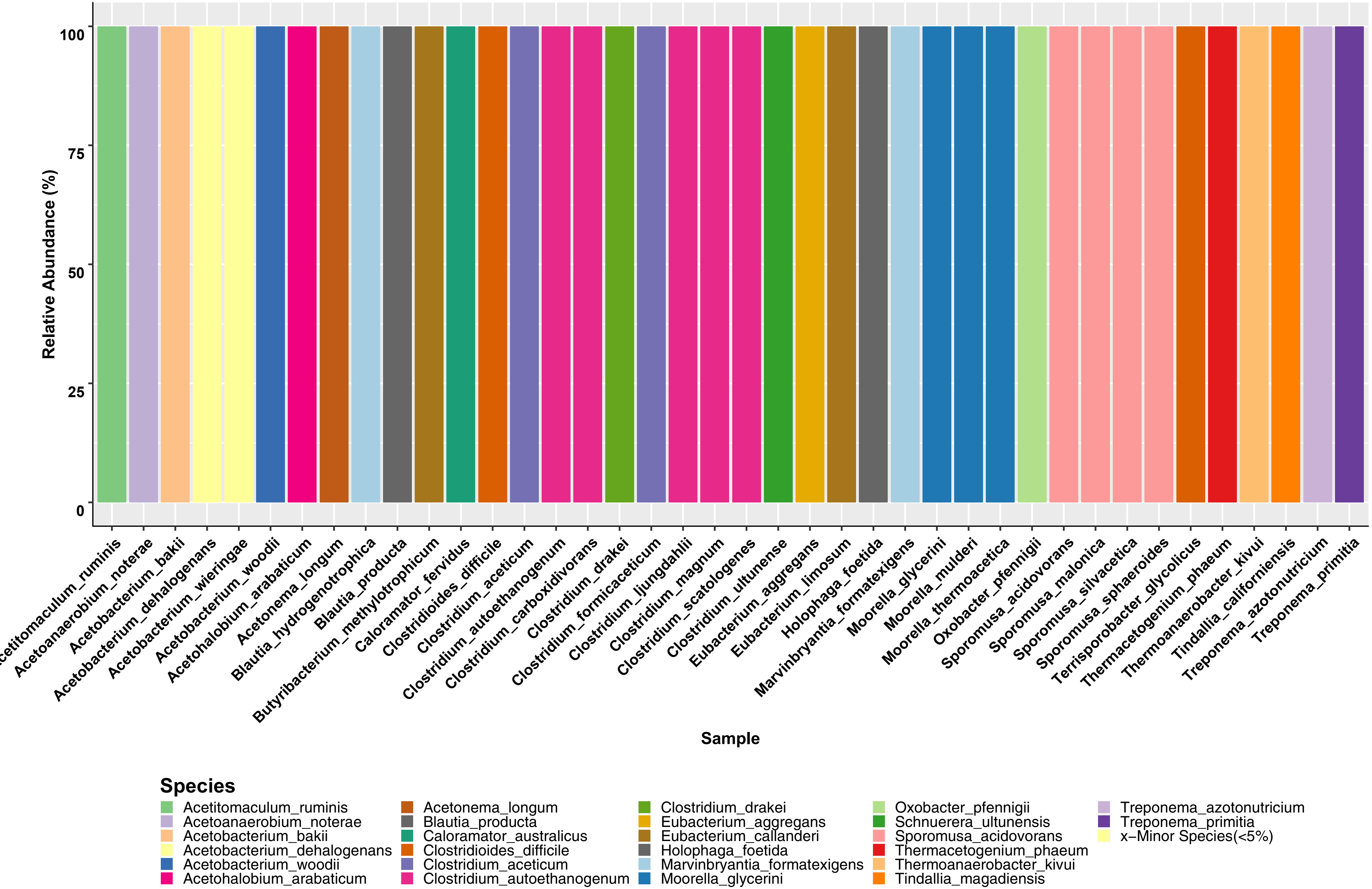

Figure A4: Taxonomic assignments for the acetogen-level data-set “trimmed” at a clustering threshold of 100 %

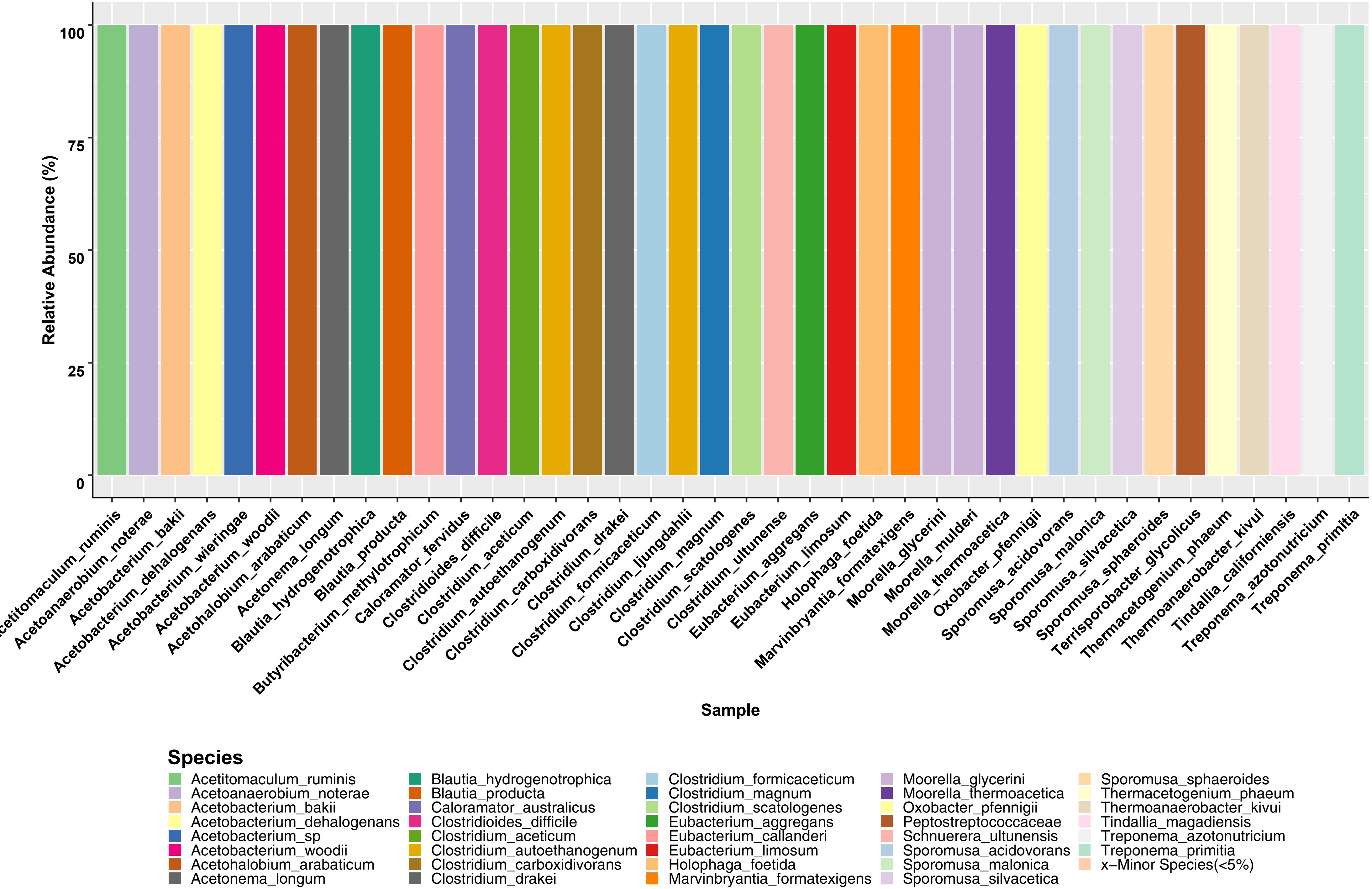

Figure A5: Taxonomic assignments for the acetogen-level data-set “300” at a clustering threshold of 80 %

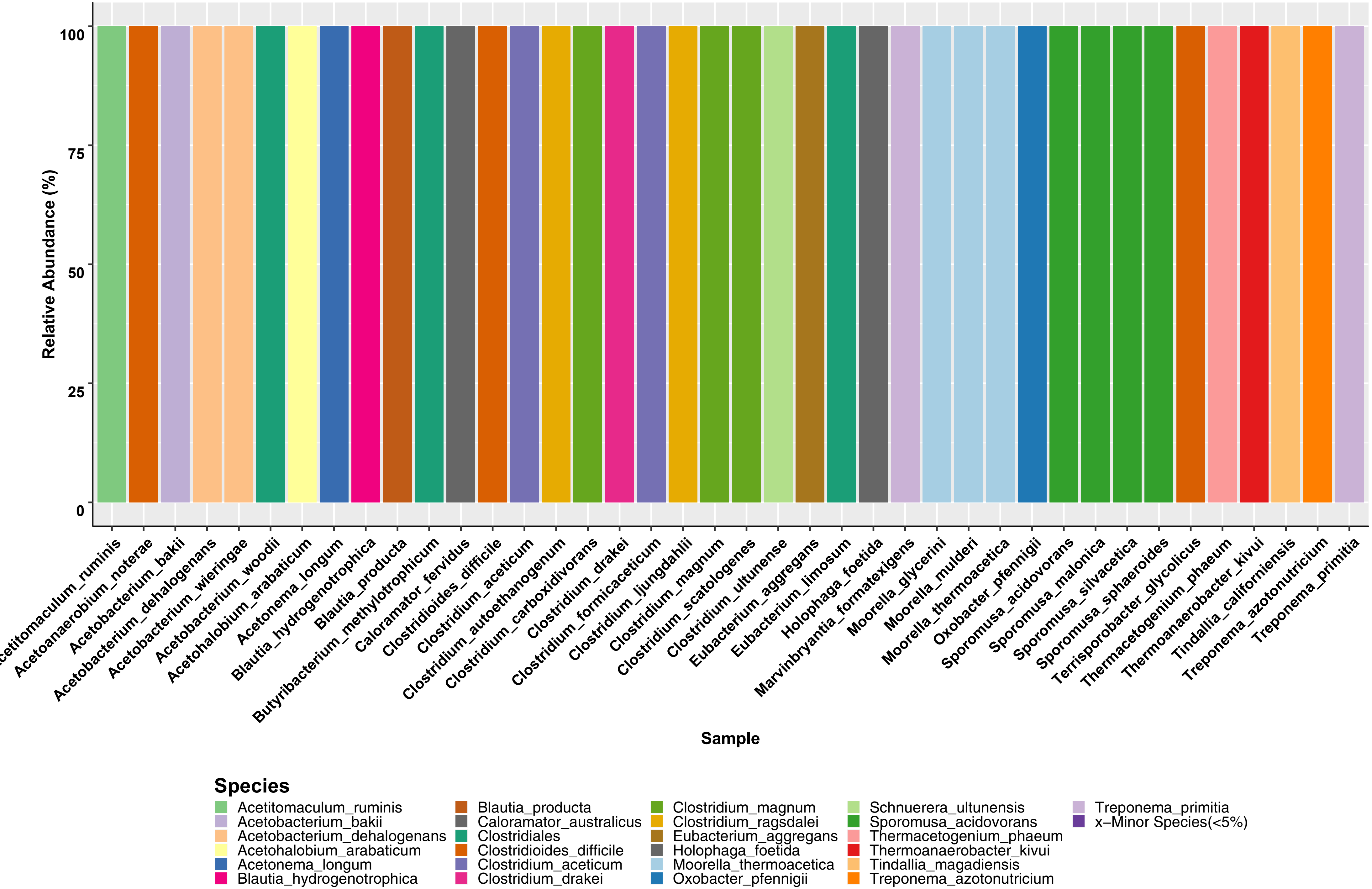

Figure A6: Taxonomic assignments for the acetogen-level data-set “300” at a clustering threshold of 100 %

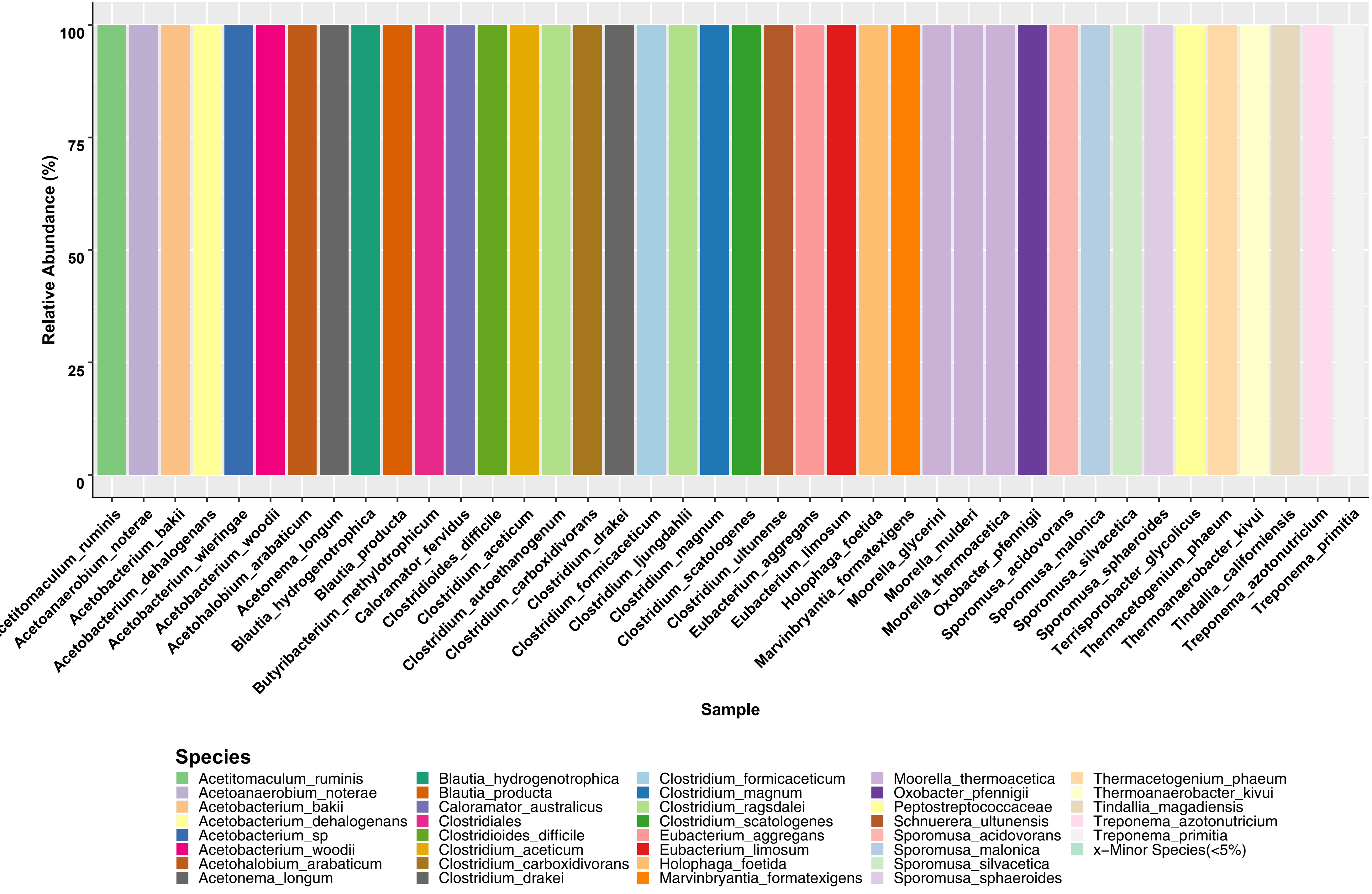

Figure A7: Taxonomic assignments for the acetogen-level data-set “150” at a clustering threshold of 80 %

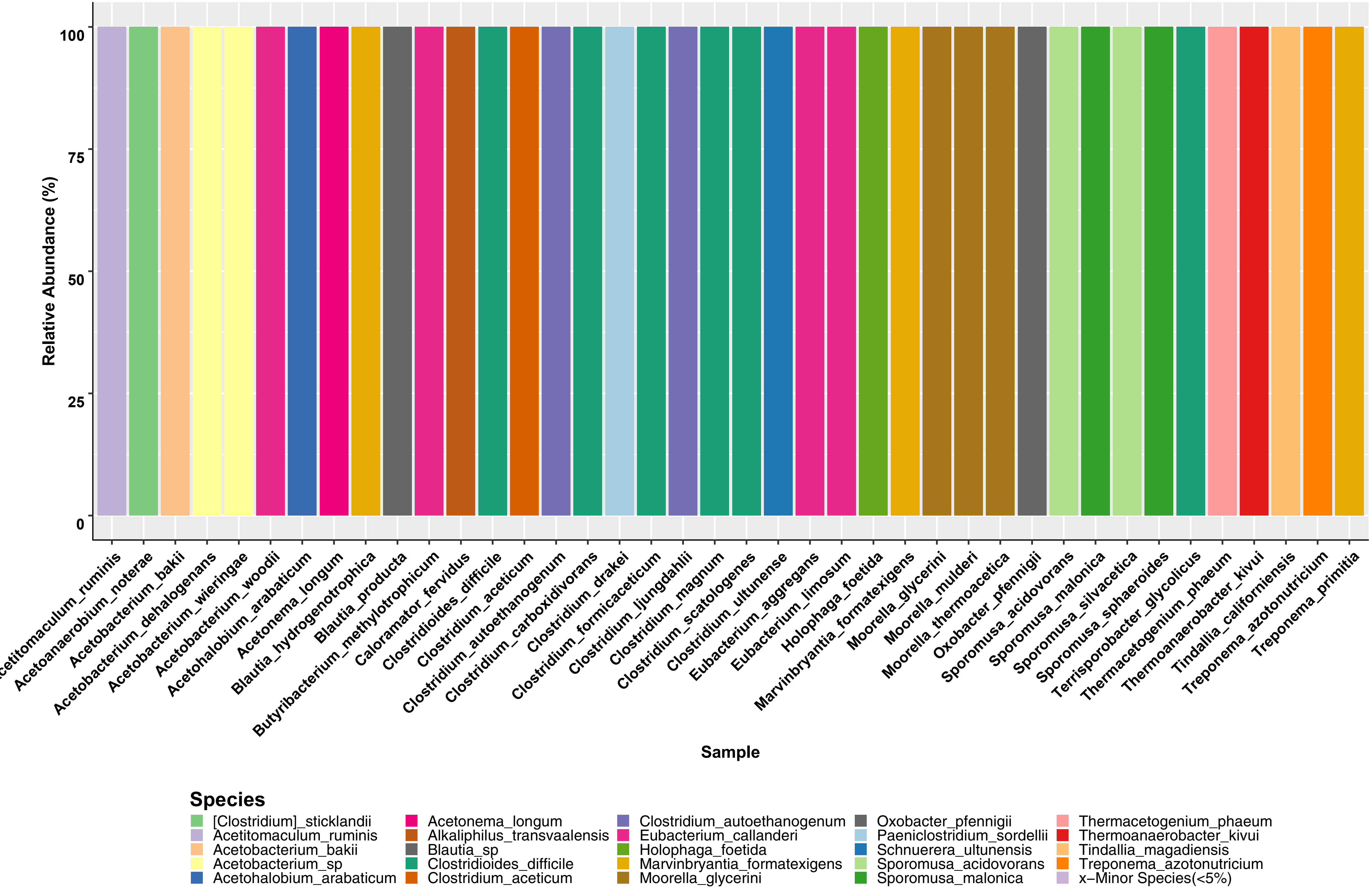

Figure A8: Taxonomic assignments for the acetogen-level data-set “150” at a clustering threshold of 100 %

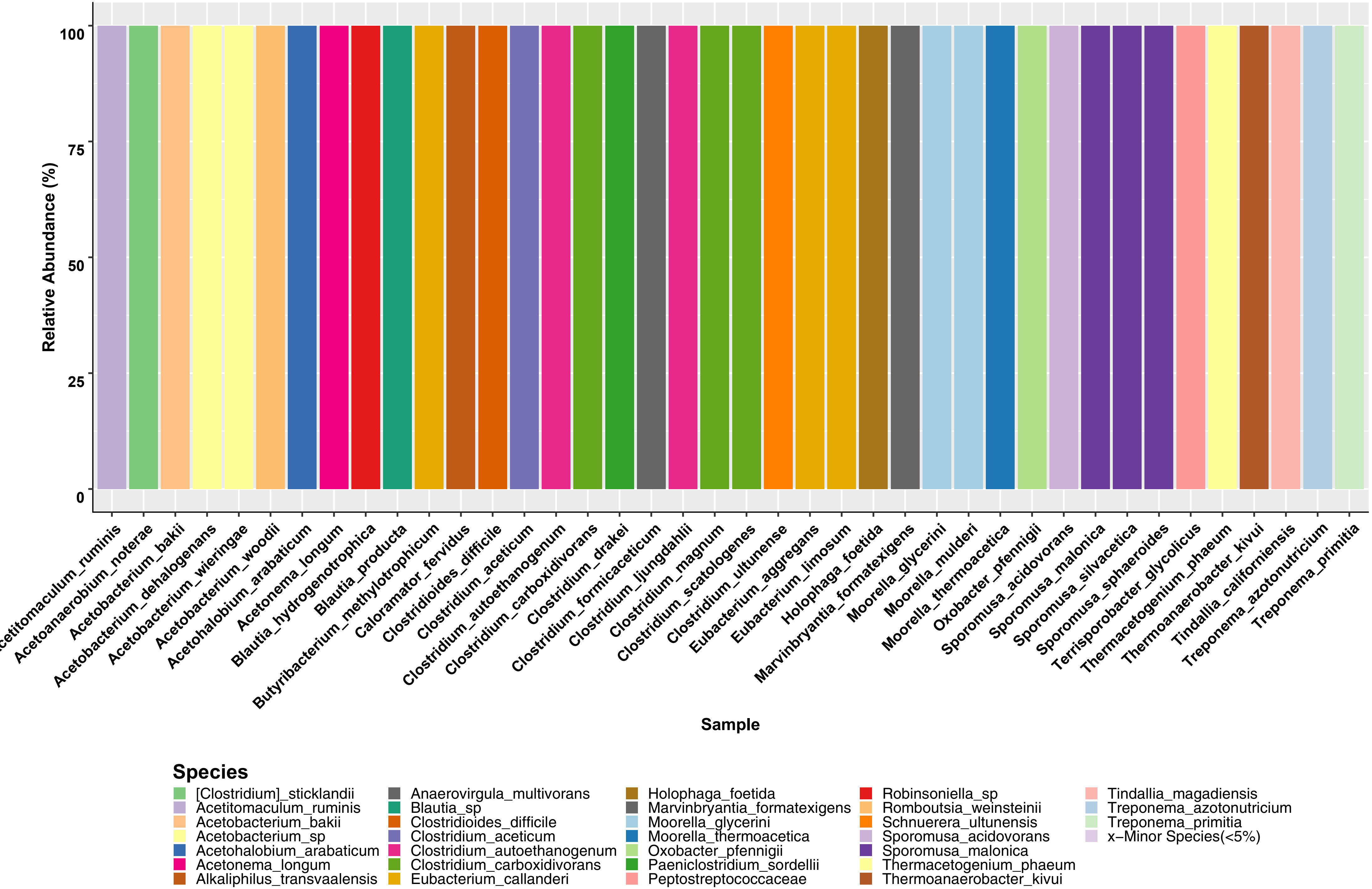

Supplement: Supplementary file 4 [file Data_Sheet_3.PDF]
