# Supplementary material for: High-Throughput Sequencing and Unsupervised Analysis of Formyltetrahydrofolate Synthetase (FTHFS) Gene Amplicons to Estimate Acetogenic Community Structure
Source: Front Microbiol. 2020 Aug 27;11:2066. doi: 10.3389/fmicb.2020.02066 (PMC7481360; doi:10.3389/fmicb.2020.02066)

Figure B1 – Phylum level taxonomic assignments of the FTHFS gene sequence data analysis of forward reads with AcetoScan at clustering threshold 80 %

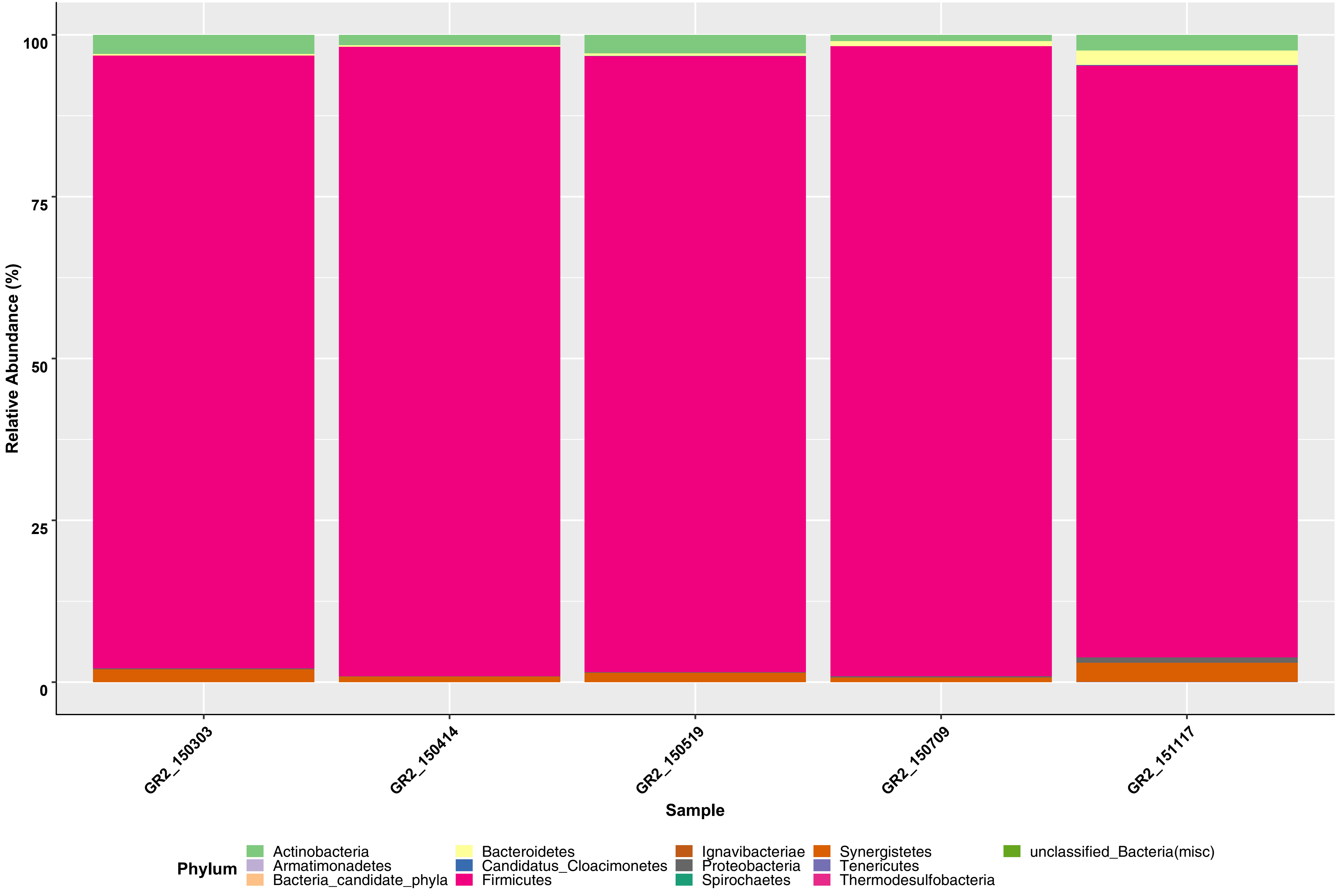

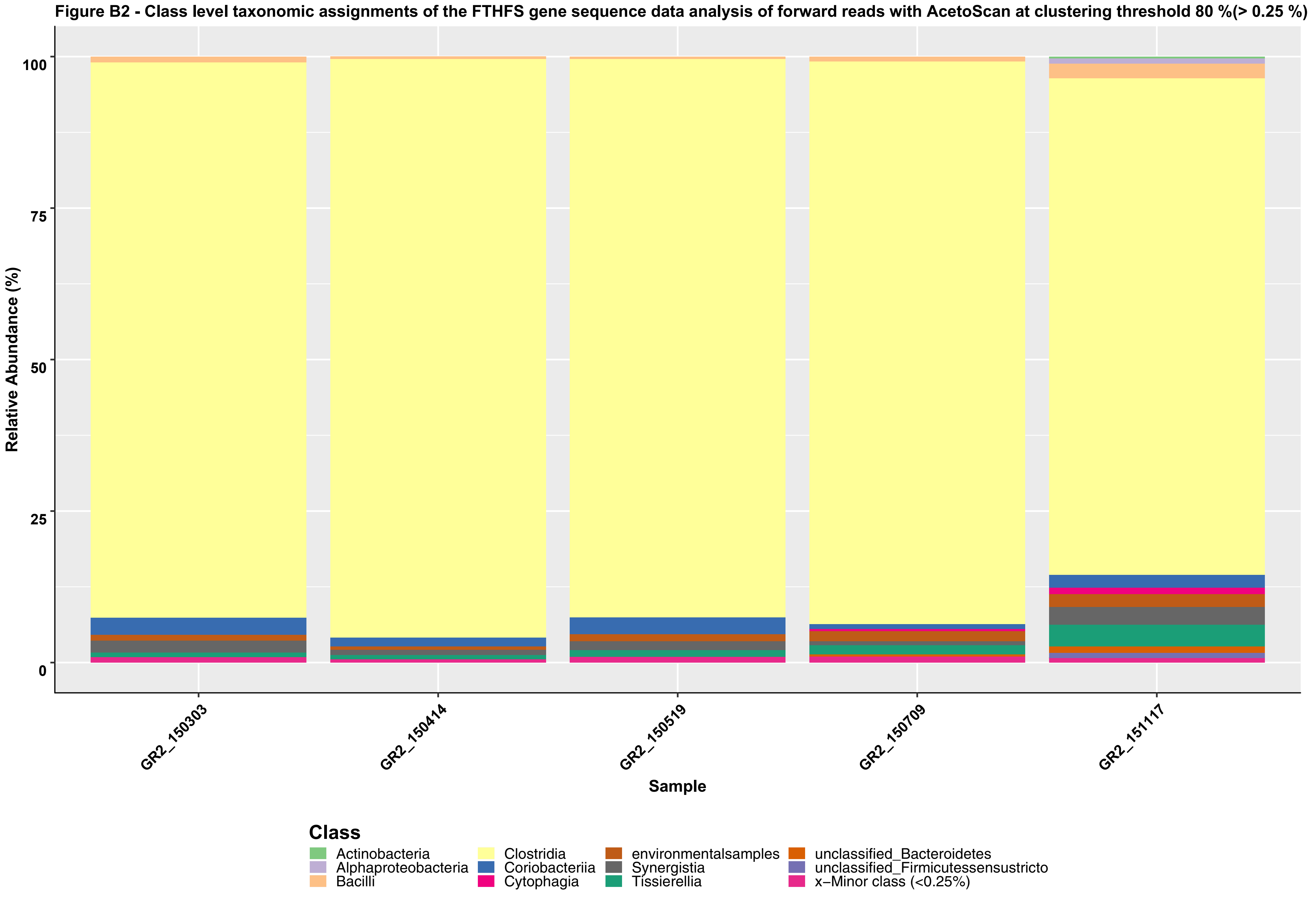
Figure B2 - Class level taxonomic assignments of the FTHFS gene sequence data analysis of forward reads with AcetoScan at clustering threshold 80 %(> 0.25 %)
Relative Abundance (%)
100
75
50
25
0
GR2_150303
GR2_150414
GR2_150519
GR2_150709
GR2_151117
Sample
Class
Actinobacteria
Alphaproteobacteria
Bacilli
Clostridia
Coriobacteriia
Cytophagia
environmentalsamples
Synergistia
Tissierellia
unclassified_Bacteroidetes
unclassified_Firmicutessensustricto
x–Minor class (<0.25%)

Figure B3 - Order level taxonomic assignments of the FTHFS gene sequence data analysis of forward reads with AcetoScan at clustering threshold 80 %(> 0.25 %)

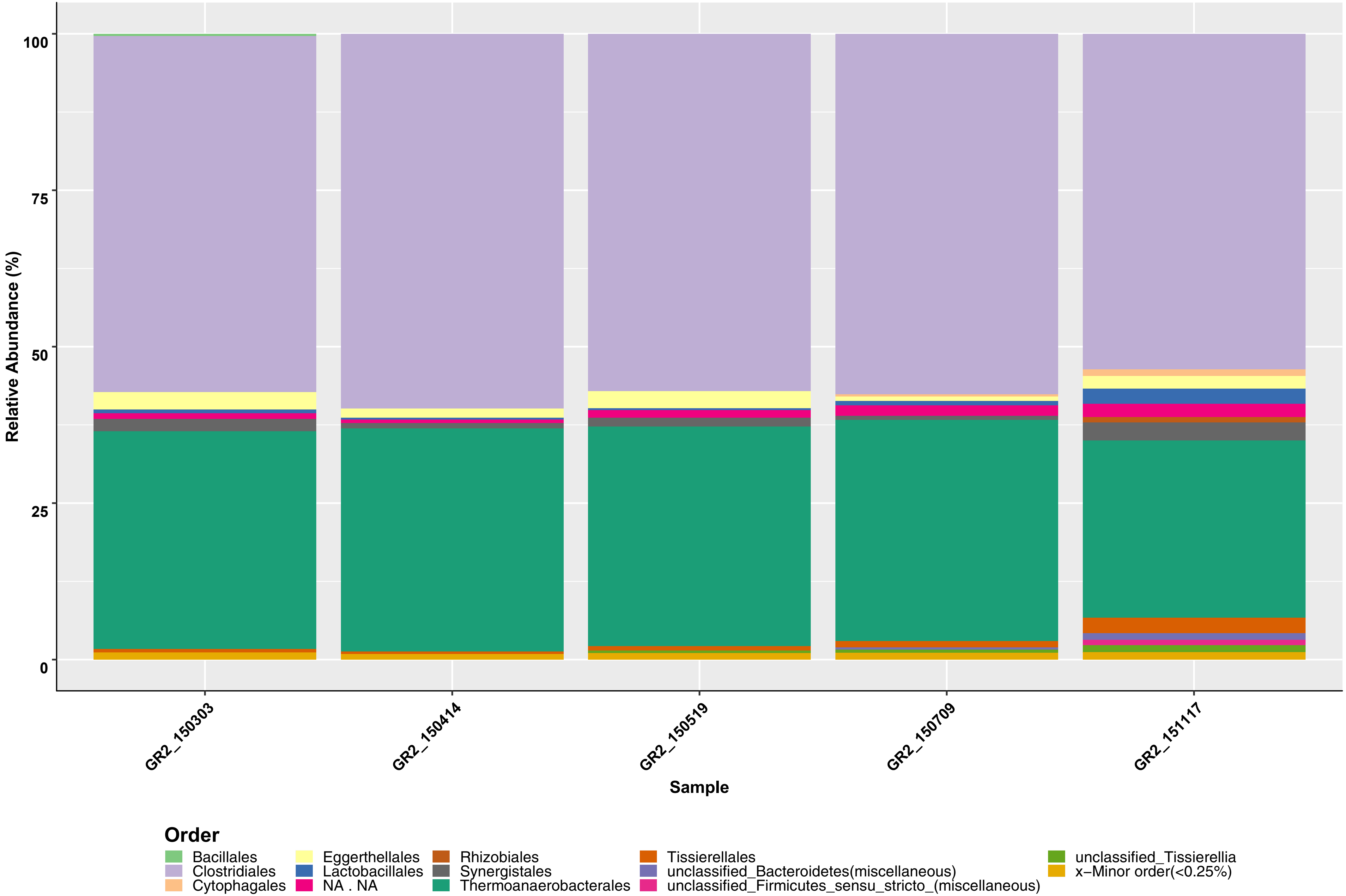

Figure B4 - Family level taxonomic assignments of the FTHFS gene sequence data analysis of forward reads with AcetoScan at clustering threshold 80 %(> 1 %)

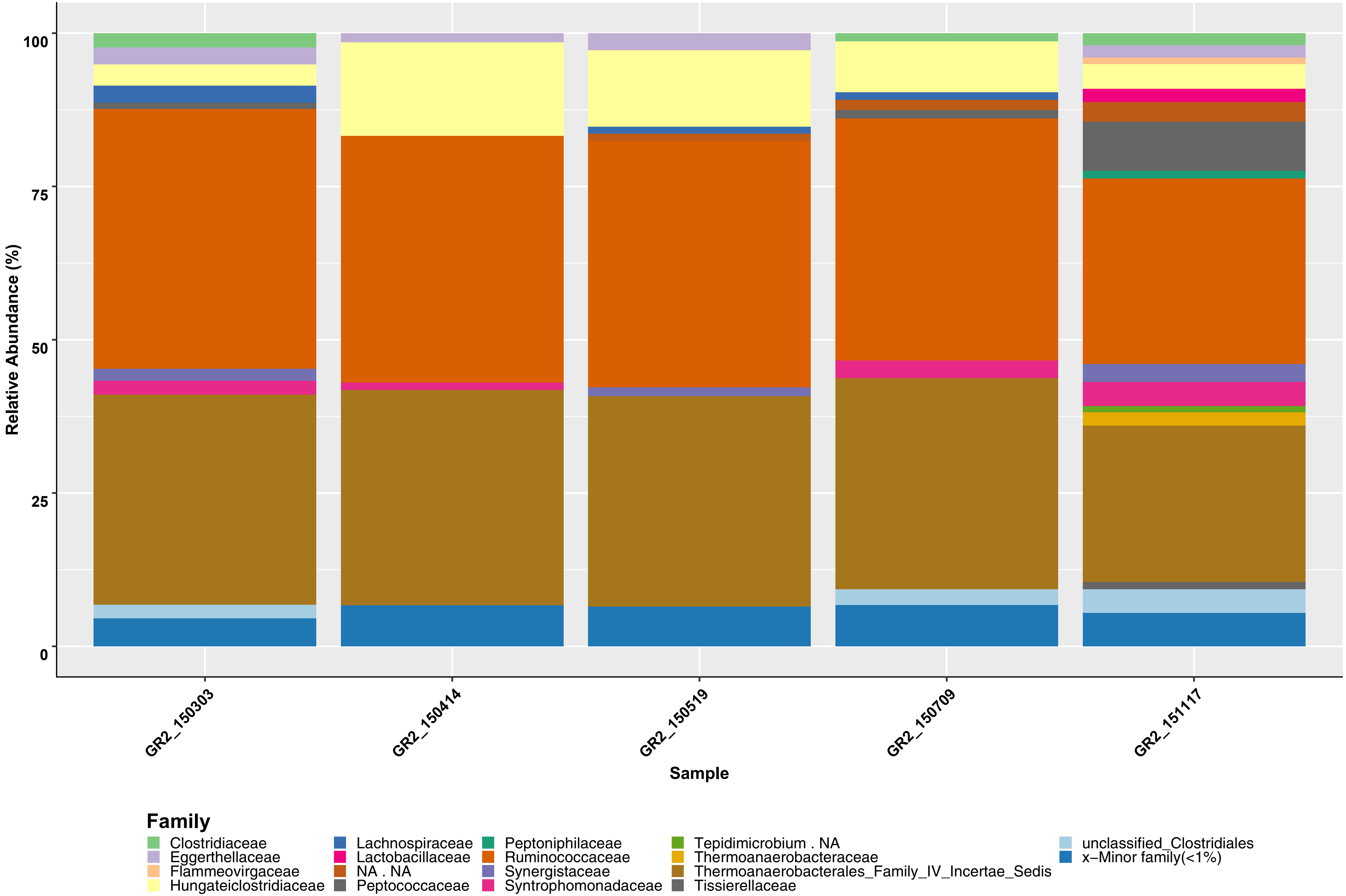

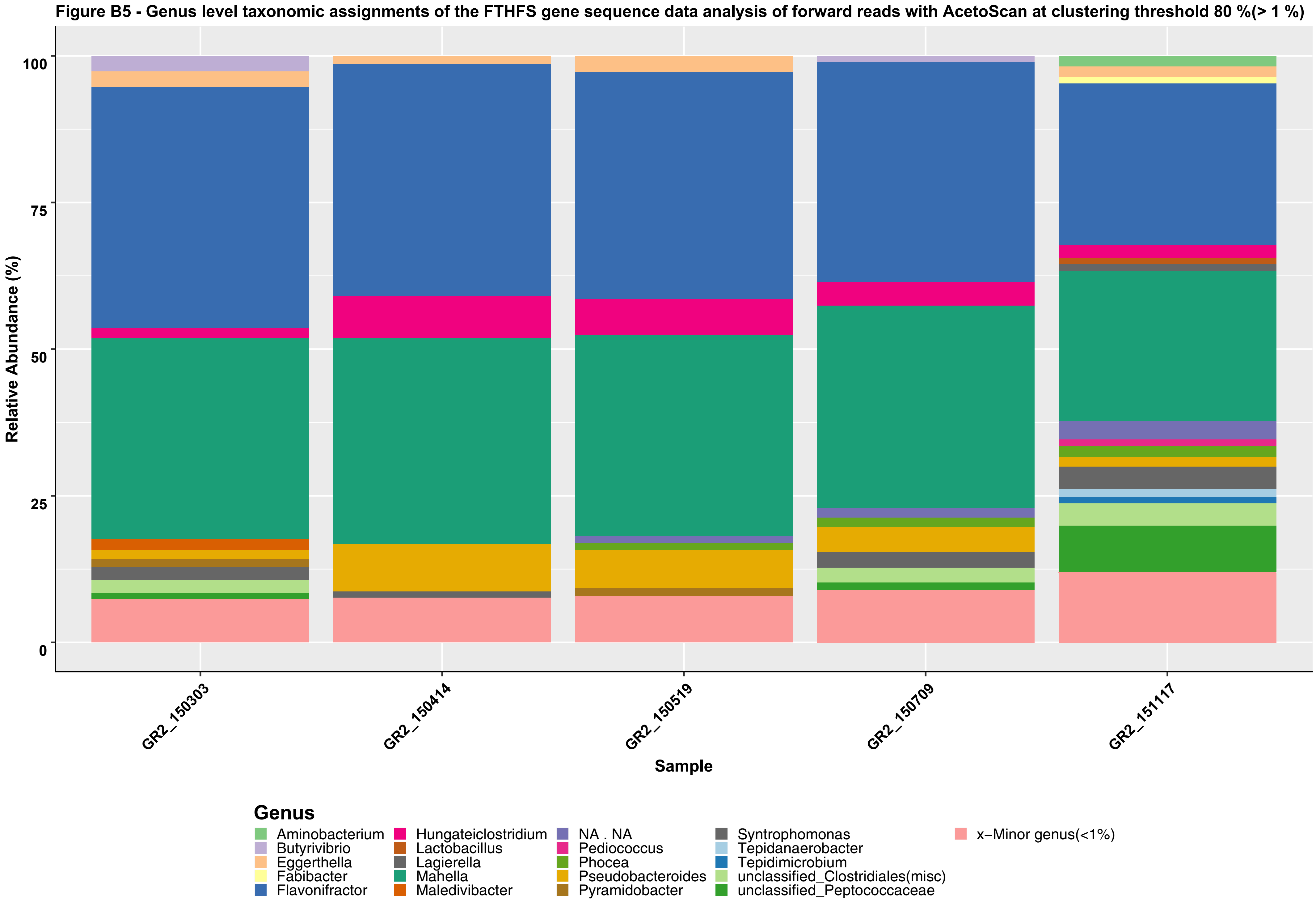
Figure B5 - Genus level taxonomic assignments of the FTHFS gene sequence data analysis of forward reads with AcetoScan at clustering threshold 80 %(> 1 %)
Relative Abundance (%)
100
75
50
25
0
GR2_150303
GR2_150414
GR2_150519
GR2_150709
GR2_151117
Sample
Genus
Aminobacterium
Butyrivibrio
Eggerthella
Fabibacter
Flavonifractor
Hungateiclostridium
Lactobacillus
Lagierella
Mahella
Maledivibacter
NA . NA
Pediococcus
Phocea
Pseudobacteroides
Pyramidobacter
Syntrophomonas
Tepidanaerobacter
Tepidimicrobium
unclassified_Clostridiales(misc)
unclassified_Peptococcaceae
x−Minor genus(<1%)

Figure B6 – Species level taxonomic assignments of the FTHFS gene sequence data analysis of forward reads with AcetoScan at clustering threshold 80 %(> 5 %)

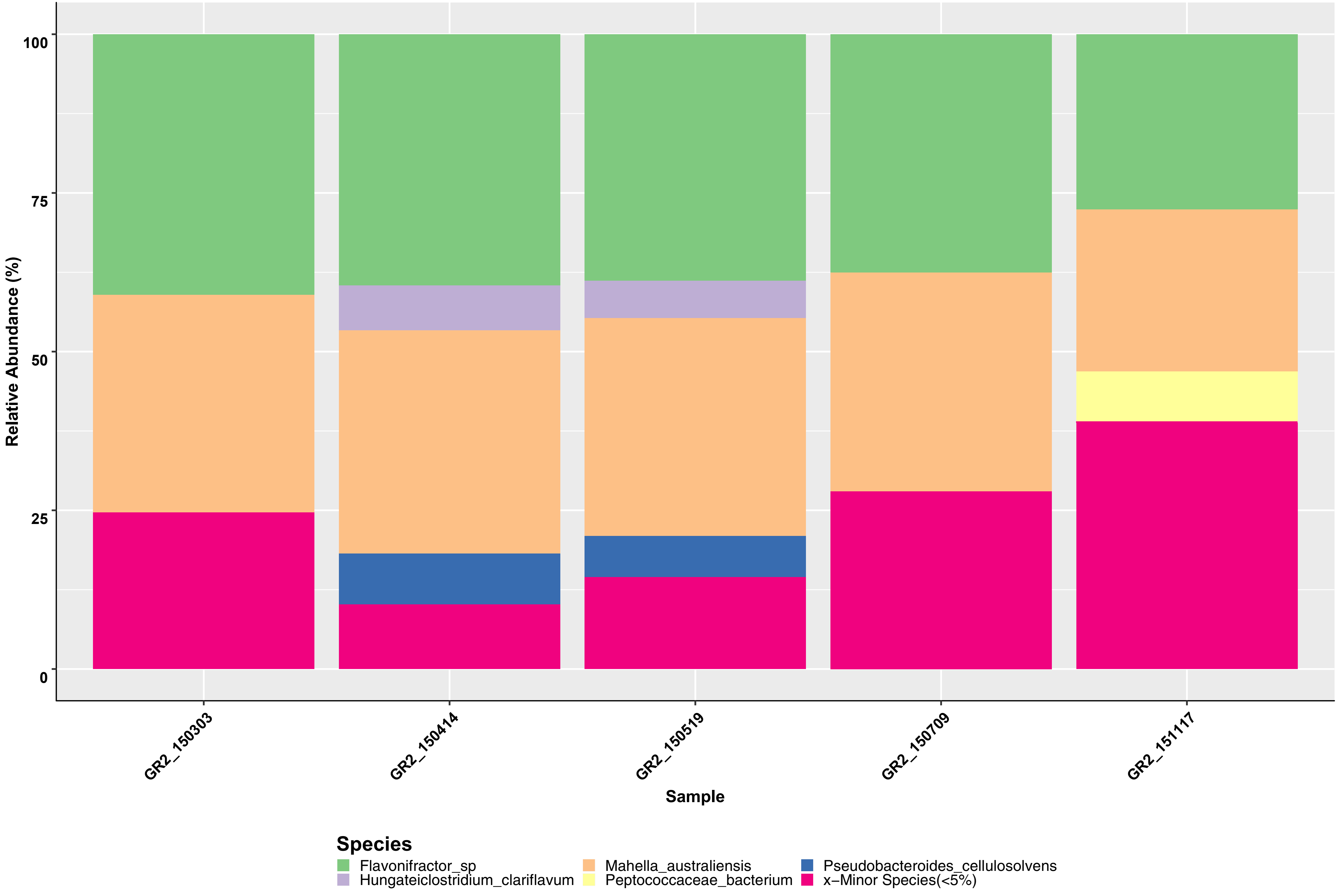

Supplement: Supplementary file 5 [file Data_Sheet_4.PDF]
