# Supplementary material for: High-Throughput Sequencing and Unsupervised Analysis of Formyltetrahydrofolate Synthetase (FTHFS) Gene Amplicons to Estimate Acetogenic Community Structure
Source: Front Microbiol. 2020 Aug 27;11:2066. doi: 10.3389/fmicb.2020.02066 (PMC7481360; doi:10.3389/fmicb.2020.02066)

Figure C1 – Phylum level taxonomic assignments of the FTHFS gene sequence data analysis of forward reads with AcetoScan at clustering threshold 100 %

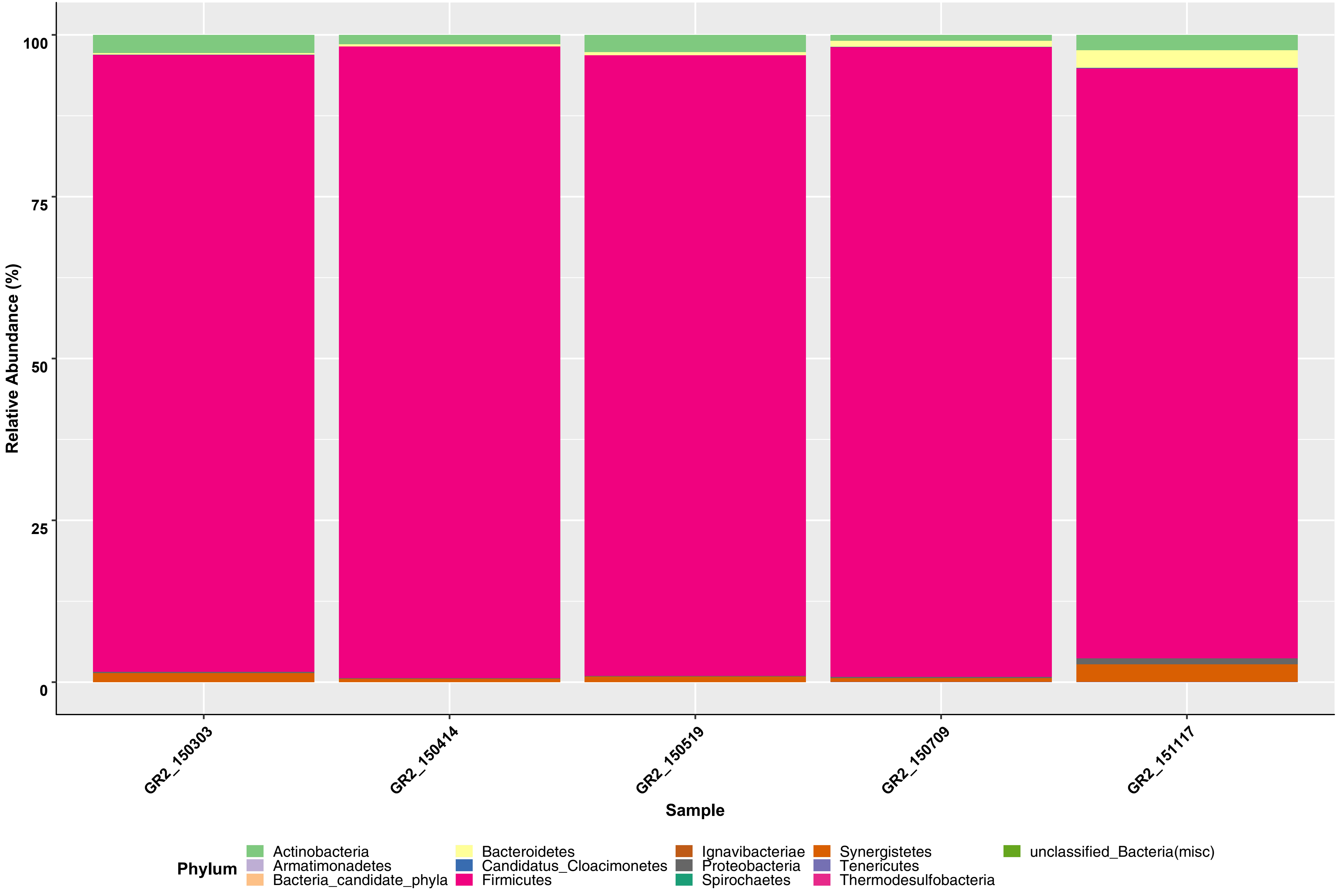

Figure C2 - Class level taxonomic assignments of the FTHFS gene sequence data analysis of forward reads with AcetoScan at clustering threshold 100 %(> 0.25 %)

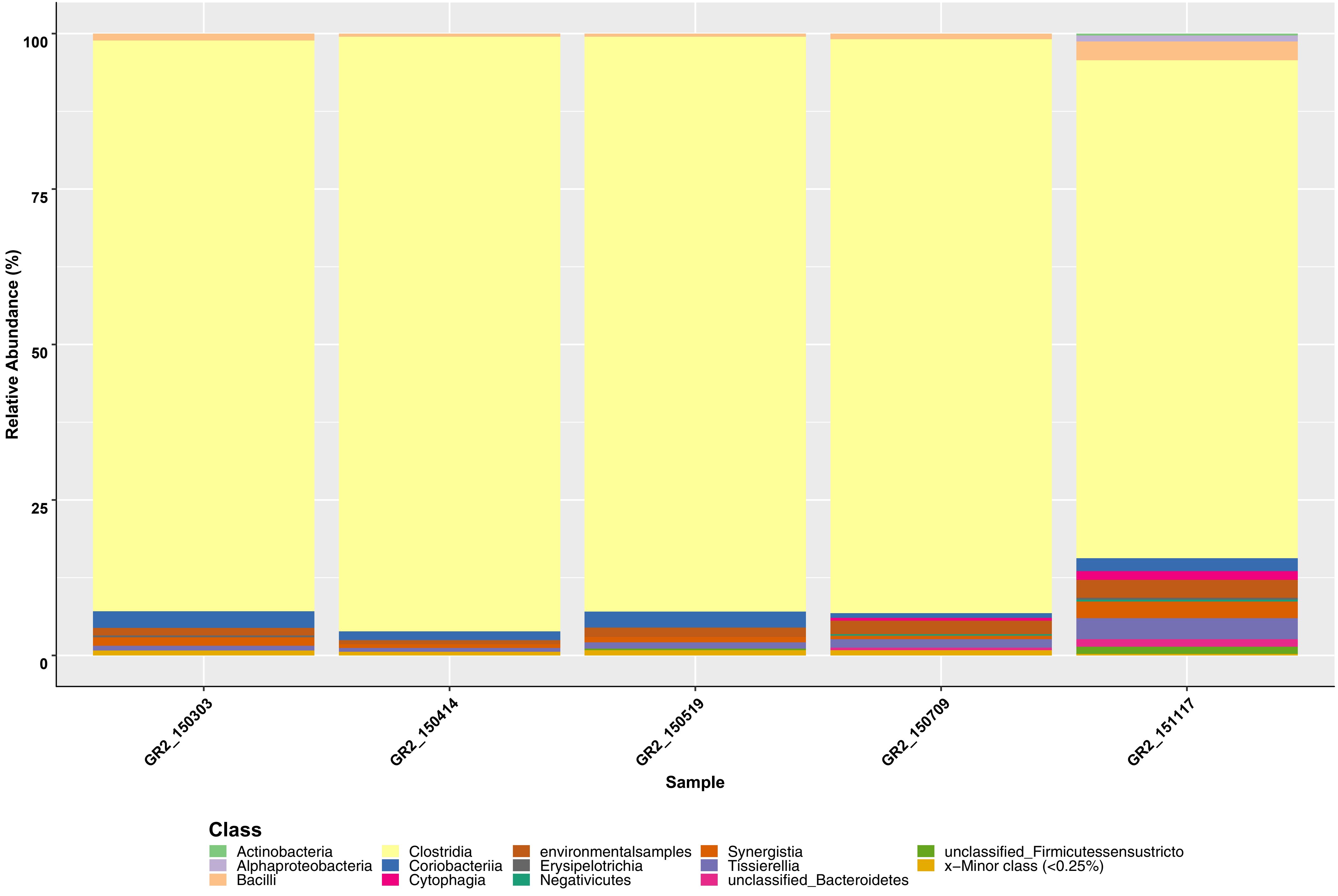

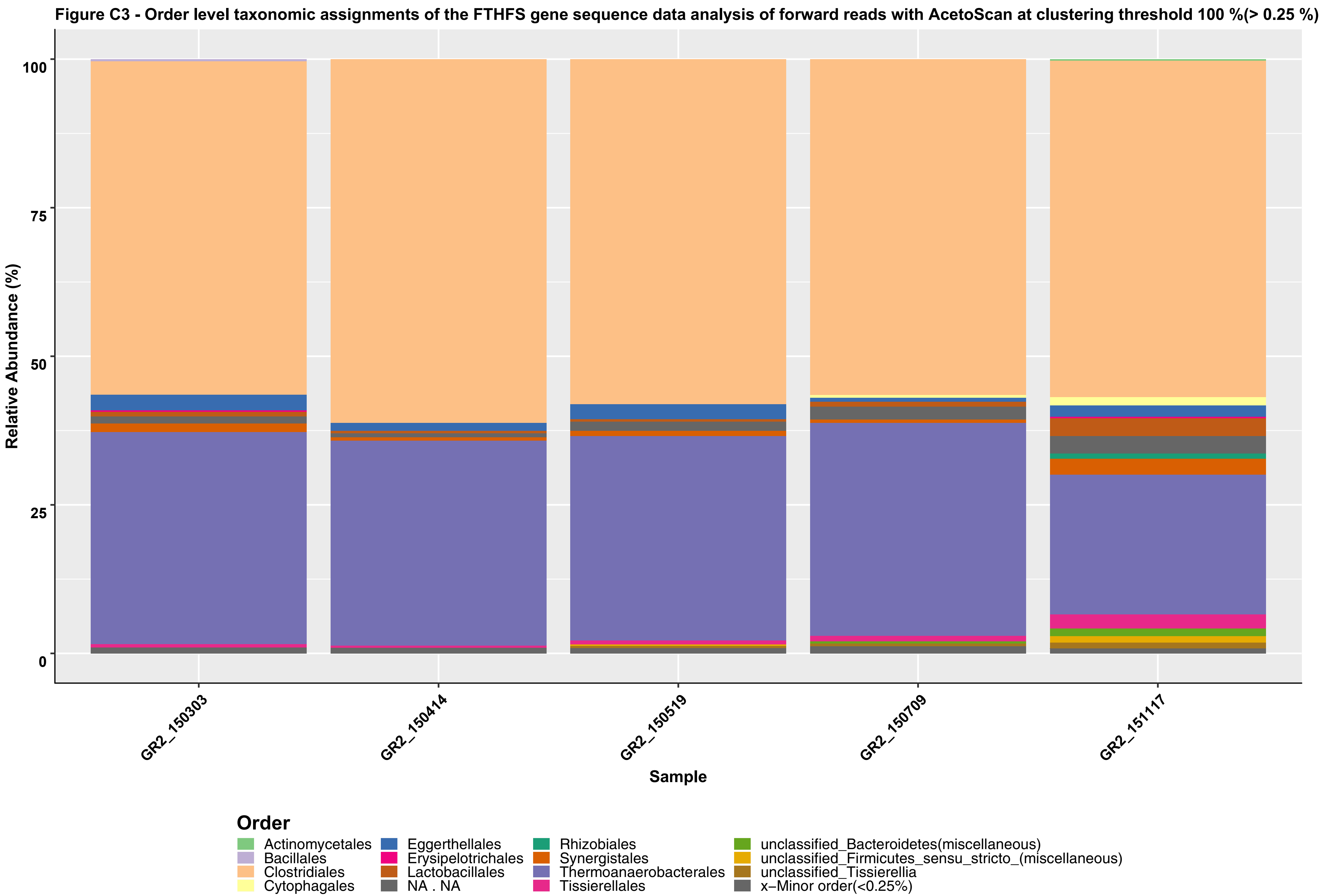
Figure C3 - Order level taxonomic assignments of the FTHFS gene sequence data analysis of forward reads with AcetoScan at clustering threshold 100 %(> 0.25 %)
Relative Abundance (%)
100
75
50
25
0
GR2_150303
GR2_150414
GR2_150519
GR2_150709
GR2_151117
Sample
Order
Actinomycetales
Bacillales
Clostridiales
Cytophagales
Eggerthellales
Erysipelotrichales
Lactobacillales
NA . NA
Rhizobiales
Synergistales
Thermoanaerobacterales
Tissierellales
unclassified_Bacteroidetes(miscellaneous)
unclassified_Firmicutes_sensu_stricto_(miscellaneous)
unclassified_Tissierellia
x−Minor order(<0.25%)

Figure C4 - Family level taxonomic assignments of the FTHFS gene sequence data analysis of forward reads with AcetoScan at clustering threshold 100 %(> 1 %)

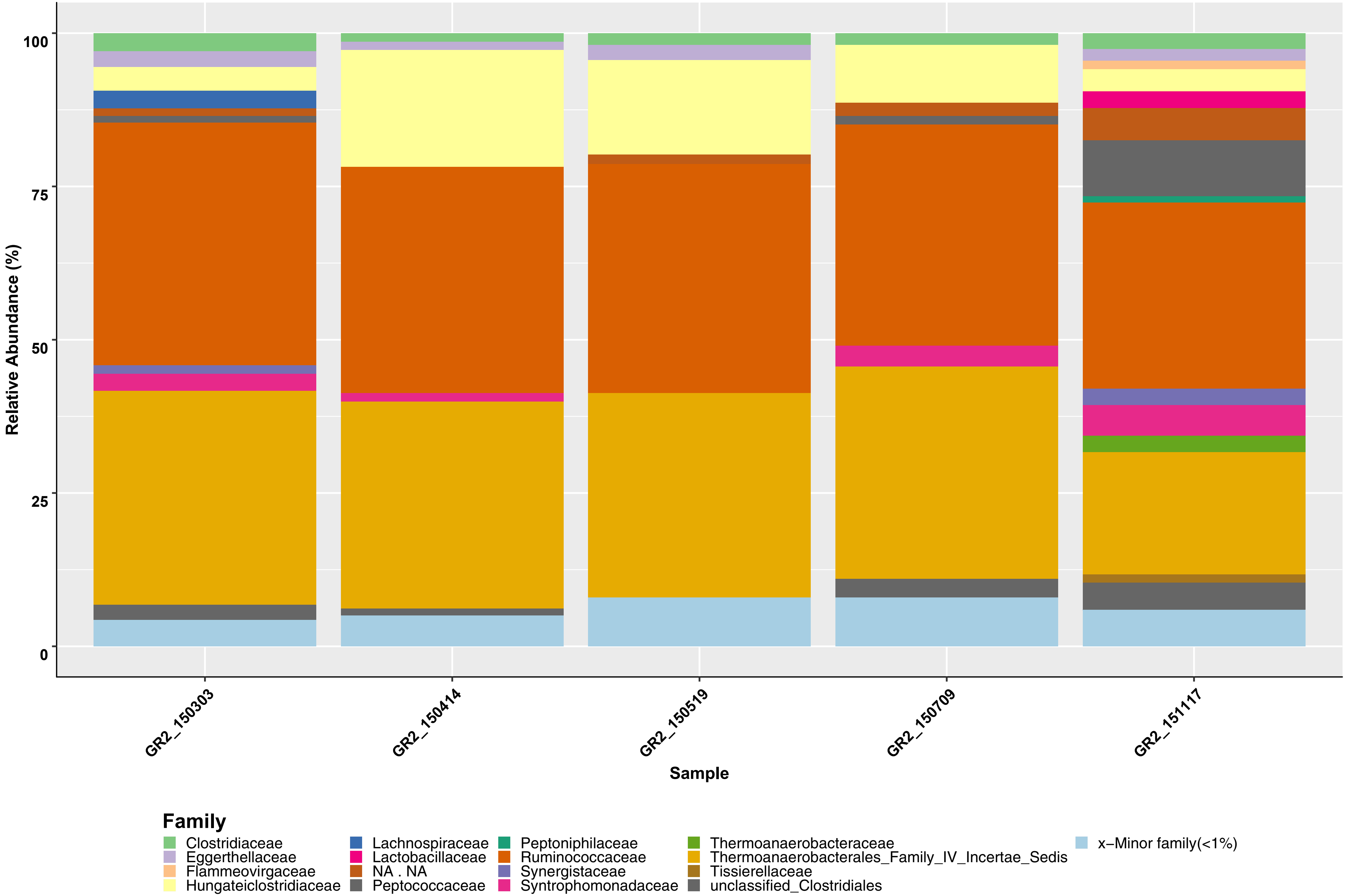

Figure C5 - Genus level taxonomic assignments of the FTHFS gene sequence data analysis of forward reads with AcetoScan at clustering threshold 100 % (> 1 %)

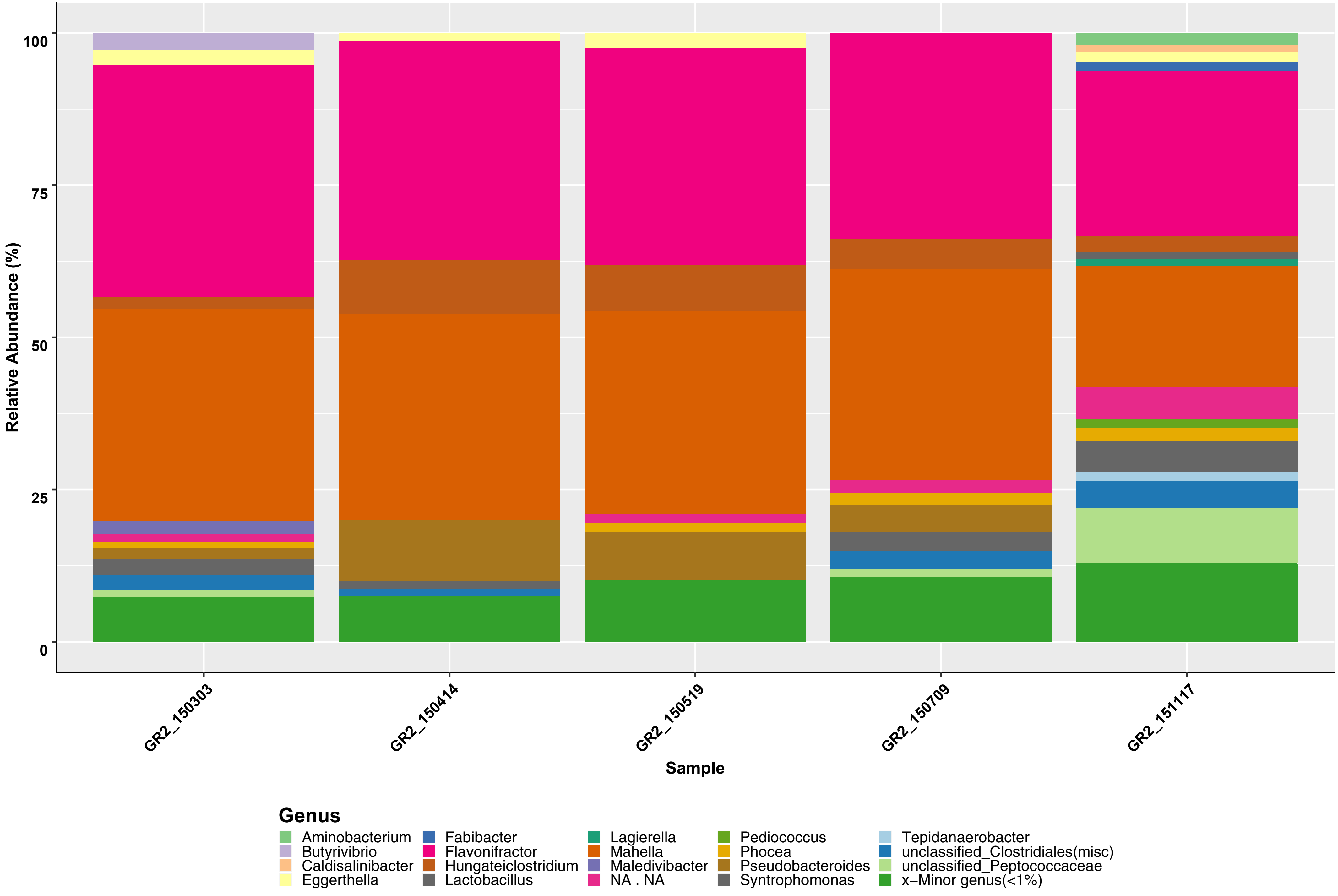

Figure C6 – Species level taxonomic assignments of the FTHFS gene sequence data analysis of forward reads with AcetoScan at clustering threshold 100 %(> 5 %)

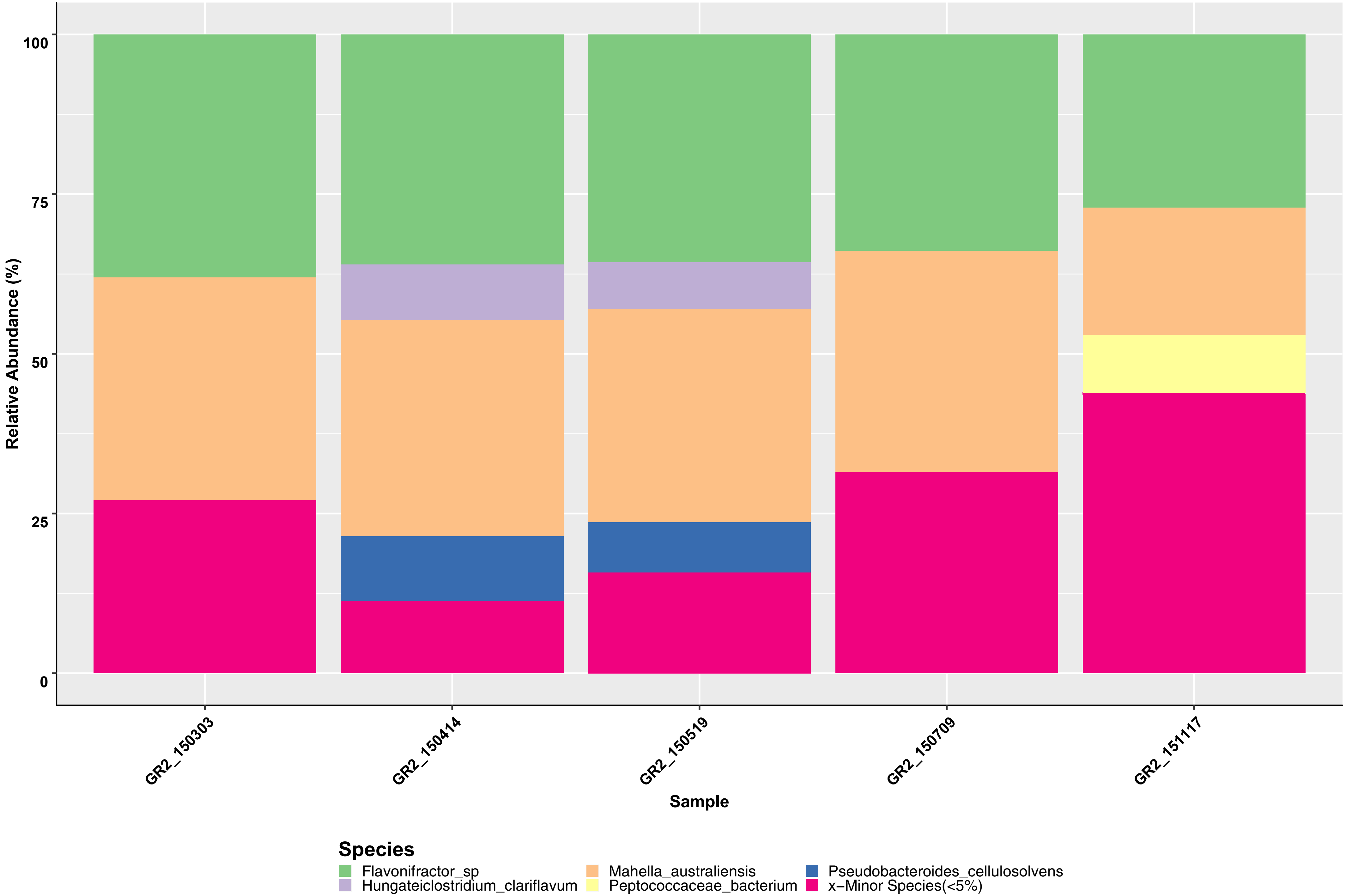

Supplement: Supplementary file 6 [file Data_Sheet_5.PDF]
