# Supplementary material for: High-Throughput Sequencing and Unsupervised Analysis of Formyltetrahydrofolate Synthetase (FTHFS) Gene Amplicons to Estimate Acetogenic Community Structure
Source: Front Microbiol. 2020 Aug 27;11:2066. doi: 10.3389/fmicb.2020.02066 (PMC7481360; doi:10.3389/fmicb.2020.02066)
Supplement: Supplementary file 7 [file Data_Sheet_6.PDF]

Figure D1 – Phylum level taxonomic assignments of the FTHFS gene sequence data analysis of reverse reads with AcetoScan at clustering threshold 80 %

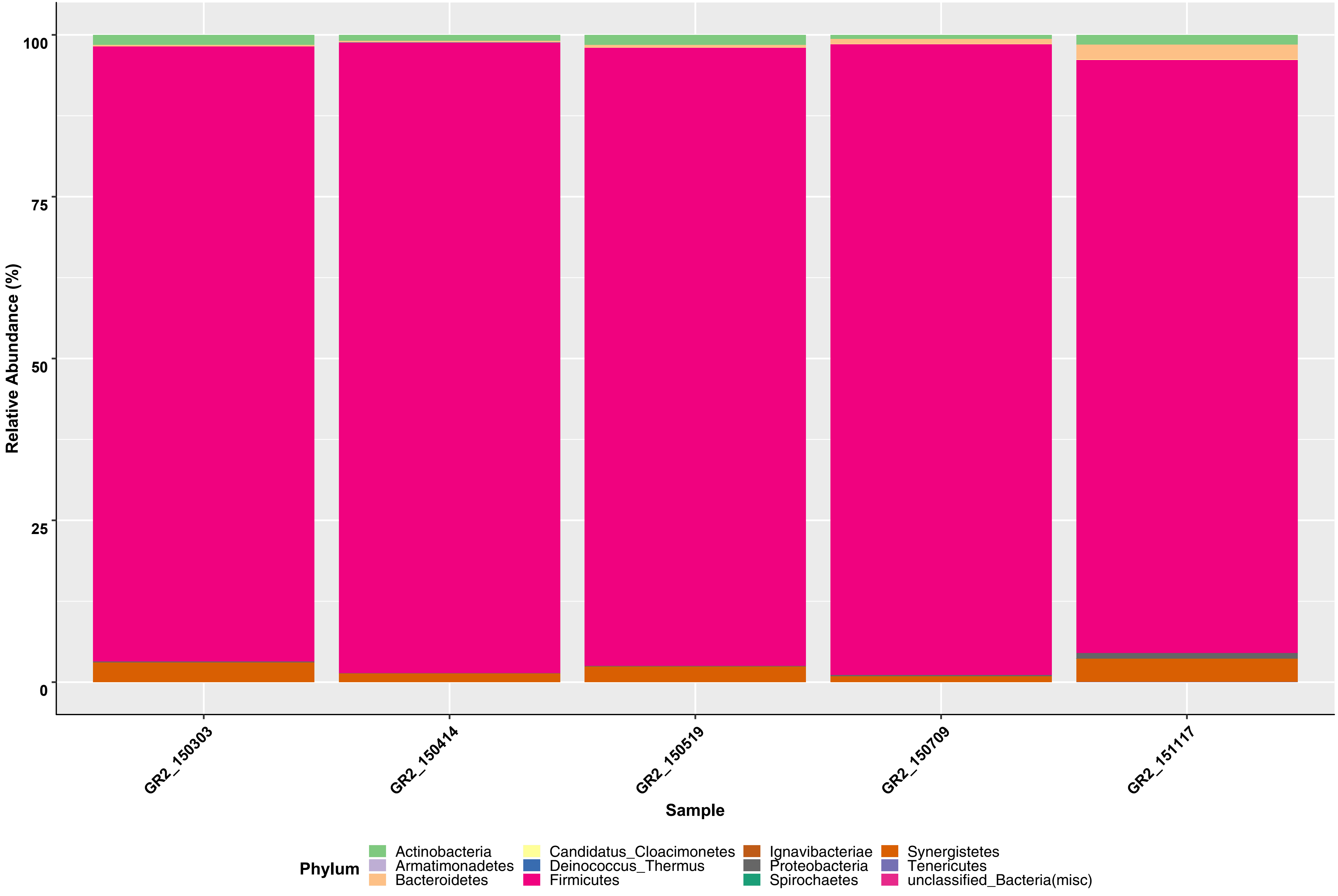

Figure D2 - Class level taxonomic assignments of the FTHFS gene sequence data analysis of reverse reads with AcetoScan at clustering threshold 80 %(> 0.25 %)

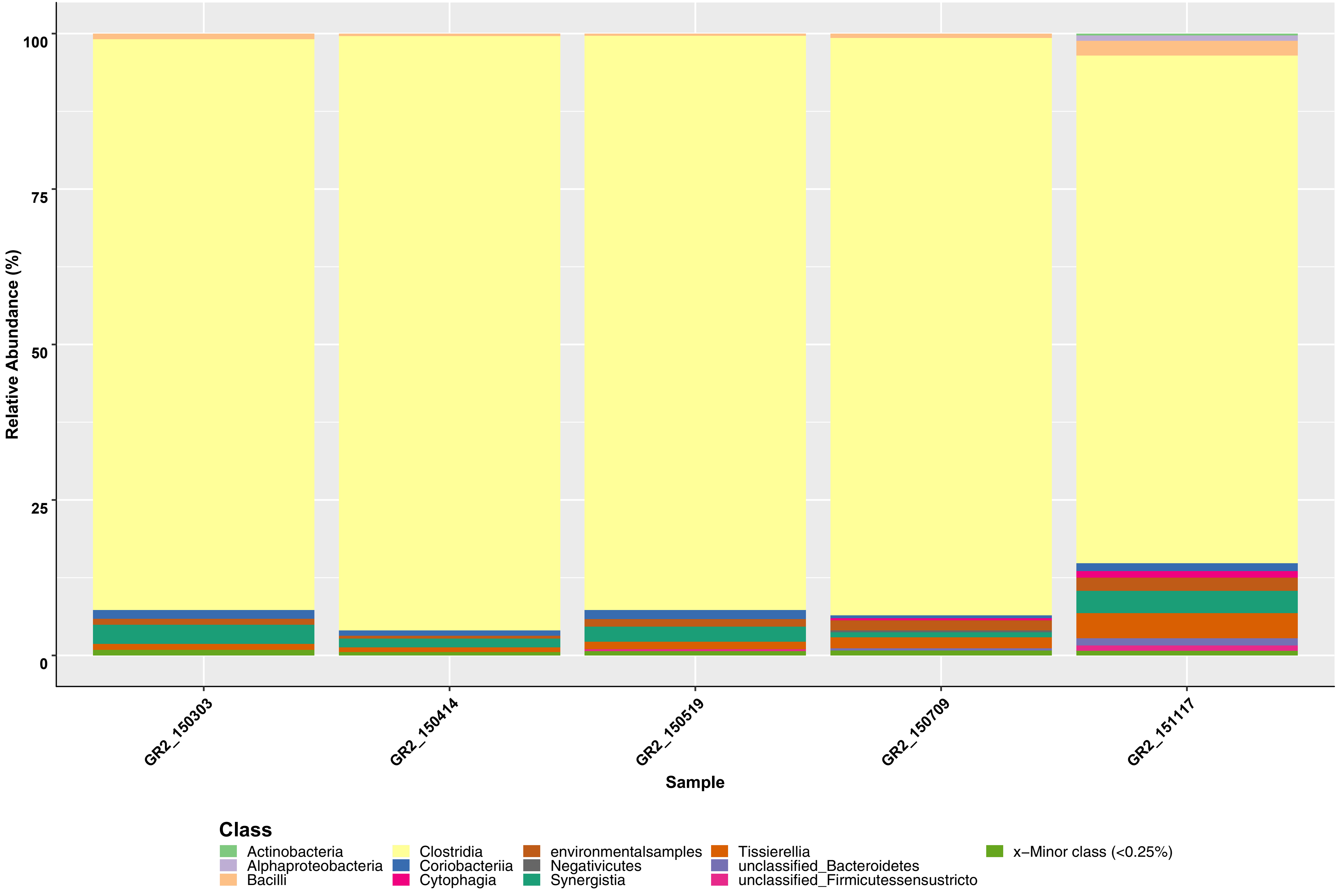

Figure D3 - Order level taxonomic assignments of the FTHFS gene sequence data analysis of reverse reads with AcetoScan at clustering threshold 80 %(> 0.25 %)

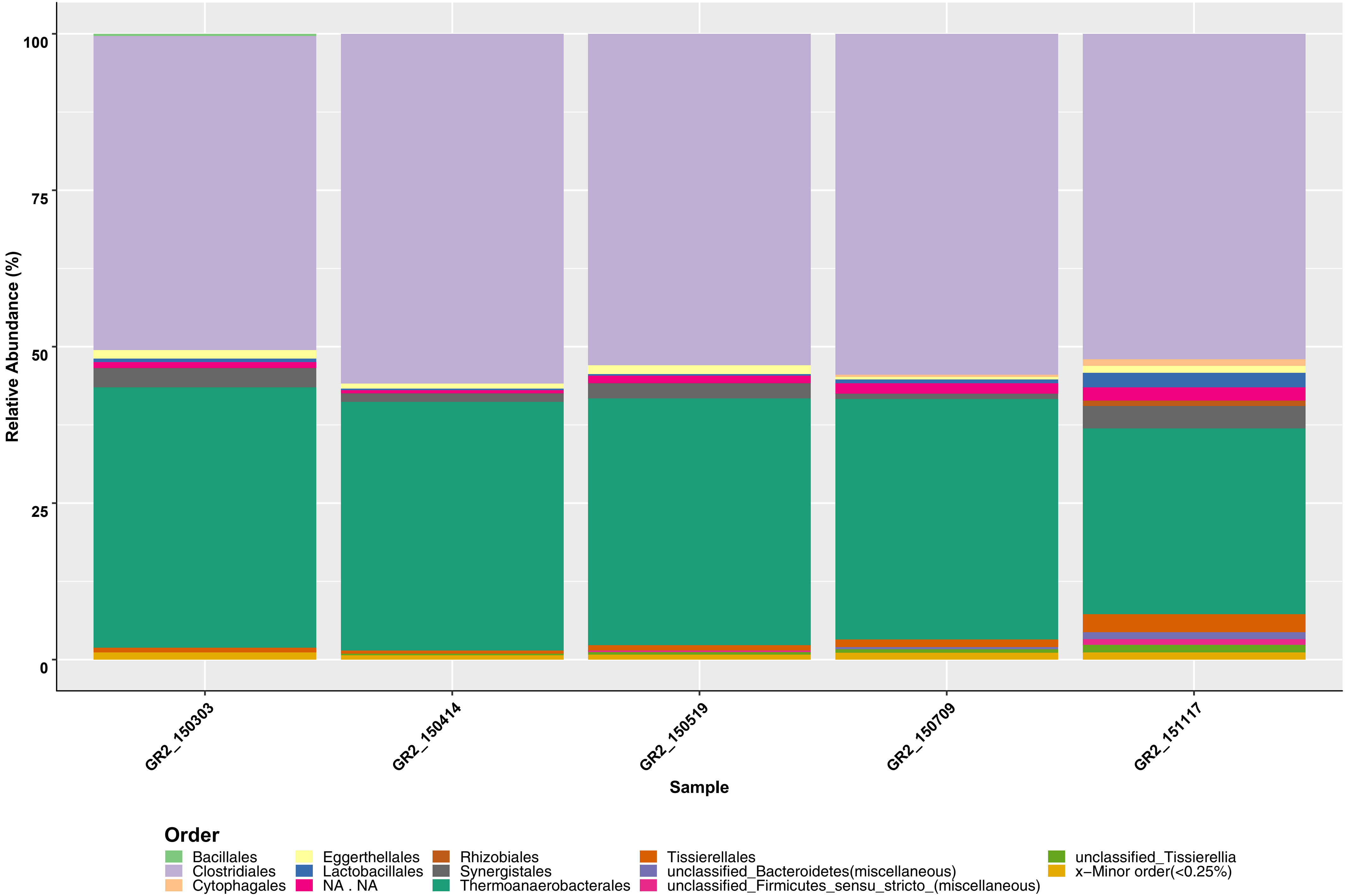

Figure D4 - Family level taxonomic assignments of the FTHFS gene sequence data analysis of reverse reads with AcetoScan at clustering threshold 80 %(> 1 %)

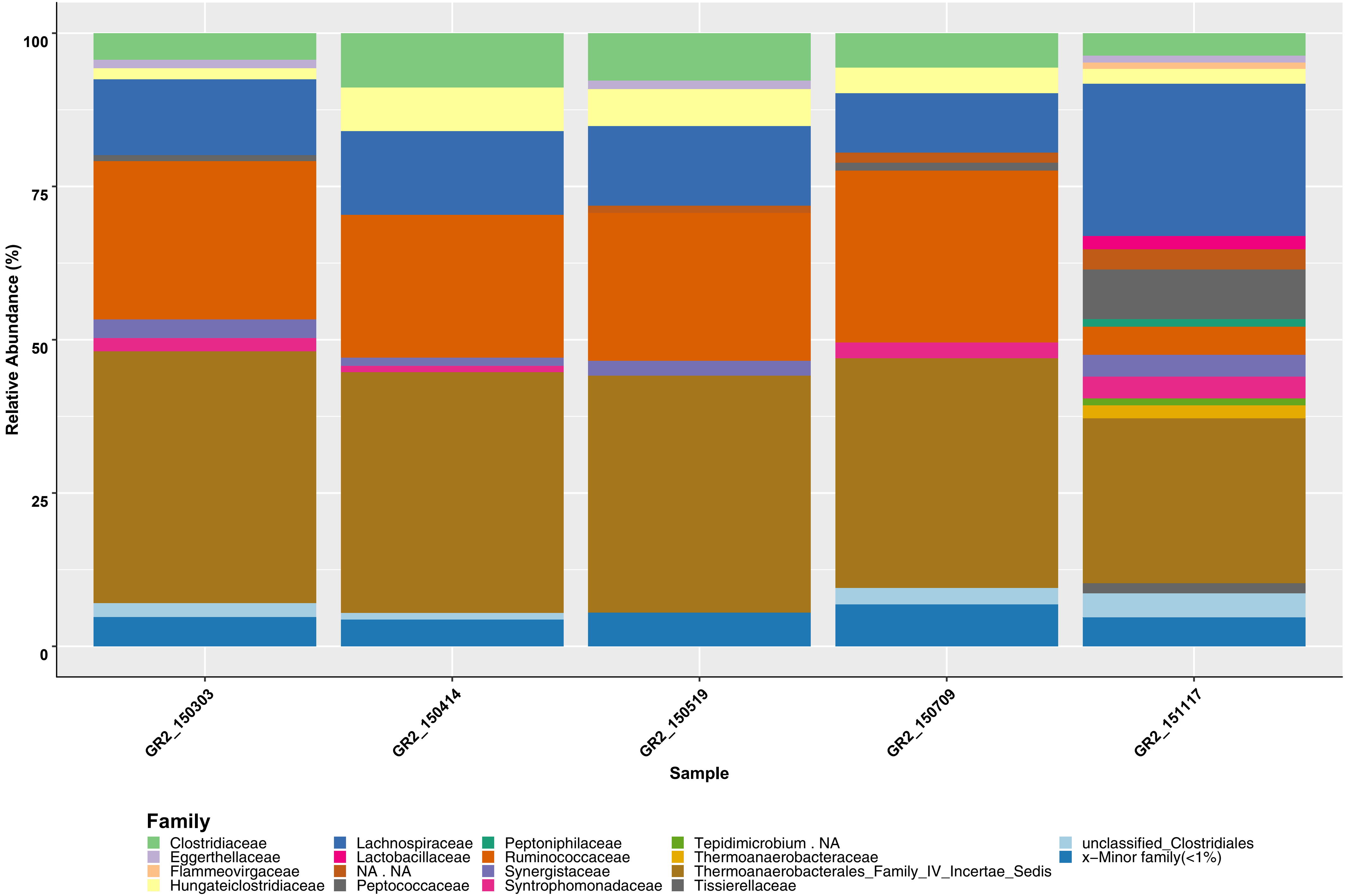

Figure D5 - Genus level taxonomic assignments of the FTHFS gene sequence data analysis of reverse reads with AcetoScan at clustering threshold 80 %(> 1 %)

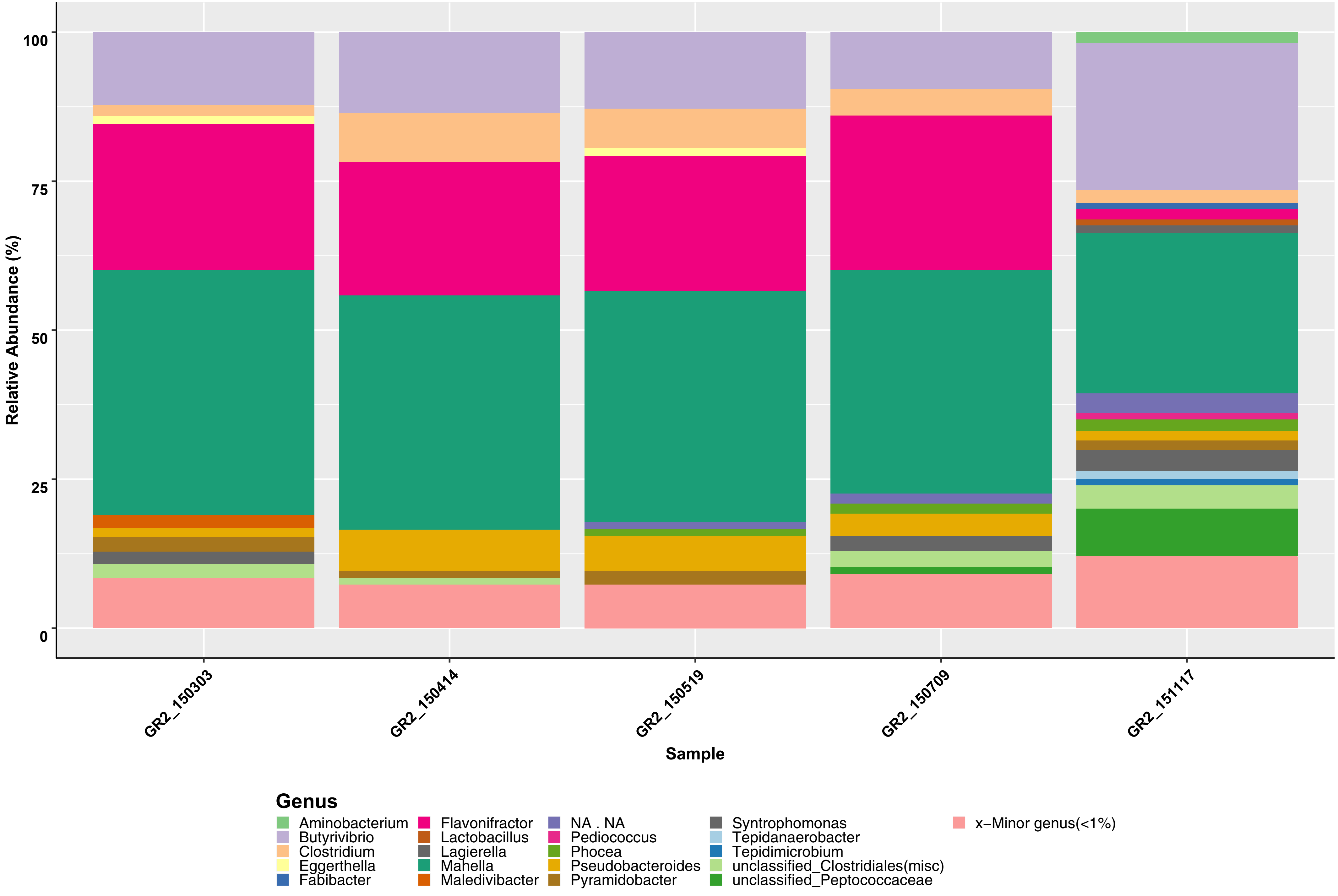

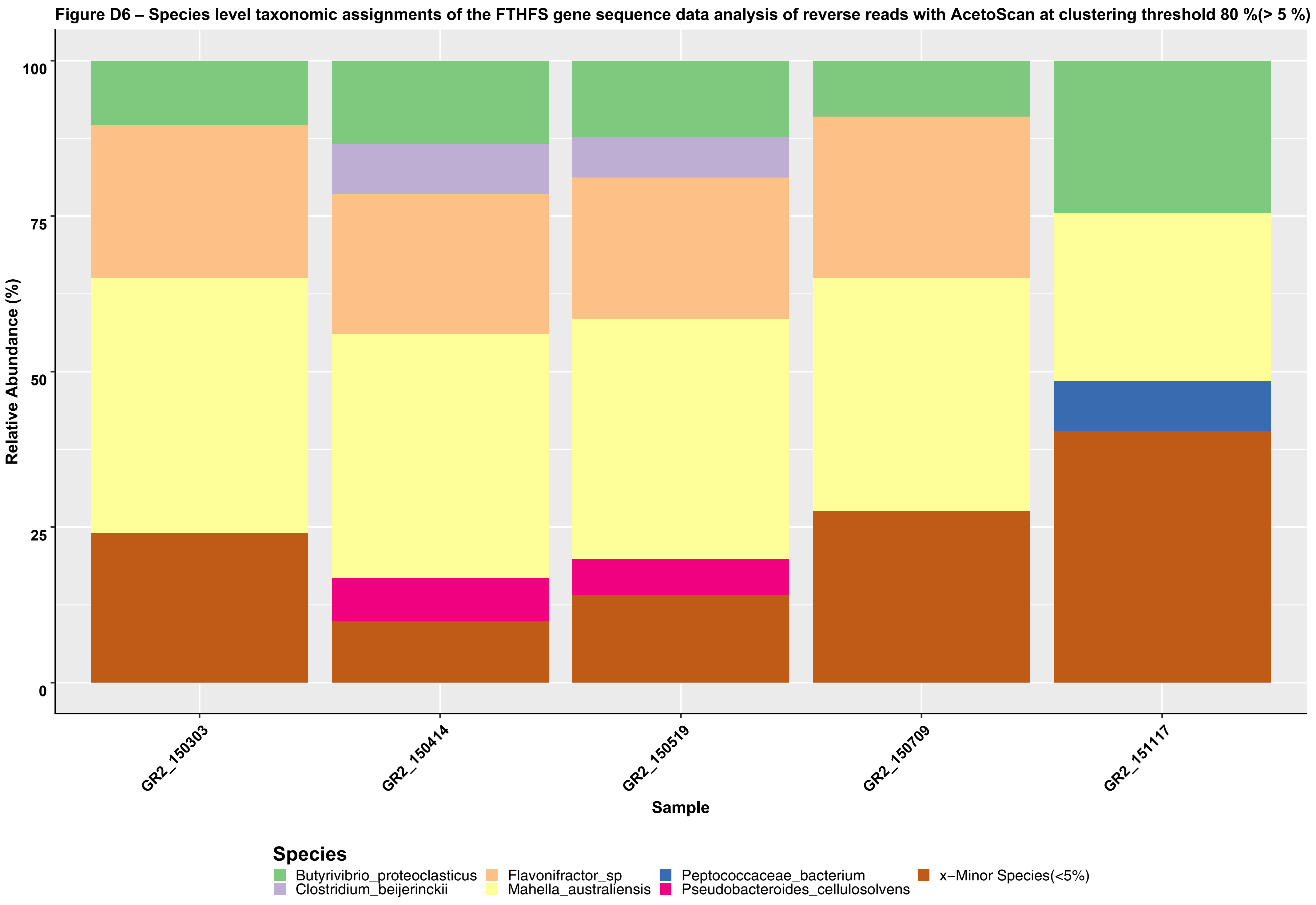
Figure D6 – Species level taxonomic assignments of the FTHFS gene sequence data analysis of reverse reads with AcetoScan at clustering threshold 80 %(> 5 %)
100
75
50
25
0
Relative Abundance (%)
GR2_150303
GR2_150414
GR2_150519
GR2_150709
GR2_151117
Sample
Species
Butyrivibrio_proteoclasticus
Clostridium_beijerinckii
Flavonifractor_sp
Mahella_australiensis
Peptococcaceae_bacterium
Pseudobacteroides_cellulosolvens
x−Minor Species(<5%)
