# Supplementary material for: High-Throughput Sequencing and Unsupervised Analysis of Formyltetrahydrofolate Synthetase (FTHFS) Gene Amplicons to Estimate Acetogenic Community Structure
Source: Front Microbiol. 2020 Aug 27;11:2066. doi: 10.3389/fmicb.2020.02066 (PMC7481360; doi:10.3389/fmicb.2020.02066)

Figure E1 – Phylum level taxonomic assignments of the FTHFS gene sequence data analysis of reverse reads with AcetoScan at clustering threshold 100 %

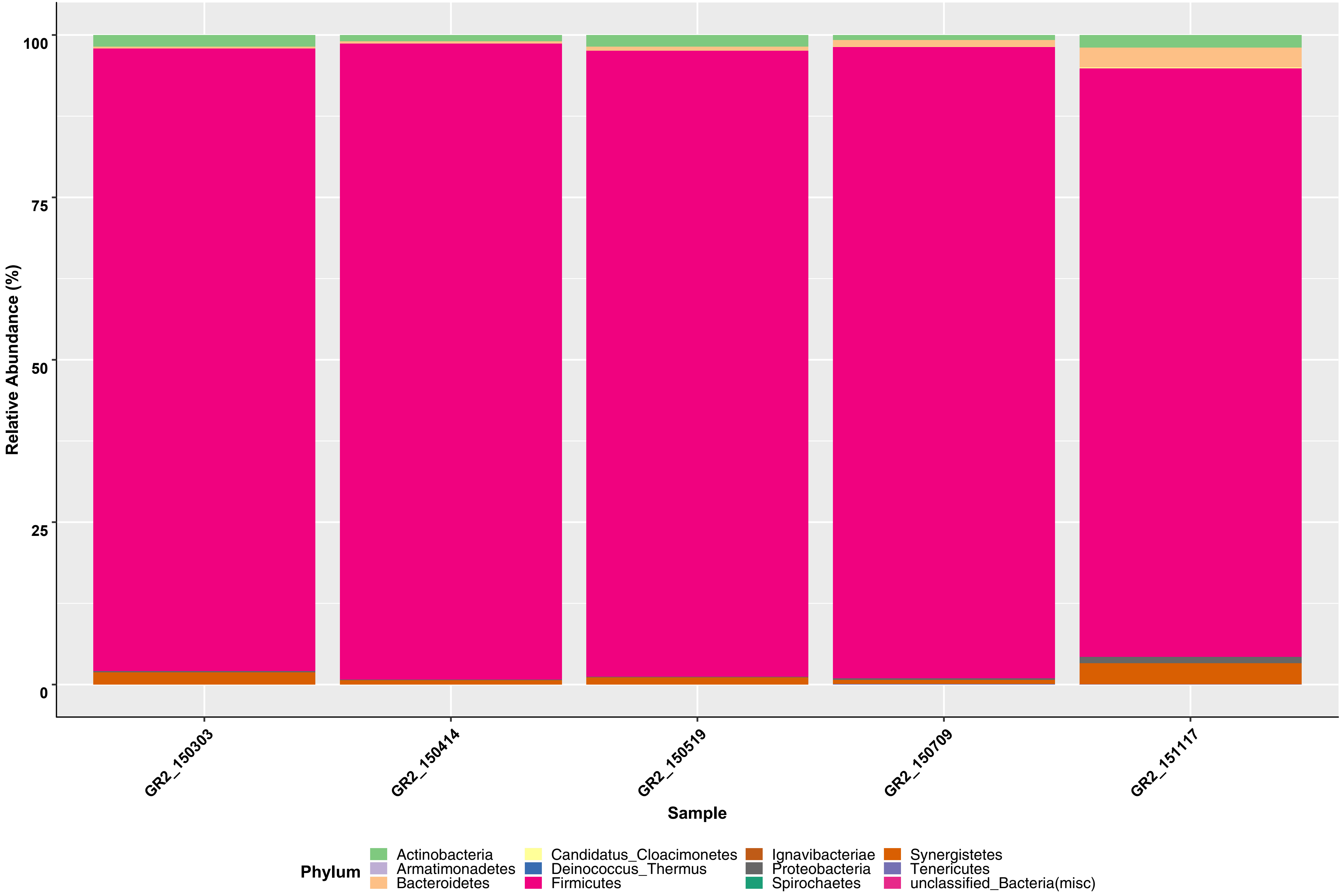

Figure E2 - Class level taxonomic assignments of the FTHFS gene sequence data analysis of reverse reads with AcetoScan at clustering threshold 100 %(> 0.25 %)

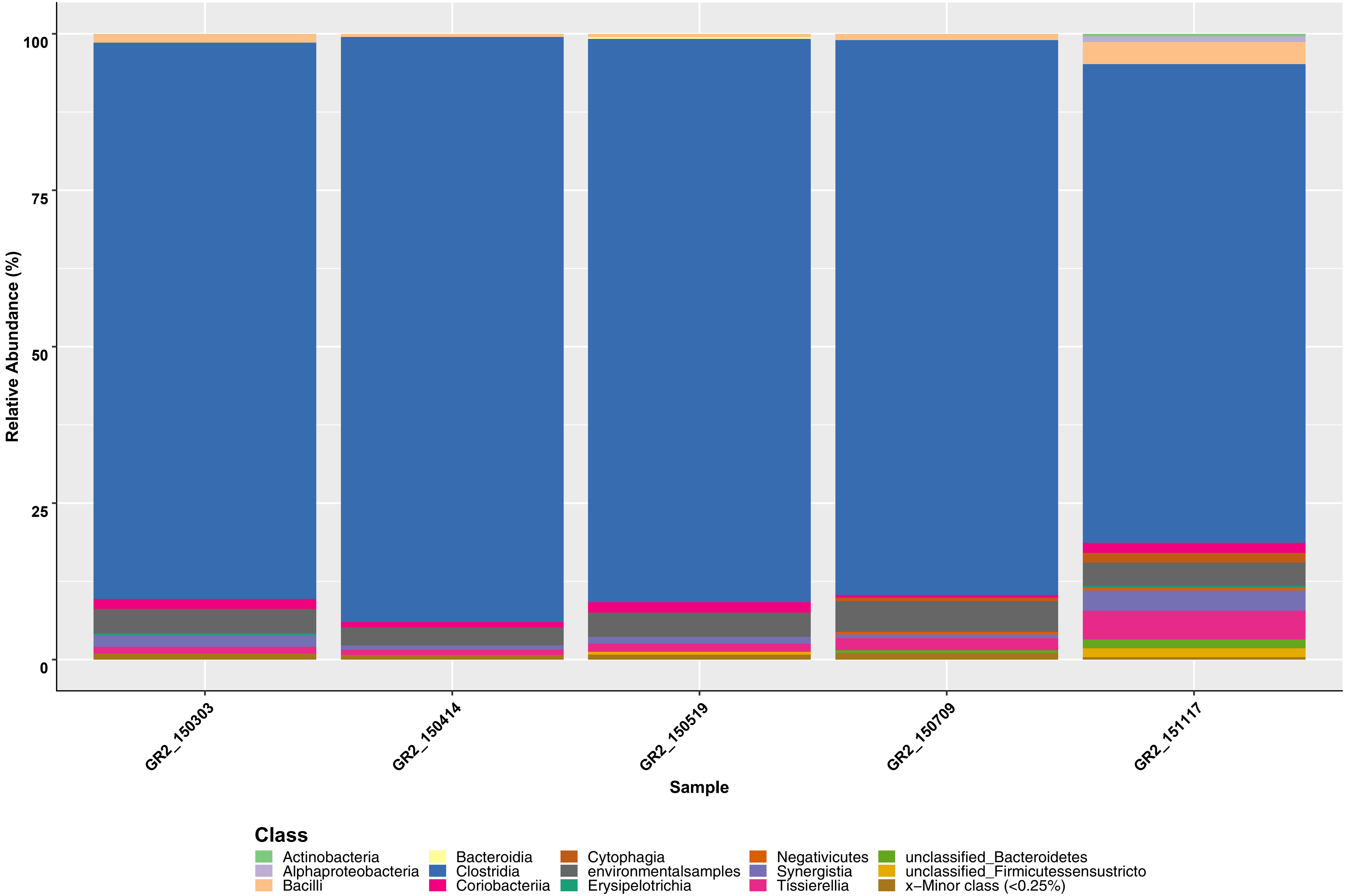

Figure E3 - Order level taxonomic assignments of the FTHFS gene sequence data analysis of reverse reads with AcetoScan at clustering threshold 100 %(> 0.25 %)

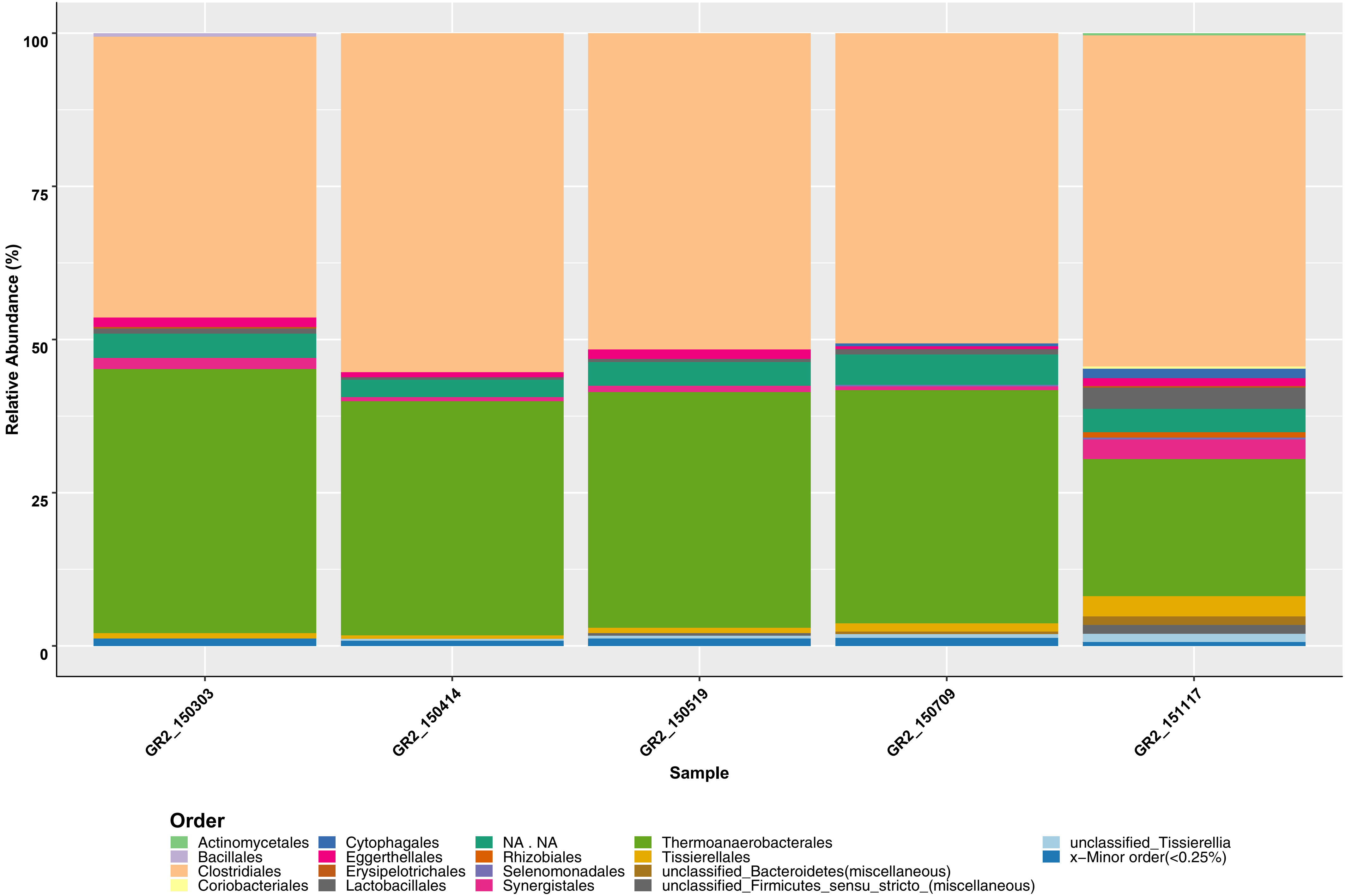

Figure E4 - Family level taxonomic assignments of the FTHFS gene sequence data analysis of reverse reads with AcetoScan at clustering threshold 100 %(> 1 %)

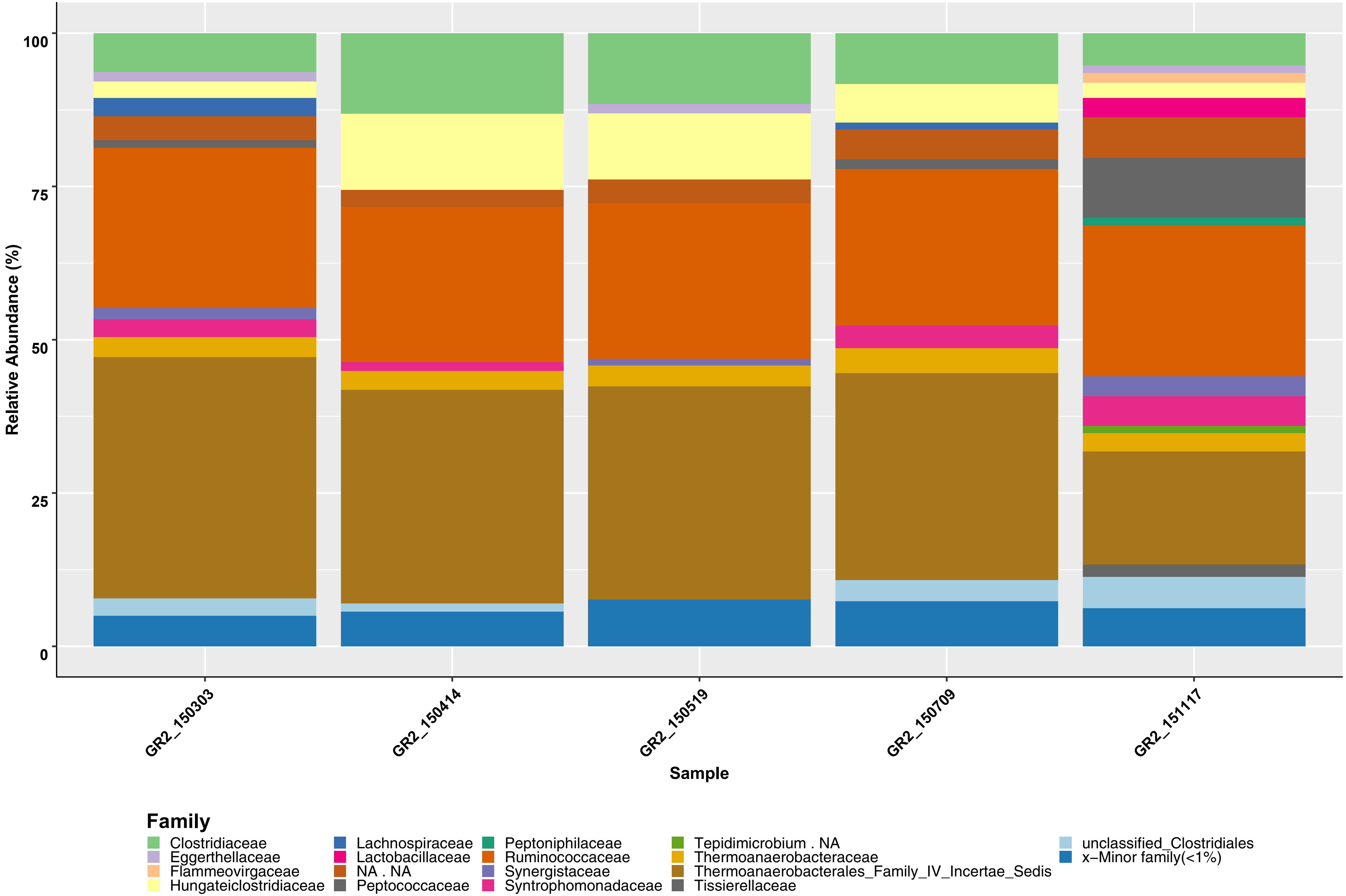

Figure E5 - Genus level taxonomic assignments of the FTHFS gene sequence data analysis of reverse reads with AcetoScan at clustering threshold 100 %(> 1 %)

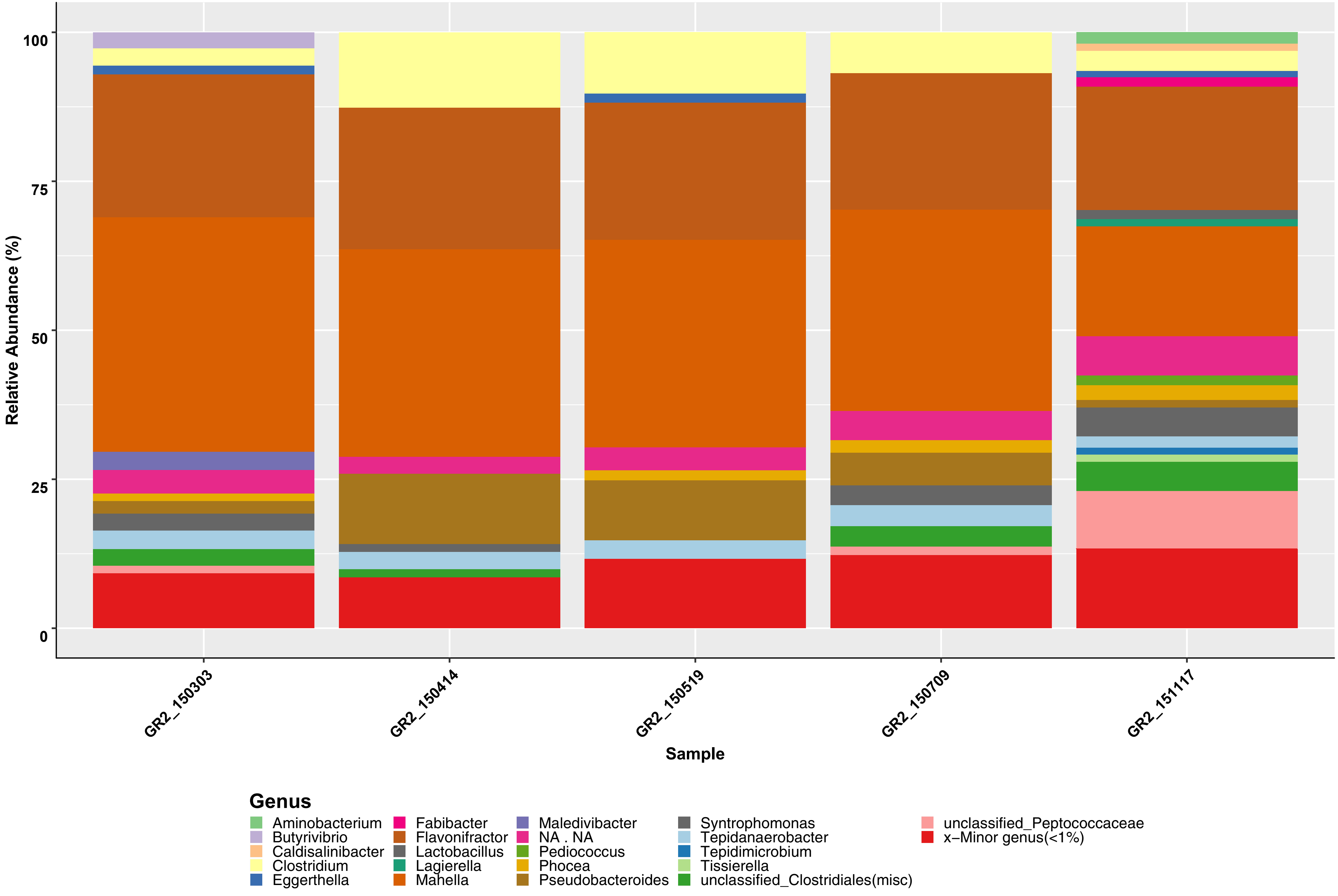

Figure E6 – Species level taxonomic assignments of the FTHFS gene sequence data analysis of reverse reads with AcetoScan at clustering threshold 100 %(> 5 %)

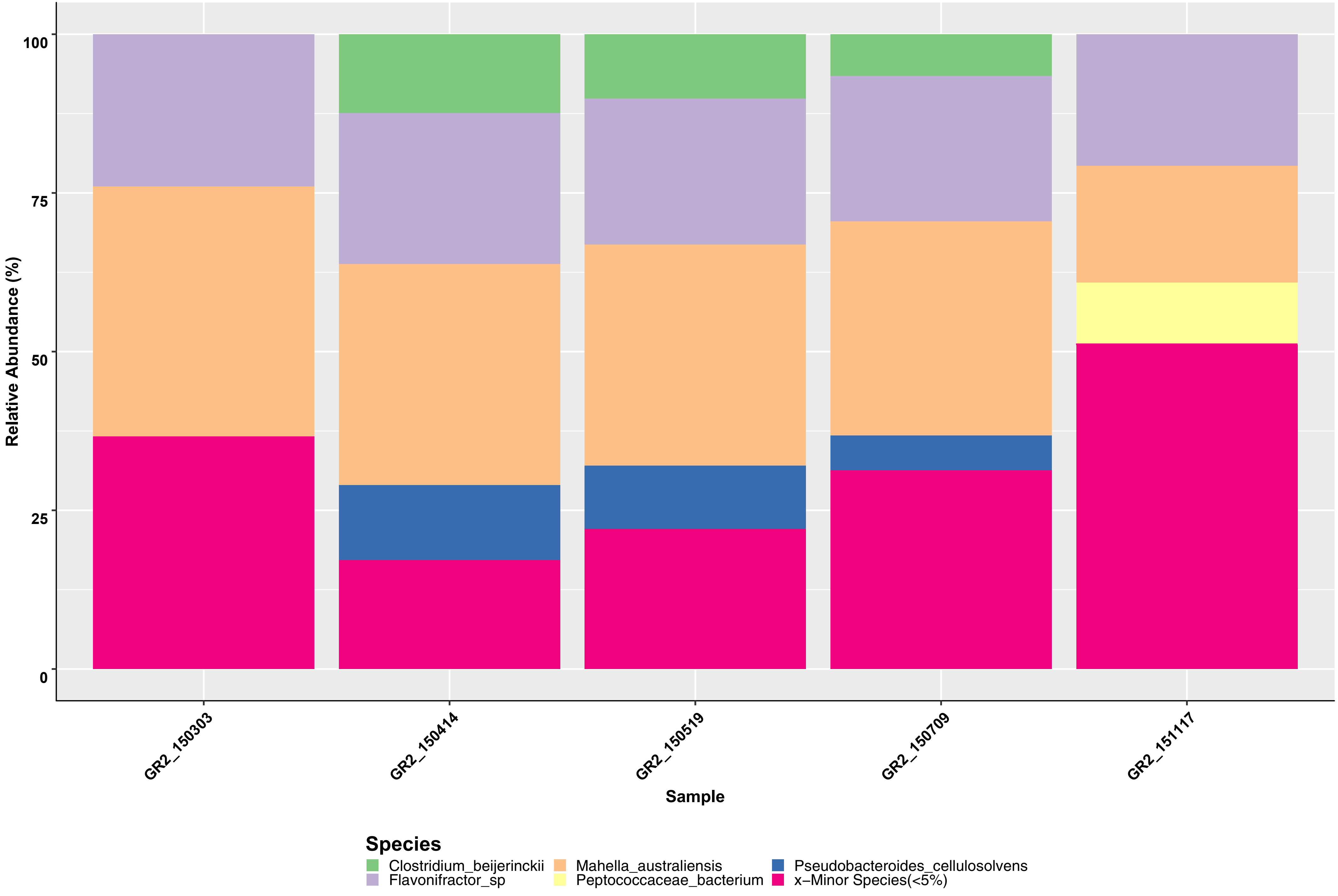

Supplement: Supplementary file 8 [file Data_Sheet_7.PDF]
